# Detecting Cage Crossing and Filling Clusters of Magnesium and Carbon Atoms in Zeolite SSZ-13 with Atom Probe Tomography

**DOI:** 10.1021/jacsau.2c00296

**Published:** 2022-10-14

**Authors:** Sophie H. van Vreeswijk, Matteo Monai, Ramon Oord, Joel E. Schmidt, Andrei N. Parvulescu, Irina Yarulina, Lukasz Karwacki, Jonathan D. Poplawsky, Bert M. Weckhuysen

**Affiliations:** † Inorganic Chemistry and Catalysis group, Debye Institute for Nanomaterials Science, Utrecht University, Universiteitsweg 99, Utrecht 3854 CG, The Netherlands; ‡ Center for Nanophase Materials Sciences, Oak Ridge National Laboratory, Oak Ridge, Tennessee 37831, United States; § BASF, Carl-Bosch-Straße 38, 67063 Ludwigshafen am Rhein, Germany

**Keywords:** Zeolites, Methanol-to-Hydrocarbons, Atom Probe Tomography, *Operando* UV−Vis Spectroscopy, *Operando* X-ray Diffraction

## Abstract

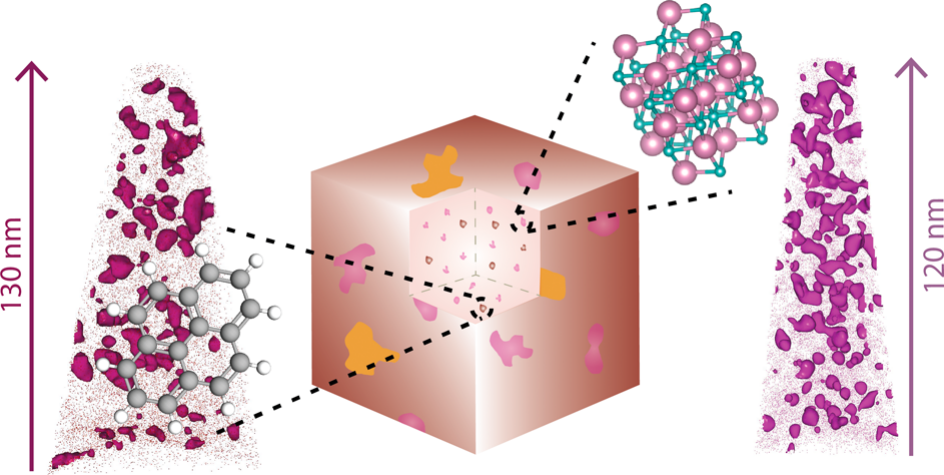

The conversion of methanol to valuable hydrocarbon molecules is of great commercial interest, as the process serves as a sustainable alternative for the production of, for instance, the base chemicals for plastics. The reaction is catalyzed by zeolite materials. By the introduction of magnesium as a cationic metal, the properties of the zeolite, and thereby the catalytic performance, are changed. With atom probe tomography (APT), nanoscale relations within zeolite materials can be revealed: i.e., crucial information for a fundamental mechanistic understanding. We show that magnesium forms clusters within the cages of zeolite SSZ-13, while the framework elements are homogeneously distributed. These clusters of just a few nanometers were analyzed and visualized in 3-D. Magnesium atoms seem to initially be directed to the aluminum sites, after which they aggregate and fill one or two cages in the zeolite SSZ-13 structure. The presence of magnesium in zeolite SSZ-13 increases the lifetime as well as the propylene selectivity. By using *operando* UV–vis spectroscopy and X-ray diffraction techniques, we are able to show that these findings are related to the suppression of aromatic intermediate products, while maintaining the formation of polyaromatic compounds. Further nanoscale analysis of the spent catalysts showed indications of magnesium redistribution after catalysis. Unlike zeolite H-SSZ-13, for which only a homogeneous distribution of carbon was found, carbon can be either homogeneously or heterogeneously distributed within zeolite Mg-SSZ-13 crystals as the magnesium decreases the coking rate. Carbon clusters were isolated, visualized, and analyzed and were assumed to be polyaromatic compounds. Small one-cage-filling polyaromatic compounds were identified; furthermore, large-cage-crossing aromatic molecules were found by isolating large coke clusters, demonstrating the unique coking mechanism in zeolite SSZ-13. Short-length-scale evidence for the formation of polyaromatic compounds at acid sites is discovered, as clear nanoscale relations between aluminum and carbon atoms exist.

## Introduction

The methanol-to-hydrocarbons (MTH) process is emerging as a renewable route for the production of plastics and fuels, as it can reduce the depletion of crude oil sources when methanol is produced from biomass, or waste or directly from CO_2_.^1−5^ The MTH reaction is catalyzed by zeolite materials, which, due to their unique porous network, can act as molecular sieves allowing only certain intermediates to form and products to diffuse out. In this way, small-pore zeolites (e.g., CHA) are highly selective toward lower olefins, such as ethylene and propylene, resulting in a process known as the methanol-to-olefins (MTO) process.^6−8^ The building blocks of zeolites are silicon, aluminum, and oxygen atoms. The different charges of the Si^4+^ and Al^3+^ framework elements result in an overall charge imbalance (negative) of the zeolite framework, which has to be compensated by a countercation.^9,10^ The nature of this countercation in zeolite materials has a great influence on the acidic properties, which can act as an active reaction center and thereby alter the catalytic performance.^7^ The performance of zeolite catalysts in the MTH reaction can also be altered by changing the strength and number of the active acid sites by, for example, exchanging the commonly present H^+^ countercation by positively charged metal ions.^7,11,12^ Previous studies of Yarulina et al. showed that the introduction of the alkaline-earth metal Ca^2+^ into zeolite ZSM-5 resulted in a 10-fold reduction of Brønsted acidity, which in turn suppressed the aromatization cycle, leading to a higher selectivity toward propylene as well as a longer catalyst lifetime.^13−18^ Similar observations were made by Goetze et al., who studied the evolution of aromatic reaction intermediates during the MTH reaction over Mg^2+^-modified ZSM-5 zeolites with *operando* UV–vis spectroscopy.^19^

These observations illustrate that the catalytic performance of zeolites in the MTH reaction depends on the structure and elemental composition of the zeolite framework. As was mentioned, these factors have already been quite extensively studied at the bulk scale, but mainly for the zeolite ZSM-5 (MFI).^7,13,14,19−21^ However, much is still unknown about the influence of the introduction of metal ions, such as Ca^2+^ and Mg^2+^, on the zeolite activation, reaction, and deactivation because zeolites are notoriously hard to study at the nanoscale and deriving nanoscale relationships in these materials largely remains elusive.^7,22−24^ Zeolites are unstable under electron beams typically used in high-resolution transmission electron microscopy (HR-TEM), and the framework elements Si and Al offer no significant *Z*-contrast difference needed for X-ray absorption spectroscopy (XAS) and X-ray diffraction (XRD) techniques.^25−27^ As a result, the understanding of nanoscale relations between framework elements, exchanged metals, and active/deactivating coke species is still limited, while this is crucial information to reveal e.g. MTH reaction and deactivation mechanisms.

Atom probe tomography (APT) is a key tool in this investigation of zeolite-based materials. It can provide information on the distribution and relations between components of zeolites and coke residues and can thereby be compared to other zeolite characterization methods such as nuclear magnetic resonance (NMR), X-ray (absorption) techniques, and time-of-flight secondary ion mass spectrometry (ToF-SIMS). However, these other techniques are either limited to bulk-scale analysis or do not have the nanoscale spatial resolution as APT is providing. As mentioned, the use of other high-resolution imaging techniques based on electron microscopy are limited due to the instability of zeolites under high-intensity electron beams and due to the limiting *Z*-contrast in studies. As in APT analysis the identification of the elements is based on time-of-flight mass spectrometry (ToF-MS), atomic-specific 3-D ion maps can be reconstructed with sub-nanometer resolution and this technique is thereby uniquely positioned among other zeolite characterization methods.^25^ In this study, we have used APT, coupled with *in situ* and *operando* techniques, i.e., XRD and UV–vis spectroscopy, to develop structure–performance relationships in the MTH process over pristine and magnesium-modified zeolite SSZ-13 (CHA). As the chabazite structure is especially known to be highly selective toward lower olefins, and the introduction of magnesium to the medium-pore zeolite ZSM-5 has already been shown to enhance the selectivity toward propylene, the effect of magnesium on the reaction intermediates and products using zeolite SSZ-13 as a showcase material is of great interest.^28−31^ Therefore, by using *operando* spectroscopy, the differences in formed intermediates, structural changes, and product distributions were analyzed. With APT, the nanoscale relations among the zeolite framework elements, countercations, and carbon molecules formed during the reaction were determined by a statistical data analysis. In this way, the distribution of the introduced magnesium was studied on the nanoscale and found to be highly heterogeneous, allowing for the isolation of magnesium clusters, which could be correlated to carbon and aluminum. Also, the influence of magnesium on coking behavior was revealed, and it was found that, on the nanoscale, the carbon compounds are more heterogeneously distributed in the Mg-exchanged samples compared to non-magnesium samples, also allowing for the analysis of carbon clusters. With this approach, we attempt to contribute to the further understanding of the complex reaction mechanisms in zeolite materials.

## Experimental Section

### Catalyst Preparation

Zeolite H-SSZ-13 was synthesized according to the procedure by Oord et al.^32^ Before the impregnation step the zeolite powder was dried at 120 °C. Afterward, the zeolite powder was impregnated by incipient wetness impregnation with an aqueous solution of Mg(NO_3_)_2_·6H_2_O in deionized water, with gentle mixing, aiming for 1 wt % Mg. The impregnated material was dried overnight at 120 °C in a static conveying followed by calcination at 550 °C (with a drying step at 150 °C and a temperature ramp of 1 °C/min) under air for 5 h.

### Bulk Catalyst Characterization

Temperature-programmed desorption with ammonia (NH_3_-TPD) was performed using a Micrometrics Autochem 2910 apparatus. The instrument is equipped with a thermal conductivity detector (TCD) to determine the amount of ammonia. A 100 mg portion of the sample was placed in a quartz tube with a quartz glass grid. First, the sample was dried at 550 °C for 30 min (heating ramp of 10 °C/min) under a He flow. Second, the sample was cooled to 100 °C and 20 pulses of 25.13 cm^3^/min NH_3_ were applied to make sure all acid sites of the zeolites were saturated. The sample was outgassed for 2 h at 100 °C. Finally, with a heating ramp of 5 °C/min, the sample was heated until 550 °C, while the amount of desorbed NH_3_ was measured. The data were analyzed by fitting the TCD concentration curve with three Gaussian functions, which were integrated to determine the moles of desorbed NH_3_, with the use of Fytik software.^32,33^ X-ray diffraction (XRD) was performed with a Bruker D2 Phaser diffractometer, in Bragg–Brentano mode, equipped with a Lynxeye detector to determine the framework structure of the zeolite-based catalysts. The instrument makes use of a fixed slit and Co Kα_1_ radiation (λ = 1.79026 Å) and was operated at 30 kV and 10 mA. XRD measurements were taken with a scan speed of 0.5 s per step, in a 2θ range between 7 and 60°, with an increment of 0.025°, resulting in 2120 steps. Scanning electron microscopy (SEM) images were taken with a Phenom Scanning Desktop Electron Microscope equipped with an energy dispersive X-ray (EDX) detector in backscattering mode at 15 kV. Inductive coupled plasma optical emission spectroscopy (ICP-OES) measurements were performed to determine the composition of the zeolite material by a Li_2_B_4_O_7_ destruction using a PerkinElmer Avio 500 instrument.

### *Operando* UV–Vis Diffuse Reflectance Spectroscopy

A methanol (MeOH) flow over a quartz rectangular fixed-bed reactor was generated by bubbling He through a methanol saturator kept at a fixed temperature (19 °C, 14.5 vol % of MeOH). Catalysts were activated by calcination under a 10 mL/min O_2_ flow at 550 °C (5 °C/min). After this, the reactor was cooled to the catalytic test temperature of 450 °C with a 35 mL/min He flow. A weight hourly space velocity (WHSV) of 1.3 h^–1^ was obtained by using the following parameters: 60 mg of zeolite (212–425 μm), 14.5 ± 0.5% methanol saturation, and 6.5 mL/min He flow for 180 min. Online activity and selectivity measurements were performed with an Interscience Compact gas chromatograph (GC). UV–vis diffuse reflectance spectroscopy (DRS) spectra were collected using an AvaSpec2048L spectrometer connected to a high-temperature UV–vis probe, to obtain information about the intermediate products. Further details of the setup can be found in earlier publications of our group.^19,33−35^ Methanol conversion and product yield were calculated using the same methods as explained in ref (19).

### *Operando* X-ray Diffraction

*Operando* XRD was done with a Bruker D8 discover diffractometer instrument equipped with a Mo X-ray source (Mo Kα, λ = 0.709 Å). Experimental details of this setup can be found in earlier publications of our group.^36,37^ A glass capillary with a 1 mm thickness (thickness wall 0.02 mm) was filled with 10 mg of SSZ-13 (20 mm, 150–120 μm sieve fraction). A high-resolution XRD pattern was acquired before calcination for reference. The catalyst bed in the glass capillary was first heated to 500 °C (heating rate 5 °C/min) under a 5 mL/min O_2_ flow, inside an infrared furnace steered by a thermocouple inserted in the capillary up to the catalyst bed. The temperature and flow were stabilized for 60 min. Throughout the whole calcination step, XRD patterns were collected every 10 min. The catalyst bed was cooled to 450 °C, and the flow was switched to He. Before starting the MTH reaction, a high-resolution XRD pattern was collected at 450 °C. MeOH was flowed on the catalyst by flushing 1.5 mL/min of He through a saturator in a water bath kept at 10 °C. A WHSV of 0.7 h^–1^ was obtained by the following parameters: 10 mg (125–212 μm), 5.8%, and 1.5 mL/min He flow. An online Interscience TraceGC 1300 GC instrument was used to follow the MTH activity and selectivity. After deactivation, the methanol input was closed and an XRD pattern at 450 °C was recorded with and without He flow. Finally, the reactor bed was cooled and an XRD pattern was recorded at RT with and without He flow. In order to quantify the structural changes of the zeolite materials under study, Rietveld refinement was applied on the recorded XRD patterns. For this purpose, the Topas academic V5 software was used. Rietveld refinement is based on a formula in which the material structure, X-ray source emission profile, and instrument details determine the overall XRD pattern. The emission profile can be measured and is determined by the X-ray source, while the instrumental details typically depend on the instrument configurations and geometries. The structure information was obtained from the PDF database. As after and during the MTH process carbon atoms are also present in the cages and are thereby nonrandomly distributed, carbon atoms were placed in the cages in this structure file. The background of the XRD patterns was fitted with a three-term Chebyshev polynomial. The sample displacement function was used to correct for sample height differences introduced by the heating of the capillary. The crystallite strain was described as a Lorentzian function.

### Methanol-to-Hydrocarbons Coking Experiments

The Mg-SSZ-13 catalyst was placed in a Linkam cell (HFS600) and pretreated at 150 °C for 30 min (5 °C/min heating rate) under a N_2_ flow (5 mL/min), followed by a heating step to 450 °C under a N_2_ flow (5 mL/min). The MTH reaction was performed at 450 °C with a N_2_ flow of 5 mL/min through a ^13^C-labeled methanol (99 atom %, Sigma-Aldrich) saturator. The MTH reaction was performed for 1, 15, 30, and 60 min to induce different degrees of coking.

### Atom Probe Tomography

Atom probe tomography (APT) measurements were performed at the Oak Ridge National Laboratory (ORNL). The APT needles used for the APT analysis were prepared as described by Schmidt et al. using focused ion beam-scanning electron microscopy (FIB-SEM).^25^ Additionally, the assessment and precautions for possible Ga damage was described in the same publication. A Si microtip, purchased from CAMECA, was used as a substrate for APT needle preparation using the FIB-milling technique with a Thermo Fisher Nova 200 dual-beam SEM/FIB instrument. We attempted to prepare APT needles from similar positions (50 ± 50 nm below the surface) in the different crystals. Different needles were reconstructed from different crystals and of different samples, resulting in different APT data sets. The fabrication of SSZ-13 needles has been explained in previous work.^25,38^ The APT measurements were performed using a LEAP 4000XHR local electrode atom probe in laser mode using a 200 pJ laser pulse energy, a 40 K base temperature, and a 0.5–2.0 detection rate. All APT analyses were performed with CAMECA’s integrated visualization and analysis software (IVAS).

The theory about the different data analysis techniques has been extensively explained in different previous publications.^23,25,39^ However, the most important theory will be discussed in this section for reasons of clarity. With a nearest-neighbor distribution (NND) analysis, the distribution of atoms with respect to the distance from each other is analyzed. Simulated random nearest-neighbor distance distributions were compared to the measured distance between nearest neighbors of similar nature. Clustering of elements will result in a multipeak Gaussian distribution, with one peak showing a maximum at shorter distances than the simulated random distributions.^25,40−42,25,43^ The difference between the measured and theoretical distributions can be quantified using a χ^2^ statistical test normalized by the sample size (Pearson coefficient, μ). In this article, we follow a similar approach previously presented in ref (38). A radial distribution function (RDF) analysis was used to discover heterogeneous distributions, i.e. short-length-scale affinities between elements, by comparing local concentrations to bulk concentrations.^25,44−46^ The maximum separation method was used to isolate elemental clusters from the bulk. The size and the composition of the clusters were determined in this way and were compared to calculated cluster sizes of spherical MgO particles with similar numbers of Mg atoms using Avogadro software.^25^

## Results and Discussion

In the first part of this work, we investigated the state of magnesium and the related properties of the Mg-SSZ-13 material at the bulk scale by applying conventional zeolite characterization methods: i.e., scanning electron microscopy-energy dispersive X-ray (SEM-EDX) analysis, NH_3_ temperature-programmed desorption (NH_3_-TPD), X-ray diffraction (XRD), and N_2_-physisorption. In a second part, the magnesium properties, elemental relations between the framework elements, and atomic distributions are described at the atom scale using atom probe tomography (APT). In a third part, the effect of magnesium on the catalytic properties and the changing catalyst properties are explored using *operando* spectroscopic and diffraction techniques. Lastly, the influence of the introduction of magnesium in zeolite SSZ-13 on the nanoscale coking properties are elucidated by making use of APT.

### Effect of Magnesium on the Physicochemical Properties of Zeolite Mg-SSZ-13

**Figure 1:**
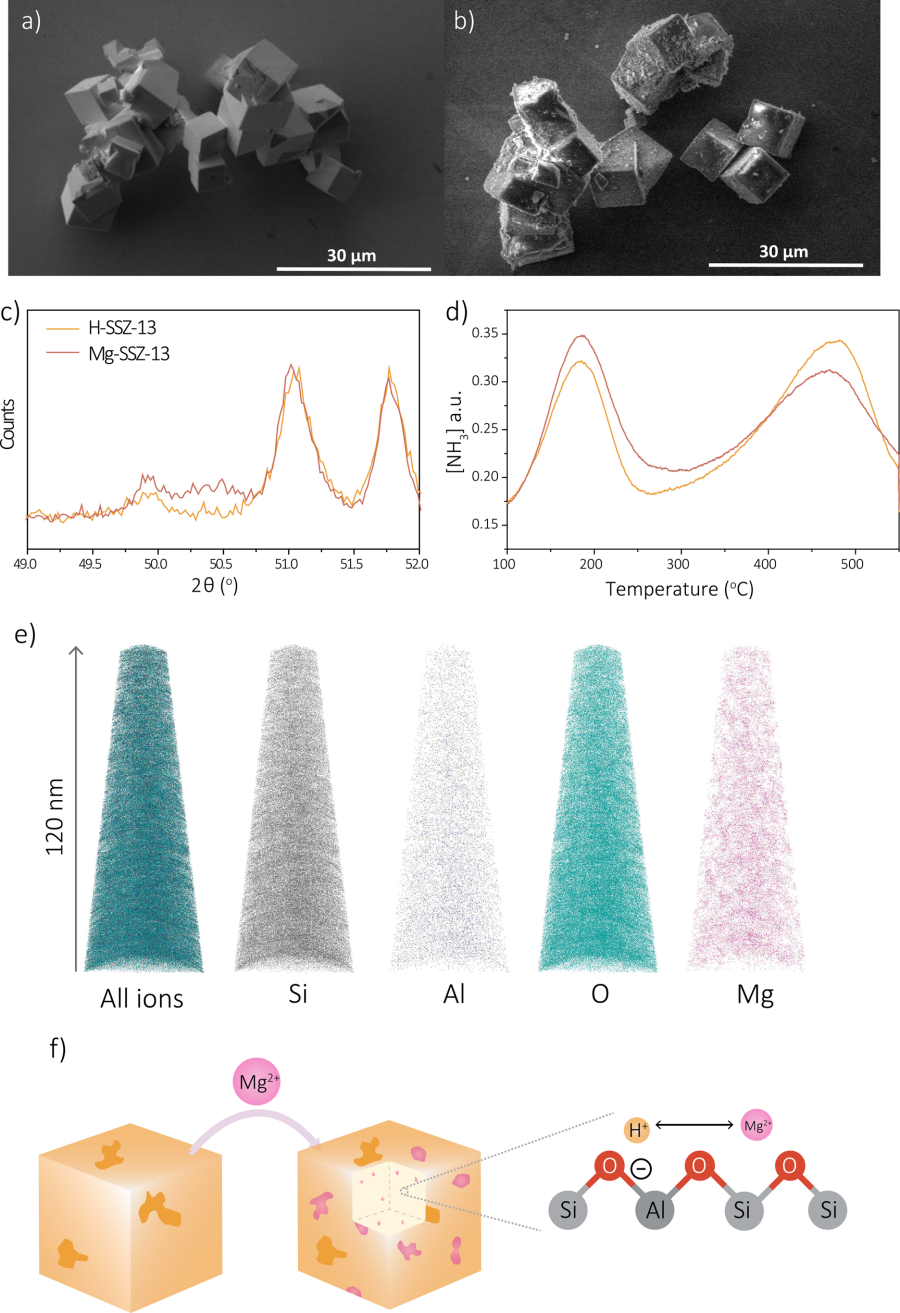
Analysis methods to determine the effect of magnesium on the catalyst properties. Scanning electron microscopy (SEM) images of (a) zeolite H-SSZ-13 and (b) zeolite Mg-SSZ-13. (c) X-ray diffraction (XRD) patterns of zeolite H-SSZ-13 and Mg-SSZ-13 enlarged in the MgO region. (d) NH_3_ temperature-programmed desorption (TPD). (e) Reconstructed atom probe tomography (APT) data sets of zeolite Mg-SSZ-13. (f) Schematic representation of the results obtained.

The bulk composition of the material is determined with inductively coupled plasma-optical emission spectroscopy (ICP-OES). The magnesium content is found to be 1.6 wt %, and the Si/Al ratio before and after showed limited dealumination of the zeolite material. The micropore volume decreased by a small extent, indicating that N_2_ molecules can still diffuse through the pore system of the zeolite crystal properly and that the magnesium does not significantly block the micropores. These results are summarized in Table S1. With SEM-EDX the bulk distribution and magnesium content can be determined. H-SSZ-13 zeolite crystals are cubes of about 5–10 μm in size and their smooth surface is sometimes interrupted by small intergrowth crystals (Figure 1a). After introducing magnesium, some roughness is observed (SEM) and magnesium is detected with SEM-EDX. This means that the magnesium is at least partially located on the outer surface of the zeolite crystals, as depicted in Figure 1b, although it is unclear what the exact penetration depth and hence surface sensitivity are of this analytical method. The zeolite crystal structure and crystallinity have not been altered by the introduction of magnesium, as observed from the XRD diffraction patterns in Figure 1c. However, a small MgO oxide peak at 50.5° 2θ could be related to the exterior MgO species observed with SEM. The aim of introducing magnesium was to exchange the H^+^ cations by Mg^2+^ cations, resulting in a change in the zeolite’s acidity. The introduction of magnesium did indeed substantially change the acidic properties, which is a proof of magnesium ions located inside the cages of the zeolite. However, Figure 1d clearly shows two desorption NH_3_ peaks indicating weak (Lewis) and strong (Brønsted) acid sites, depending on temperature, and a clear decrease in Brønsted acidity by the introduction of magnesium is observed.^47,48^ However, most Brønsted acid sites remain after the procedure, meaning that not all acid sites are affected, that different Brønsted acid sites are formed, or both.^19^ To quantify the changes in zeolite acidity, the NH_3_-TPD curves were fitted with three Gaussian curves.^32^ The measured curves, the three Gaussian curves, the fitted curves, and further explanation can be found in Section 1.3 and Figure S2 in the Supporting Information. The semiquantitative results can be found in in Table S2. Besides the clear decrease of the high-temperature peak, the low-temperature peak is increased by the introduction of magnesium, possibly due to the introduction of Lewis acidity. Comparing the differences between the micropore volume and the changes in acidity, the observed reduction of Brønsted acidity cannot be assigned to the reduction in micropore volume area, as this difference is too low to have a large effect. Additionally, for similar reasons, the effect of the decrease in Brønsted acidity cannot be related to the dilution of the sample with MgO, as the introduced weight loading is much lower (1.6 wt %) than the changes in acidic nature of the zeolite would indicate. Figure 1f schematically illustrates all of these results, including the presence of magnesium on the outer surface of the zeolite crystals and reduced Brønsted acidity resulting from the possible exchange of H^+^ by Mg^2+^ atoms. The effect of magnesium on the zeolite SSZ-13 properties is found to be similar to its influence on the zeolite ZSM-5, allowing for further comparison.^14,19^

To obtain insights into the effect of magnesium, nanoscale information is crucial. APT experiments were performed to analyze the atom distributions on sub-nanometer scale in different reconstructed needles, which were fabricated from Mg-SSZ-13 crystals. Three needles of calcined zeolite Mg-SSZ-13 were successfully reconstructed. One of these reconstructed needles is depicted in Figure 1e, while the other two are presented in Figure S3. The first data set did not contain magnesium and only the zeolite framework elements were found. The second third data sets both contained magnesium (2.4% and 0.08%, respectively). All compositions from the data sets obtained of the pristine Mg-SSZ-13 samples can be found in Table S3. This means that the magnesium is heterogeneously distributed on the bulk/macro scale, also related to the observed heterogeneity on the surface of microcrystals with SEM-EDX. Additionally, as the needles were prepared from the interior of the zeolite crystals, this proves that the magnesium is indeed also located inside the pore architecture of the zeolite.

**Figure 2:**
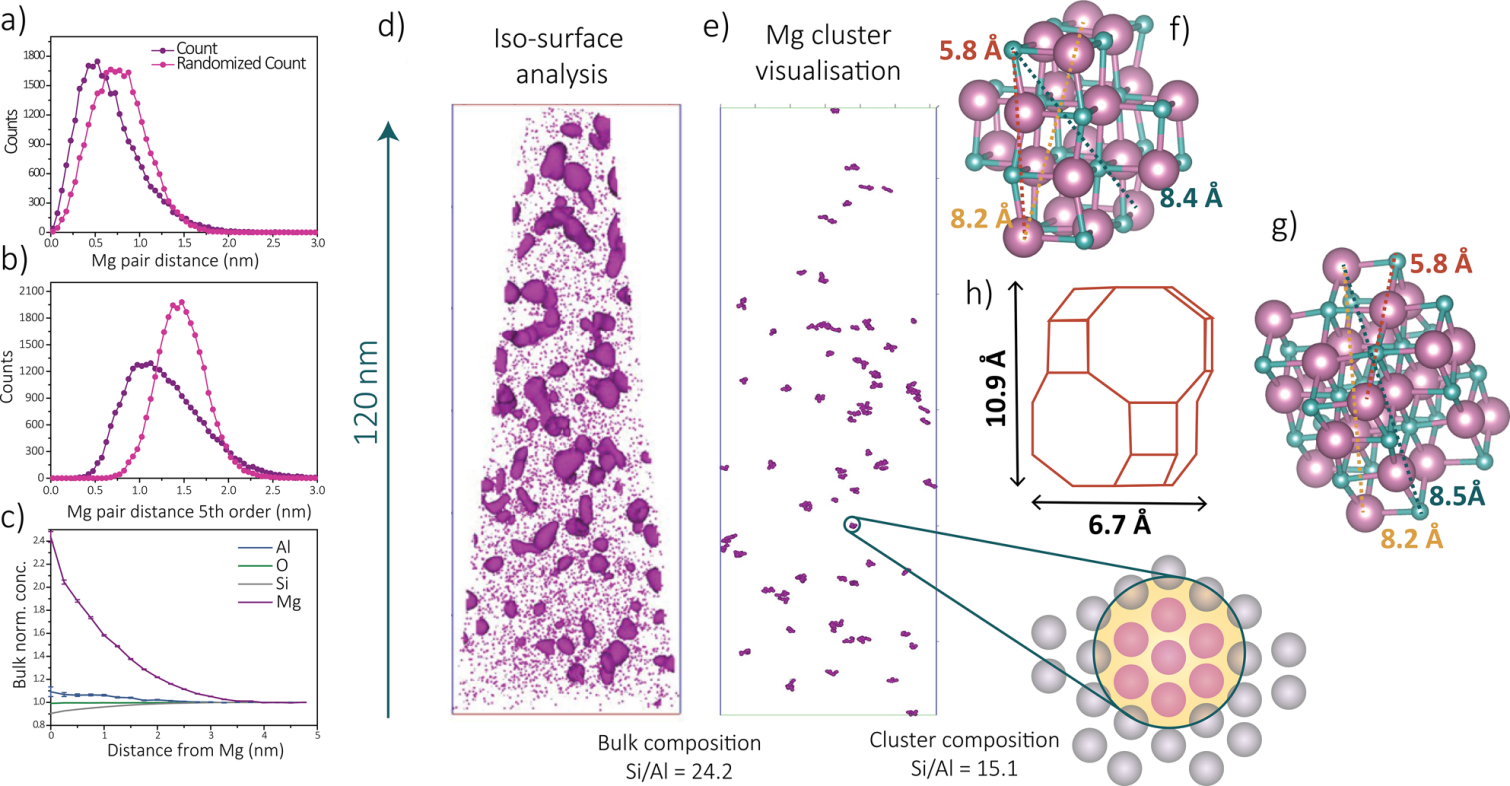
(a) First- and (b) fifth-order nearest-neighbor distribution (NND) analyses and (c) a Mg radial distribution analysis. (d) Reconstructed needle with isosurfaces drawn with a concentration of 2× the bulk value (7.2%) to show the magnesium-rich areas. (e) Cluster visualization in the data set after applying the maximum separation method for cluster analysis. Enlarged schematic representation of the clusters visualized with the maximum separation method with purple representing magnesium atoms and gray representing other (framework) elements. The bulk composition is the composition of the APT data sets, while the cluster composition is the composition of the isolated clusters. A possible MgO cluster forms with dimensions of (f) 20 and (g) 24 magnesium atoms. (h) The zeolite cage dimension in which these clusters are supposed to fit.

Moreover, the magnesium distribution in the reconstructed needles presented in Figure 1e already seems quite heterogeneous on the nanoscale. Statistical methods can be used to prove this heterogeneity: e.g., a nearest-neighbor distribution (NND) analysis and radial distribution function (RDF) analysis. All NND and RDF of the pristine zeolite Mg-SSZ-13 samples, measured with APT, can be found in Sections 2.1.3 and 2.1.4 of the Supporting Information. The second data set contains the largest amount of magnesium, allowing for a higher accuracy data analysis, and we will therefore focus on this sample from now on. However, in both Mg-containing APT data sets, prepared from the calcined Mg-SSZ-13 sample, the measured nearest-neighbor distribution deviates from the random distribution, which indicates a heterogeneous distribution of Mg. In Figure 2a, the first-order NND of zeolite Mg-SSZ-13 is depicted showing a deviation from a random distribution, meaning that the magnesium is indeed heterogeneously distributed on the nanoscale in zeolite SSZ-13. This heterogeneity in the magnesium distribution becomes even more noticeable on examination of the fifth-order NND as statistical fluctuations are reduced (Figure 2b). Similarly, from an NND analysis, the zeolite framework elements are found to be homogeneously distributed. Furthermore, as suggested from the bulk normalized concentrations in the RDF analysis (Figure 2c), a short-length-scale affinity between magnesium atoms exists (Mg–Mg short-length-scale affinity), as the magnesium concentrations are significantly larger at shorter length scales around other magnesium atoms. Furthermore, a small-length-scale affinity between aluminum atoms and magnesium atoms is observed in Figure 2c. This is intuitive because of the charge mismatch of the framework elements of a zeolite, and therefore, the magnesium is acting initially as the counterion in the framework: i.e., magnesium is initially attracted to the Al sites, after which it is obviously is clustering, which could be due to the calcination. With an isoconcentration (isosurface) surface analysis (Figure 2d), magnesium-rich and -poor areas can be identified within the volume of the reconstructed needle.

All of the isosurface analyses of magnesium are shown in Figure S10. Clear, statistically significant magnesium-rich areas were identified, also strengthening the evidence for a heterogeneous magnesium distribution inside the zeolite pores. As the distribution of magnesium is obviously heterogeneous in the zeolite SSZ-13 framework on the nanoscale, a cluster analysis can be performed. The maximum separation method was used to extract Mg clusters from the data set (*D*_max_ = 0.46 nm, *N*_min_ = 7 ions, and order 1). In this way, the clusters of just a few nanometers can be spatially visualized in 3-D (Figure 2e) and the composition and size of these clusters can be analyzed. A total of 77 clusters were found in the data set. Cluster analysis counted clusters of at least 7 magnesium atoms, oxygen, and other framework elements. As the detector efficiency is 33%, the number of counted atoms has to be multiplied by ∼3 to get a rough estimate of the actual number of magnesium atoms in one cluster. It is worth noting that these results do not exclude the existence of Mg^2+^ ions anchored to the zeolite framework. The clusters found with this technique contain ∼21–30 magnesium ions per cluster. To be able to make an approximation of how large these magnesium oxide clusters would be, spherical clusters of MgO were created with 20 and 24 magnesium atoms (Figure 2f,g). The spherical clusters of these composition are compared to the cage dimensions (Figure 2h), and it is evident that these clusters occupy one or two connecting cages in the zeolite. It is worth noting here that there is no evidence that the magnesium clusters are spherical. A nonuniform morphology of the magnesium clusters would increase the changes of cage-filling magnesium species. The APT maximum separation method strategy can also be used to calculate the size of the clusters: the average volume of the clusters was found to be 483 Å^3^, the median volume was 333 Å^3^, and the mode volume was 167 Å^3^ (with most of the clusters being between 150 and 250 Å^3^). The size distribution of the magnesium clusters found with APT is shown in Figure S11. With the cage of a chabazite structure believed to be approximately 10.9 Å × 6.7 Å × 6.7 Å = 489 Å^3^ with an accessible pore volume of 413.04 Å^3^, this cluster analysis size determination would indicate that most clusters do fit inside a cage of the zeolite.^37,49^ This means that the theoretical calculations indicate clusters larger than the clusters measured with APT; however, they are of the same size order, and we could therefore hypothesize that the magnesium clusters are located inside one cage or intercross two cages, or two small magnesium clusters are located in two cages very close to each other. These results imply that Mg is both clustered and distributed homogeneously between the clusters. A point to consider when interpreting the APT data is that 2 out of 3 ions are lost (33% detector efficiency), making the detection of small clusters impossible. However, this does prove the formation of magnesium aggregates within the zeolite cages. The concentration of aluminum inside the clusters was found to be higher than in the bulk, which is, together with the small-length-scale affinity from the RDF, evidence that the clusters like to form next to aluminum, which relates to the bulk-scale results. The Brø̷nsted acidity of the zeolite decreased, indicating the initial direction of the magnesium atoms next to the aluminum framework atoms, after which, at least a part of the magnesium atoms form clusters inside the cages of the zeolite SSZ-13.

### Effect of Magnesium on the Catalytic Performances of Zeolite SSZ-13 in the Methanol-to-Hydrocarbons Reaction

**Figure 3:**
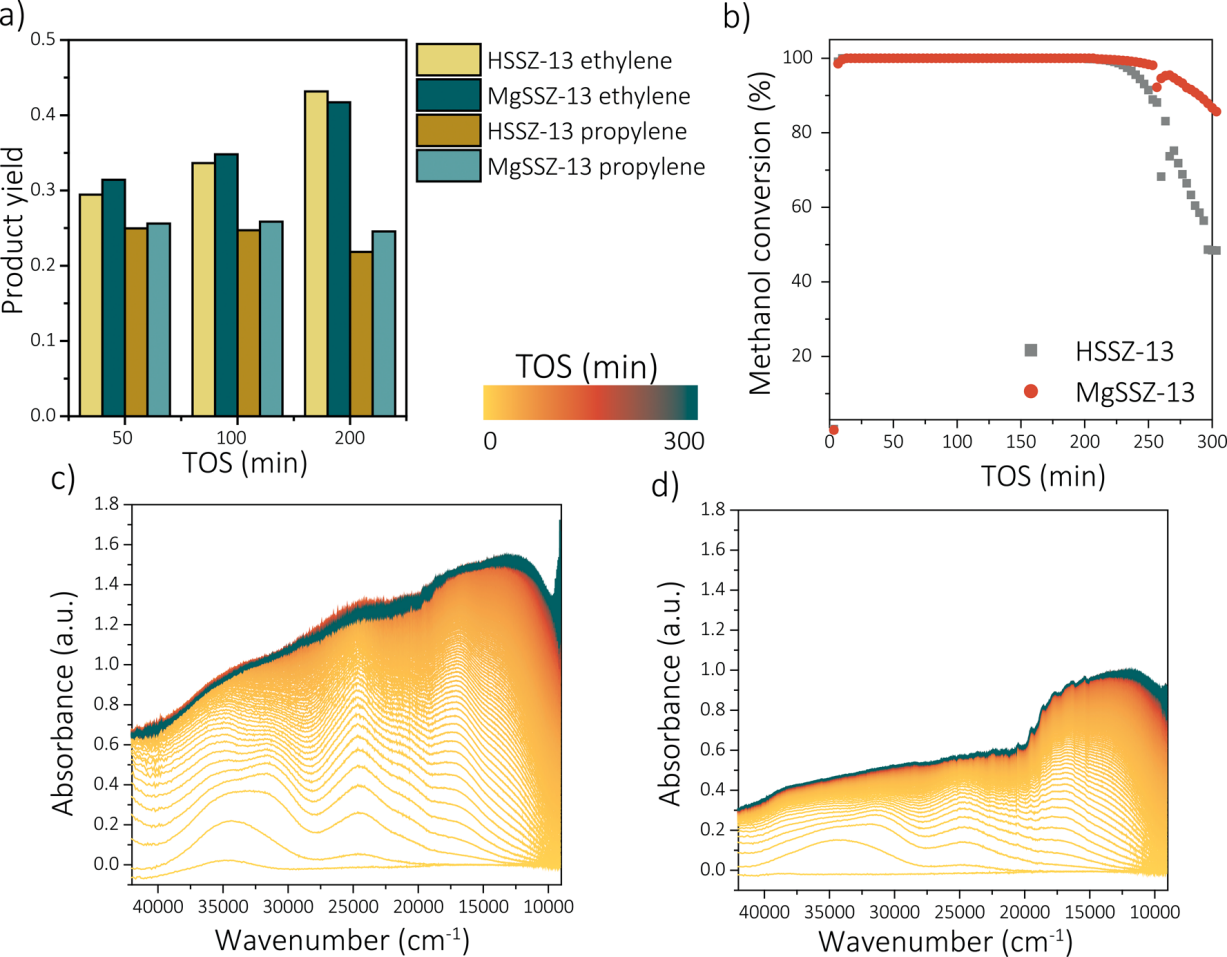
Catalytic performances of zeolite H-SSZ-13 and Mg-SSZ-13 studied with *operando* UV–vis diffuse reflectance spectroscopy (WHSV = 0.8 h^–1^, 450 °C): (a) ethylene and propylene selectivity; (b) methanol conversion over time. Operando UV–vis diffuse reflectance spectra of (c) zeolite H-SSZ-13 and (d) Mg-SSZ-13.

**Figure 4:**
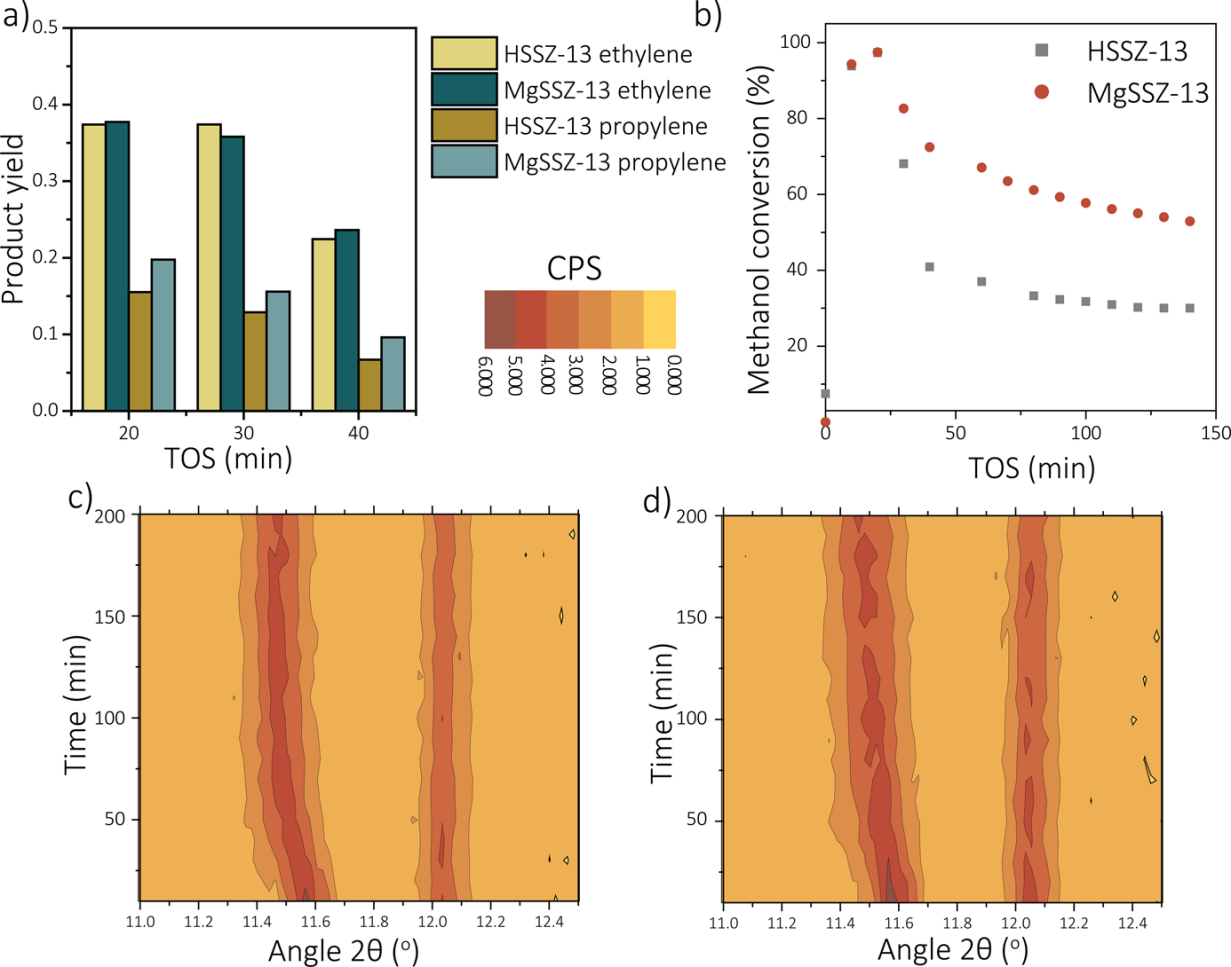
Catalytic performance of zeolite H-SSZ-13 and Mg-SSZ-13 studied with *operando* X-ray diffraction (XRD) (WHSV = 0.7 h^–1^, 450 °C): (a) ethylene and propylene selectivity; (b) methanol conversion over time. *Operando* XRD patterns of c) zeolite H-SSZ-13 and d) Mg-SSZ-13 in counts per second (CPS) between 11 and 12.5° 2θ to show the clear structural changes due to the methanol-to-hydrocarbons (MTH) reaction.

**Figure 5:**
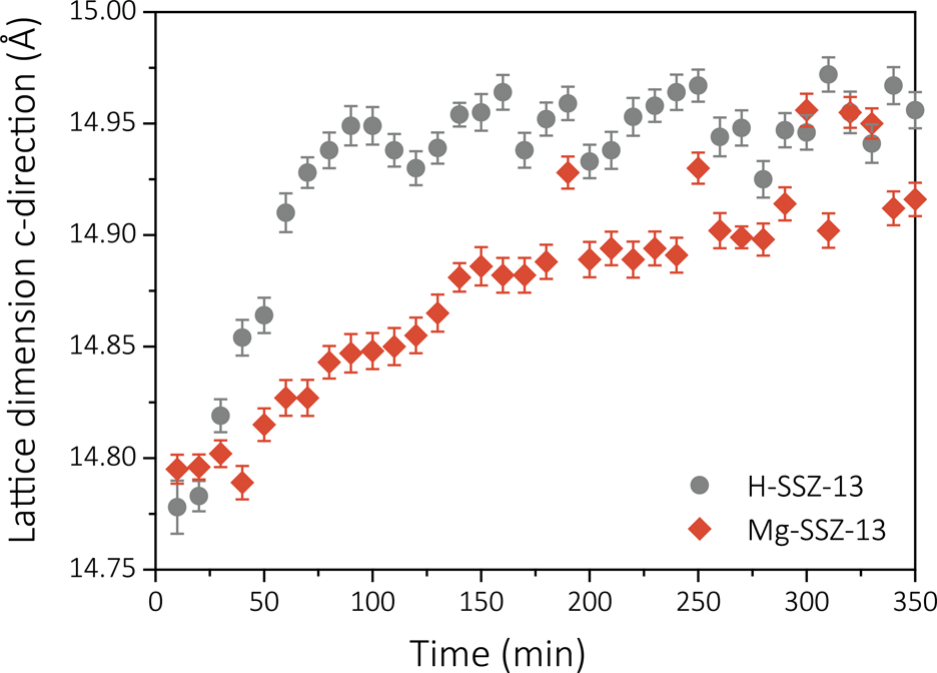
Dimensions of the lattice in the *c* direction determined with a Rietveld refinement analysis with increasing time on stream of zeolite H-SSZ-13 and Mg-SSZ-13 during the methanol-to-hydrocarbons (MTH) reaction (WHSV = 0.7 h^–1^, 450 °C).

The catalytic performances in the MTH reaction of the parent H-SSZ-13 as well as Mg-SSZ-13 were measured and correlated using *operando* UV–vis diffuse reflectance spectroscopy (DRS) and *operando* XRD. The catalytic performance of zeolite SSZ-13 is altered by the modification with magnesium, as can be observed in Figure 3, showing the *operando* UV–vis DRS results. Besides a slight enhancement of the catalytic lifetime, the rate at which the catalyst deactivates is also decreased (Figure 3b). This slower deactivation rate in the case of Mg-SSZ-13 (the slope of the conversion plot after the conversion stops to be 100%) is especially indicative of a difference in activating and deactivating behaviors. These changes are also translated in different product yields (selectivity to products). This is depicted in Figure 3a for three reaction times on stream, and the full product yield progression is shown in Figure S31. While the production of ethylene increased more rapidly for H-SSZ-13, the propylene product yield is higher for Mg-SSZ-13, especially after longer reaction times. Similarly to what was described previously for ZSM-5,^19^ the observed differences may be explained by *operando* UV–vis DRS. The fact that similar effects are observed for two different zeolite structures could indicate that the Mg is actually participating in the MTH reaction, rather than influencing shape selectivity. The *operando* UV–vis DRS results over time of the two catalysts under study are shown in Figure 3c,d. The modification of zeolite SSZ-13 with magnesium alters the reaction intermediates (i.e., hydrocarbon pool species, HCP) formed, which indicates a direct contribution of magnesium to the catalytic process. These results show that the magnesium also has an influence on the hydrocarbon pool species formed, which indicates the effect of magnesium on the catalytic process. More pronounced absorption bands at around 35000, 32000, and 25000 cm^–1^ are observed for zeolite H-SSZ-13, corresponding to neutral aromatics, charged monoenyl/cyclopentyl species and charged polyalkylated benzenes, respectively.^33^ In Section 3.1 in the Supporting Information, we also show the intensity evolutions at these wavelengths to stress the changes upon modification. However, as the UV–vis DRS data are the sum of different overlapping Gaussian-shaped absorption bands, these intensity evolutions cannot be used for quantification and will only be used to observe trends. The initial increase in absorption also decreases drastically with the introduction of magnesium (Figures S32 and S33). For instance, the contribution of the charged polyalkylated benzenes (25000 cm^–1^) seems to be less pronounced for zeolite Mg-SSZ-13, which are thought to be key intermediates for the formation of ethylene in the zeolite ZSM-5 dual-cycle mechanism.^7^ Therefore, a possible link between these aromatics and the formation of ethylene over zeolite SSZ-13 has been established. It seems evident that both catalysts are deactivated by the formation of polyaromatic compounds, which gives rise to adsorption bands between 17500 and 10000 cm^–1^.^33^ More specifically, the formation of these species in H-SSZ-13 and Mg-SSZ-13 seems rather similar when the relative absorbance intensities are compared with each other. This could indicate that the formation of polyaromatic compounds is not suppressed by magnesium, only the formation of the aromatic HCP species. The suppression of aromatic intermediate molecules by magnesium has also been described for ZSM-5, indicating a similar reaction mechanism. The actual reaction mechanisms in zeolite SSZ-13 are widely debated. This could mean that the dual-cycle principle, a MTH reaction mechanism established for ZSM-5, is also applicable for SSZ-13. The effect of different metal ions on the reaction intermediates formed will be the focus of future studies, in which the aromatic intermediates found with UV–vis DRS will be explained quantitatively. In this hypothesis, the introduction of magnesium ions suppresses the formation of aromatic intermediates within the cages of the zeolite SSZ-13 structure. The formation of fewer aromatic intermediates results in a longer lifetime, as coke precursor molecules are reduced, which results in a higher propylene selectivity and a slower deactivation rate. The same catalytic trends, in which the propylene selectivity is enhanced, the lifetime is increased, and the deactivation rate is reduced by the modification with magnesium, are observed with the *operando* XRD experiments (Figure 4a,b). The absolute differences in lifetime and product selectivity measured with *operando* UV–vis DRS and XRD techniques are due to the differences in reaction setups: e.g., reactor size differences and sieve fractions. Catalytic results and some contour maps of the measured diffractograms over time are shown in Figure 4 and in Figure S35. Structure–performance relations were obtained using *operando* XRD. The contour plots of the XRD patterns over time show that certain peaks (e.g., 11° 2θ) shift to lower angles with increasing time on stream due to the expansion of the zeolite lattice (Figure 4c,d). This phenomenon has been attributed to the formation of carbon molecules in the cages of the zeolite framework.^37^ The expansion of the lattice is found to only occur along the *c* direction, as only peaks corresponding to this direction are shifting.^37^ With a Rietveld refinement analysis, the expansion of the unit cell can be quantified with time on stream (Figure 5). All dimension parameters, *R*_wp_ factors, and peak positions can be found in the Supporting Information. The differences in coking behavior found with the *operando* UV–vis DRS experiments are correlated with differences in the development of the expansion of the unit cell lattice of the zeolites. The lattice expansion happens much more rapidly for zeolite H-SSZ-13 than for zeolite Mg-SSZ-13, which is in line with the faster formation of intermediate aromatic molecules in zeolite H-SSZ-13 and its faster deactivation rate. The slower increase of the lattice expansion with time on stream for zeolite Mg-SSZ-13 can be related to the formation and growth of aromatic molecules inside the cages of the zeolite even after deactivation, which happens at around 50 min time on stream for both zeolite catalysts. This phenomenon could explain the slower deactivation rate of zeolite Mg-SSZ-13 compared to zeolite H-SSZ-13 and strengthens the hypothesis of the reduced formation of aromatic (coke) molecules in the cages of the zeolite for zeolite Mg-SSZ-13. We therefore show evidence for the direct catalytic influence of the magnesium introduction on the MTH mechanism in the chabazite structure, which shows similarities to the introduction of magnesium to zeolite ZSM-5. Magnesium does indeed slow down the formation of aromatic intermediate molecules but does not prevent the formation of polyaromatic compounds.

### Nanoscale Coking Behavior

**Figure 6:**
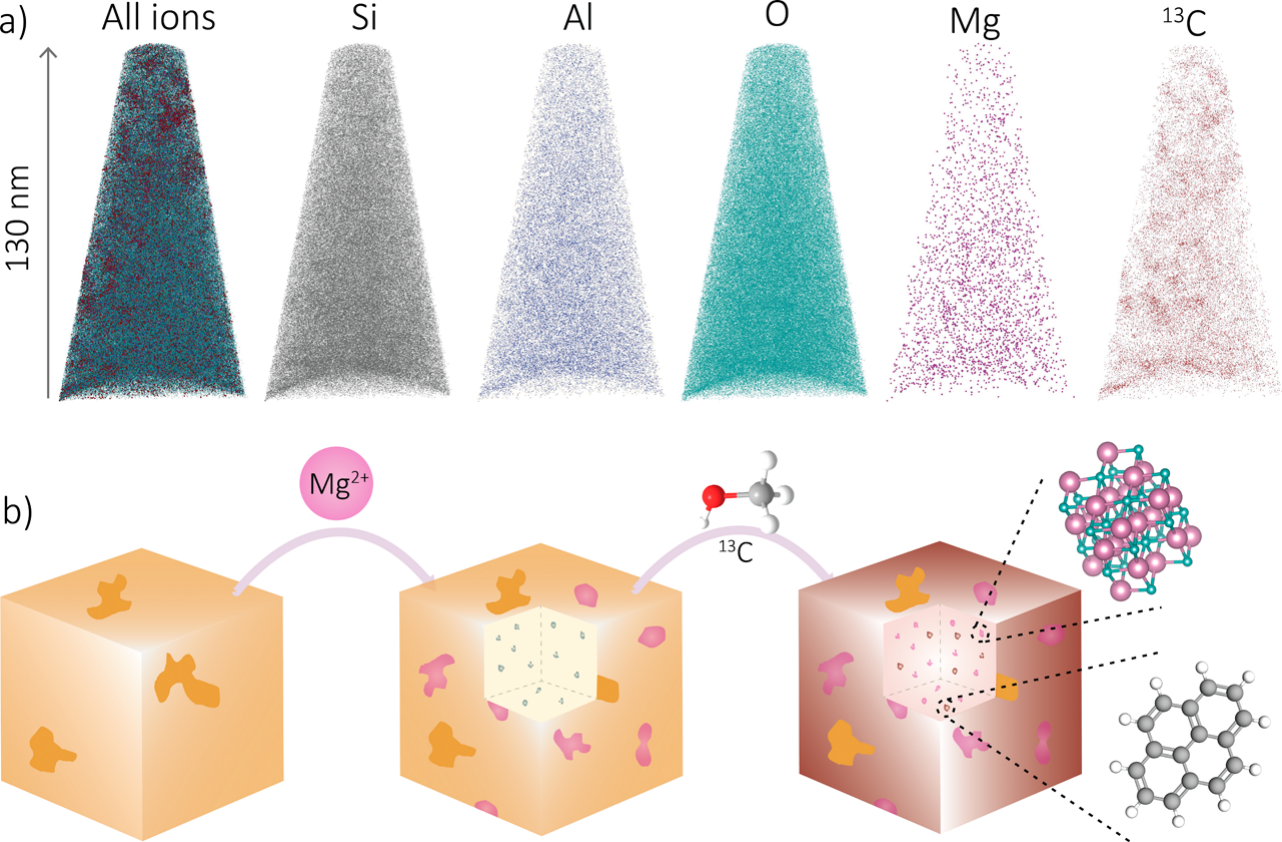
(a) Reconstructed atom probe tomography (APT) 3-D maps of the 1 min coked zeolite Mg-SSZ-13 samples, containing both magnesium and carbon. (b) Schematic representation of the results found, including the formation of magnesium and carbon clusters.

To obtain nanoscale information about the effect of magnesium on the zeolite properties and thereby the catalytic performance and coking behavior, the APT technique was applied to spent zeolite catalysts. Different spent catalyst materials were prepared, which were used in the MTH process using ^13^C-labeled methanol for different reaction times: i.e., 1, 15, 30, and 60 min time on stream (TOS). At least one needle per reaction time was successfully reconstructed, and the compositions of these APT data sets are depicted in Sections 2.2.1 and 2.2.2 in the Supporting Information. One example of a reconstructed needle containig both magnesium and carbon is presented in Figure 6a (1 min sample). However, this observation of both magnesium and carbon has not been found for the longer time on stream samples, as data sets containing a large amount of magnesium did not contain as much carbon and vice versa. This negative correlation between carbon and magnesium is thereby confirmed on the tens of nanometers scale (needle scale) and relates therefore to the bulk-scale observations in which fewer aromatic intermediates were formed. A schematic representation of the results found is shown in Figure 6b.

The degree of heterogeneity of the magnesium distribution can be compared between APT data sets using χ^2^ statistics applied to the NNDs. The Pearson coefficient (μ) is χ^2^ normalized to the sample size. A value of 0 indicates a completely random elemental distribution, while a value of 1 indicates a fully heterogeneous distribution of elements.^50^ The Pearson coefficients of the pristine zeolite Mg-SSZ-13 samples (0.47 and 0.90) are significantly larger than those for the 30 min coked zeolite Mg-SSZ-13 sample (0.22 and 0.18), indicating a more homogeneous distribution of magnesium in the spent catalyst. Overall, the distribution of Mg becomes more homogeneous over time. This indicates that magnesium is redistributing under the reaction conditions, such that it is dispersing homogeneously. To fully understand this phenomenon, a proper annealing study on Mg-SSZ-13 is necessary to fully understand how Mg redistributes with temperature. The Pearson coefficients of Si, Mg, and ^13^C for all data sets are shown in Table S8 in the Supporting Information and is shown over time in Figure S22.

As was explained before, magnesium suppresses the formation of aromatic molecules in the chabazite structure on the bulk scale. Similar results were found while analyzing the composition of the APT data sets, as a negative correlation between carbon and magnesium was discovered. All of the NNDs and RDFs are depicted in Sections 2.2.3 and 2.2.4 in the Supporting Information. The carbon distribution on the nanoscale is rather homogeneous for the longer time on stream samples (5–15 min coked samples), as the measured NND does not deviate significantly from the randomized NND. This is in line with the results found previously for zeolite H-SSZ-13, in which the carbon is homogeneously distributed as well, especially in comparison to ZSM-5, for which many carbon clusters could be identified. This effect also increased upon longer reaction times for zeolite H-SSZ-13 and is attributed to the high number of coked cages.^38^

**Figure 7:**
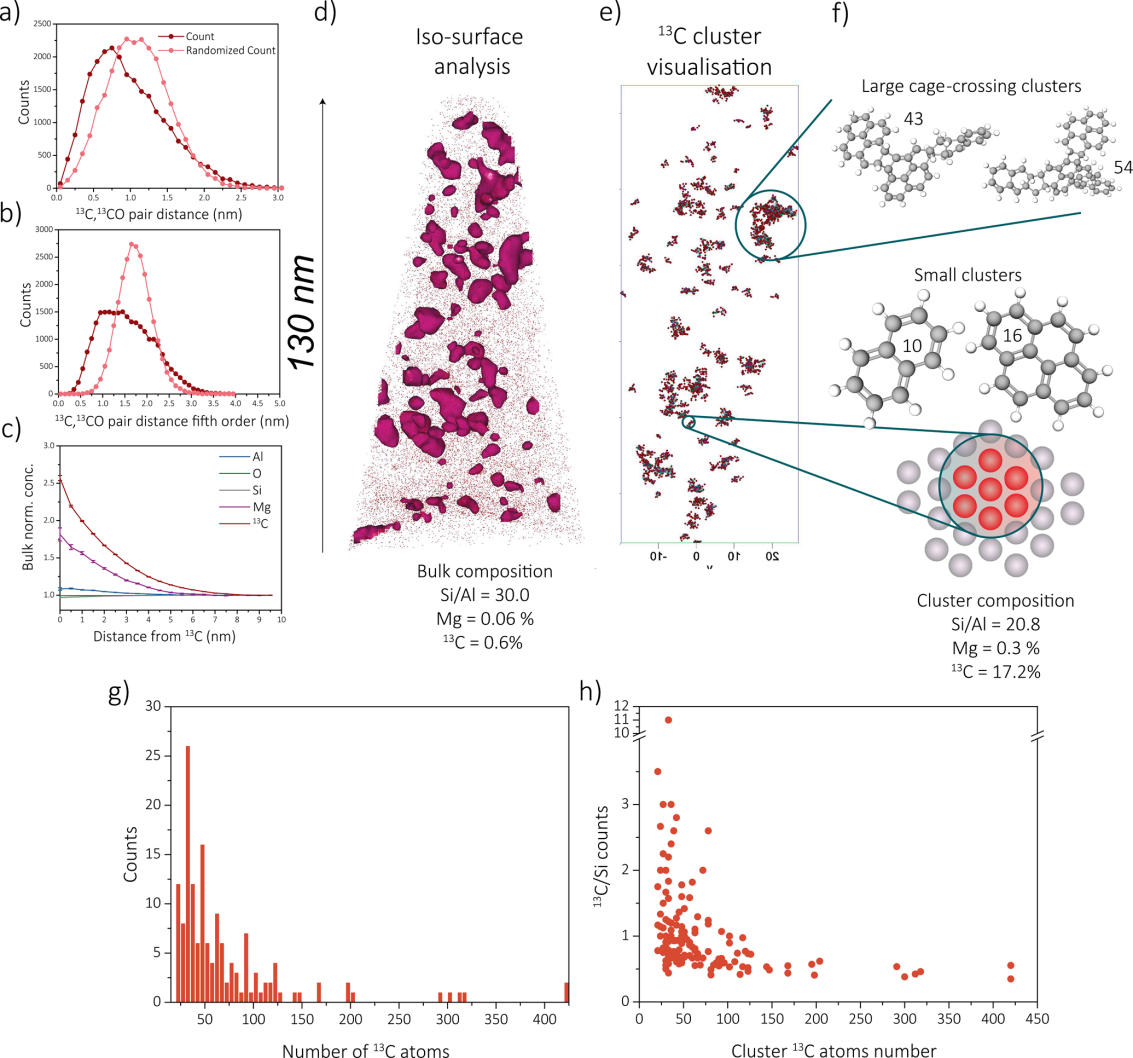
Results of the 1 min coked zeolite Mg-SSZ-13 samples on the distribution of carbon atoms: (a) first-order nearest-neighbor distribution (NND) analysis; (b) fifth-order NND analysis of ^13^C atoms and ^13^CO; (c) radial distribution function (RDF) analysis; (d) isosurface analysis; (e) cluster analysis; (f) some proposed hydrocarbon molecules compared with the size of the found clusters; (g) distribution of the number of carbon atoms per cluster; (h) comparison of the number of carbon atoms to the amount of framework elements.

It could be argued that the distribution of carbon in these longer time on stream samples is a bit more heterogeneous for Mg-SSZ-13 than for H-SSZ-13. However, a dissimilar trend is observed in a very short time on stream (1 min) zeolite Mg-SSZ-13 data set. This was the only 1 min data set containing both a significant amount of magnesium and carbon atoms. From Figure 6a, it is already evident that the carbon is not homogeneously distributed in this sample, as visual heterogeneities can be observed in the reconstructed ^13^C needle. For this specific data set, both the first- and fifth-order measured NNDs also deviate and shift to shorter distances compared to the random distribution, meaning that the carbon is heterogeneously distributed on the nanoscale (Figure 7a,b). Additionally, ^13^C–^13^C short-length-scale affinities were found with an RDF analysis (Figure 7c). An isosurface analysis also indicates clear carbon-rich areas in the data set (Figure 7d). Moreover, from Figure 7c in addition with the other RDF analyses displayed in Figures S23–S29, it can be concluded that a short-length-scale affinity between Mg–^13^C and Al–^13^C also exists. The affinity between ^13^C and Al atoms has been observed in previous work, but strong conclusions were not drawn. The fact that we see similar trends for all zeolites studied in the past (i.e., ZSM-5, SAPO-34, and SSZ-13) could mean that indeed the carbon clusters tend to form next to aluminum (Brønsted acid) sites.^25,39,38^ Mg clusters were isolated in this APT data set (1 min, data set 1). This, however, just as for the fresh calcined Mg-SSZ-13, does not mean that Mg is only present as clusters; and more dispersed and homogeneous Mg has also been found in the same APT data set. To obtain information on the role of the Mg clusters and the more homogeneously distributed Mg on the coking behavior, the RDFs of ^13^C and Mg in the APT data set with and without clusters present (more specific Mg-rich areas) were compared (Figure S29). After the exclusion of the Mg-rich areas, still a small nanoscale affinity between Mg atoms is observed, which would indicate the presence of smaller clusters below the resolution of the cluster isolation analysis. However, this affinity is extremely reduced, showing the high homogeneity of this Mg ions in the APT data set. It seems that the Mg–^13^C and ^13^C–Mg affinity is decreasing when the cluster analysis is excluded from the data set. This could indicate that the more dispersed Mg particles are contributing more to the suppression of the aromatic molecules than the clusters. The differences between the nanoscale coking behavior of the H-SSZ-13 and Mg-SSZ-13 materials, in which Mg-SSZ-13 does show a very clear heterogeneous distribution, can again, most probably, be attributed to the suppression of the aromatic species in Mg-SSZ-13, resulting in a reduced amount of coke in the sample, especially at the beginning of the reaction. The fast “overcoking” of zeolite SSZ-13 has been described before as the primary reason for the homogeneous distribtion of carbon.^38^ Since the carbon in this specific sample clearly is heterogeneously distributed, a cluster analysis can be performed, again using the maximum separation method (*D*_max_ = 0.92, *N*_min_ = 7, order = 5). Just as for the magnesium clusters, the size, location, and the composition can be determined. The cluster analysis results are shown in Figure 7e. The magnesium content inside the carbon clusters is higher than outside the clusters (in the bulk), which contradicts the previous results showing that magnesium suppresses the formation of hydrocarbon molecules on the bulk scale but does agree with the observed short-length-scale affinity between these elements. This could be due to the fact that both carbon and magnesium tend to deposit near aluminum (Figures 7c and 2c, and Figures S8, S9, and S23–S29), resulting in some overlap due to spatial blurring, and that the formation of large polyaromatics, which only can be detected with APT, is not necessarily suppressed by the presence of magnesium, something which was also found with *operando* UV–vis DRS. Additionally, we know from the bulk analysis that the formation of larger polyaromatic coke molecules is not suppressed by the modification with magnesium. When looking at the carbon cluster analysis, the number of carbon atoms detected start at 7 as *N*_min_ = 7, which means (when the detector efficiency has been considered) that clusters between 21 and 450 carbon atoms were detected, with 30–35 atoms as the mean cluster size value. This means that the smaller aromatic intermediate species cannot be detected, and the carbon clusters are assumed to be polyaromatic species, which also explains the correlation between magnesium and carbon. The distribution of the amount of carbon atoms in one cluster is depicted in Figure 7g. The decreasing ^13^C/Si ratio with a higher number of ^13^C atoms (shown in Figure 7h) indicates that the amount of silicon in the clusters with a large amount of carbon atoms is much higher. This means that these clusters are most likely crossing multiple cages and is an indication of the cage-crossing coking behavior of zeolite SSZ-13. Recent studies by Wang et al.^51^ also found that SAPO-34 (a zeotype catalyst with topology similar to that of zeolite SSZ-13) has a crossing-cage deactivation mechanism, in which polyaromatic molecules such as naphthalene are growing into larger aromatic hydrocarbon species that cross multiple cages. Some possible larger aromatic coke species, which are correlated to the number of carbon atoms found in specific clusters, are depicted in Figure 7f. On the other hand, smaller clusters, with for instance 21–35 carbon atoms, do generally not contain as much silicon, indicating that these clusters will occupy fewer or even just one or a few cages in contrast to the larger clusters. Further proof of the affinity of carbon clusters toward Al sites has been established by comparing the Si/Al ratio of the bulk to that of the cluster. It is observed that the Si/Al ratio in the carbon clusters (Si/Al = 20.8) is higher than that in the bulk (Si/Al = 30.0) resulting from the orientation of the clusters near Al sites. This is in agreement with the observed Al–^13^C short-length-scale affinity found with an RDF analysis.

Based on the above observations, we conclude that there is a correlation between the bulk coking and nanoscale coking behaviors, proving that the magnesium is interfering with the catalysis in such a way that the aromatic intermediate hydrocarbon pool species are suppressed, but not the formation of larger coke molecules, resulting in a longer catalyst lifetime and higher propylene selectivity. This suppressed coking rate leads to the possibility of isolating carbon clusters in zeolite SSZ-13, which was not possible without magnesium present.^38^ Additionally, we were able to isolate carbon clusters in zeolite SSZ-13 using APT, something which has not been done before. The additional proof of cage-crossing aromatic carbon molecules explains the fast deactivation mechanism of the chabazite structure.

Further research on this topic could focus on the other possible stoichiometry possibilities of magnesium (clusters or ions) in the chabazite structure and how they are initially exchanged at the (double or single) Al sites. Additionally, the synthesis method, e.g., direct inclusion of magnesium in the synthesis gel or ion exchange, could also have a large effect on the final magnesium distribution as well on the bulk-nanoscale coking behavior. The distribution of Mg (and other metals) could have a drastic effect on the final catalytic performance, which would require further comparison studies between samples with different metal distributions.^52^

## Conclusions

Zeolite SSZ-13 was successfully modified with magnesium via an impregnation preparation step. With atom probe tomography (APT), it was discovered that magnesium is heterogeneously distributed through the sample and tends to aggregate on the nanoscale, unlike the framework elements of the zeolite. With this technique, magnesium clusters with a size of just a few nanometers could be isolated, visualized, and analyzed. The cluster size was found to be similar to the size of one or two SSZ-13 zeolite cages. Additionally, a short-length-scale affinity and a higher concentration of aluminum inside the clusters prove a nanoscale relationship between aluminum and magnesium, indicating an initial affinity of magnesium to the Brønsted acid sites of the zeolite, after which it is clustering. The modification with magnesium leads to a longer catalyst lifetime in the methanol-to-hydrocarbons (MTH) reaction, a higher propylene selectivity, and a slower deactivation rate. By using *operando* UV–vis diffuse reflectance spectroscopy (DRS) and X-ray diffraction (XRD) these findings were attributed to the suppressed formation of aromatic intermediate species, similar to the case for zeolite ZSM-5, as the corresponding absorption bands were less pronounced and the lattice expansion happened more slowly. However, it was also found that magnesium does not prevent the formation of polyaromatic compounds, which deactivate the catalyst material. The exact mechanism behind the different catalytic performance is still unknown and requires further investigation by, for instance, changing the catalyst (e.g., different zeolites, weight loadings, and metal distribution) and reaction (e.g., temperature, WHSV) parameters. We would like to hypothesize that the introduced Mg is actively participating in the conversion of methanol, but further research would be necessary. A further nanoscale analysis showed that on a needle (tens of nanometers) scale, the magnesium and carbon are also anticorrelated, as APT data sets containing a large amount of carbon did not contain as much magnesium and vice versa. For the longer time on stream zeolite Mg-SSZ-13 samples, the carbon was homogeneously distributed, which is comparable to previous results for zeolite H-SSZ-13. However, in the shorter time on stream samples, the carbon was much more heterogeneously distributed, probably due to the suppression of the formation of aromatic molecules by magnesium. Isolated carbon clusters could be identified, spatially visualized, and analyzed. All of the carbon clusters are assumed to be polyaromatic molecules. Small carbon clusters, which potentially would fit in one or two SSZ-13 zeolite cages, were found, but also much larger clusters, proving that also large cage-crossing carbon clusters can exist. A short length scale between carbon and aluminum was found, reinforcing that the hydrocarbon formation indeed takes place on the acid sites of the zeolite. Moreover, although an anticorrelation between magnesium and coke has been found from the bulk to the tens of nanometers scale, a slight nanoscale affinity between magnesium and carbon was observed. This could be due to the fact that both magnesium and carbon are correlated to the aluminum sites. The nanoscale affinity between magnesium and aluminum supports the hypothesis that magnesium does not suppress the formation of deactivating polyaromatic compounds.

## Supporting Information Available

Additional discussions, figures, and tables as described in the text (ZIP)

## Abbreviations

APT: atom probe tomography
MTH: methanol-to-hydrocarbons
MTO: methanol-to-olefins
HR-TEM: high-resolution transmission electron microscopy
XAS: X-ray absorption spectroscopy
XRD: X-ray diffraction
TPD: temperature-programmed desorption
SEM: scanning electron microscopy
EDX: energy dispersive X-ray
WHSV: weight hourly space velocity
DRS: diffuse reflectance spectroscopy
GC: gas chromatograph
FIB-SEM: focused ion beam-scanning electron microscopy
IVAS: integrated visualization and analysis software
NND: nearest-neighbor distribution
RDF: radial distribution function.

## References

[ref1] Tian P.; Wei Y.; Ye M.; Liu Z. Methanol to Olefins (MTO): From Fundamentals to Commercialization. ACS Catal. 2015, 5, 1922–1938. 10.1021/acscatal.5b00007.

[ref2] Meunier N.; Chauvy R.; Mouhoubi S.; Thomas D.; De Weireld G. Alternative Production of Methanol from Industrial CO_2_. Renew. Energy 2020, 146, 1192–1203. 10.1016/j.renene.2019.07.010.

[ref3] Lange J. Methanol Synthesis: A Short Review of Technology Improvements. Catal. Today 2001, 64, 3–8. 10.1016/S0920-5861(00)00503-4.

[ref4] Ma J.; Sun N.; Zhang X.; Zhao N.; Xiao F.; Wei W.; Sun Y. A Short Review of Catalysis for CO_2_ Conversion. Catal. Today 2009, 148, 221–231. 10.1016/j.cattod.2009.08.015.

[ref5] Iaquaniello G.; Centi G.; Salladini A.; Palo E.; Perathoner S.; Spadaccini L. Bioresource Technology Waste-to-Methanol: Process and Economics Assessment. Bioresour. Technol. 2017, 243, 611–619. 10.1016/j.biortech.2017.06.172. 28709065

[ref6] Olsbye U.; Svelle S.; Bjrgen M.; Beato P.; Janssens T. V. W.; Joensen F.; Bordiga S.; Lillerud K. P. Conversion of Methanol to Hydrocarbons: How Zeolite Cavity and Pore Size Controls Product Selectivity. Angew. Chem., Int. Ed. 2012, 51, 5810–5831. 10.1002/anie.201103657. 22511469

[ref7] Yarulina I.; Chowdhury A. D.; Meirer F.; Weckhuysen B. M.; Gascon J. Recent Trends and Fundamental Insights in the Methanol-to-Hydrocarbons Process. Nat. Catal. 2018, 1, 398–411. 10.1038/s41929-018-0078-5.

[ref8] Olsbye U.; Svelle S.; Lillerud K. P.; Wei Z. H.; Chen Y. Y.; Li J. F.; Wang J. G.; Fan W. B. The Formation and Degradation of Active Species during Methanol Conversion over Protonated Zeotype Catalysts. Chem. Soc. Rev. 2015, 44, 7155–7176. 10.1039/c5cs00304k. 26185806

[ref9] Ashtekar S.; Chilukuri S. V. V.; Chakrabarty D. K. Small-Pore Molecular Sieves SAPO-34 and SAPO-44 with Chabazite Structure: A Study of Silicon Incorporation. J. Phys. Chem. 1994, 98, 4878–4883. 10.1021/j100069a018.

[ref10] Perea D. E.; Arslan I.; Liu J.; Ristanović Z.; Kovarik L.; Arey B. W.; Lercher J. A.; Bare S. R.; Weckhuysen B. M. Determining the Location and Nearest Neighbours of Aluminium in Zeolites with Atom Probe Tomography. Nat. Commun. 2015, 6, 7589 10.1038/ncomms8589. 26133270 PMC4506508

[ref11] Deimund M. A.; Harrison L.; Lunn J. D.; Liu Y.; Malek A.; Shayib R.; Davis M. E. Effect of Heteroatom Concentration in SSZ-13 on the Methanol-to-Olefins Reaction. ACS Catal. 2016, 6, 542–550. 10.1021/acscatal.5b01450.

[ref12] Valecillos J.; Epelde E.; Albo J.; Aguayo A. T.; Bilbao J.; Castaño P. Slowing down the Deactivation of H-ZSM-5 Zeolite Catalyst in the Methanol-to-Olefin (MTO) Reaction by P or Zn Modifications. Catal. Today 2020, 348, 243–256. 10.1016/j.cattod.2019.07.059.

[ref13] Yarulina I.; De Wispelaere K.; Bailleul S.; Goetze J.; Radersma M.; Abou-Hamad E.; Vollmer I.; Goesten M.; Mezari B.; Hensen E. J. M.; Martínez-Espín J. S.; Morten M.; Mitchell S.; Perez-Ramirez J.; Olsbye U.; Weckhuysen B. M.; Van Speybroeck V.; Kapteijn F.; Gascon J. Structure–Performance Descriptors and the Role of Lewis Acidity in the Methanol-to-Propylene Process. Nat. Chem. 2018, 10, 804–812. 10.1038/s41557-018-0081-0. 29941905

[ref14] Yarulina I.; Bailleul S.; Pustovarenko A.; Martinez J. R.; Wispelaere K. De; Hajek J.; Weckhuysen B. M.; Houben K.; Baldus M.; Van Speybroeck V.; Kapteijn F.; Gascon J. Suppression of the Aromatic Cycle in Methanol-to-Olefins Reaction over ZSM-5 by Post-Synthetic Modification Using Calcium. ChemCatChem. 2016, 8, 3057–3063. 10.1002/cctc.201600650.

[ref15] Spannhoff K.; Patcas F. C.; Bay K.; Gaab M.; Schwab E.; Hesse M. Katalysator Und Verfahren Für Die Umwandlung von Oxygenaten Zu Olefinen. WO 2014/001410, 2014.

[ref16] Gaab M.; Mueller U.; Kostur M.; Braunsmann K.; Bay K.; Parvulescu A.-N. Production and Use of a Zeolitic Material in a Process for the Conversion of Oxygenates to Olefins. WO/2014/076625, 2014.

[ref17] Heriyanto H.; Muraza O.; Nasser G. A.; Sanhoob M. A.; Bakare I. A.; Budhijanto; Rochmadi; Budiman A. Development of New Kinetic Models for Methanol to Hydrocarbons over a Ca-ZSM-5 Catalyst. Energy Fuels 2020, 34, 6245–6260. 10.1021/acs.energyfuels.9b04327.

[ref18] Rostamizadeh M.; Taeb A. Highly Selective Me-ZSM-5 Catalyst for Methanol to Propylene (MTP). J. Ind. Eng. Chem. 2015, 27, 297–306. 10.1016/j.jiec.2015.01.004.

[ref19] Goetze J.; Weckhuysen B. M. Spatiotemporal Coke Formation over Zeolite ZSM-5 during the Methanol-to-Olefins Process as Studied with: Operando UV-Vis Spectroscopy: A Comparison between H-ZSM-5 and Mg-ZSM-5. Catal. Sci. Technol. 2018, 8, 1632–1644. 10.1039/C7CY02459B.

[ref20] Salmasi M.; Fatemi S.; Najafabadi A. T. Improvement of Light Olefins Selectivity and Catalyst Lifetime in MTO Reaction; Using Ni and Mg-Modified SAPO-34 Synthesized by Combination of Two Templates. J. Ind. Eng. Chem. 2011, 17, 755–761. 10.1016/j.jiec.2011.05.031.

[ref21] Chen C.; Zhang Q.; Meng Z.; Li C.; Shan H. Effect of Magnesium Modification over H-ZSM-5 in Methanol to Propylene Reaction. Appl. Petrochemical Res. 2015, 5, 277–284. 10.1007/s13203-015-0129-7.

[ref22] Ji Y.; Birmingham J.; Deimund M. A.; Brand S. K.; Davis M. E. Steam-Dealuminated, OSDA-Free RHO and KFI-Type Zeolites as Catalysts for the Methanol-to-Olefins Reaction. Microporous Mesoporous Mater. 2016, 232, 126–137. 10.1016/j.micromeso.2016.06.012.

[ref23] Schmidt J. E.; Peng L.; Poplawsky J. D.; Weckhuysen B. M. Nanoscale Chemical Imaging of Zeolites Using Atom Probe Tomography. Angew. Chem., Int. Ed. 2018, 57, 10422–10435. 10.1002/anie.201712952. PMC6519151 29718553

[ref24] Mentzel U. V.; Ho̷jholt K. T.; Holm M. S.; Fehrmann R.; Beato P. Conversion of Methanol to Hydrocarbons over Conventional and Mesoporous H-ZSM-5 and H-Ga-MFI: Major Differences in Deactivation Behavior. Appl. Catal. A Gen. 2012, 417–418, 290–297. 10.1016/j.apcata.2012.01.003.

[ref25] Schmidt J. E.; Peng L.; Paioni A. L.; Ehren H. L.; Guo W.; Mazumder B.; Matthijs De Winter D. A.; Attila Ö.; Fu D.; Chowdhury A. D.; Houben K.; Baldus M.; Poplawsky J. D.; Weckhuysen B. M. Isolating Clusters of Light Elements in Molecular Sieves with Atom Probe Tomography. J. Am. Chem. Soc. 2018, 140, 9154–9158. 10.1021/jacs.8b04494. 30003782 PMC6065070

[ref26] Thomas J. M.; Terasaki O.; Gai P. L.; Zhou W.; Gonzalez-Calbet J. Structural Elucidation of Microporous and Mesoporous Catalysts and Molecular Sieves by High-Resolution Electron Microscopy. Acc. Chem. Res. 2001, 34, 583–594. 10.1021/ar970352j. 11456476

[ref27] Mayoral A.; Zhang Q.; Zhou Y.; Chen P.; Ma Y.; Monji T.; Losch P.; Schmidt W.; Sch F.; Hirao H.; Yu J.; Terasaki O. Atomic-Level Imaging of Zeolites: Oxygen, Sodium in Na-LTA and Iron in Fe-MFI. Angew. Chem., Int. Ed. 2020, 59, 19510–19517. 10.1002/anie.202006122. PMC7689718 32542978

[ref28] McGuire R.; Wechsung A.; Kuretschka C.; Ivana J.; Kuschel A.; Schunk S. A. A Composition Comprising a Mixed Metal Oxide and a Molding Comprising a Zeolitic Material Having Framework Type CHA and an Alkaline Earth Metal. WO/2019/030279, 2019.

[ref29] McGuire R.; Kuretschka C.; Müller U.; Schwab E. Catalyst Composite Comprising an Alkaline Earth Metal Containing CHA Zeolite and Use Thereof in a Process for the Conversion of Oxygenates to Olefins. WO/2018/096171, 2018.

[ref30] Rivera-Ramos M. E.; Hernández-Maldonado A. J. Adsorption of N_2_ and CH_4_ by Ion-Exchanged Silicoaluminophosphate Nanoporous Sorbents: Interaction with Monovalent, Divalent, and Trivalent Cations. Ind. Eng. Chem. Res. 2007, 46, 4991–5002. 10.1021/ie061016m.

[ref31] Zhu D.; Wang Z.; Meng F.; Zhao B.; Kanitkar S.; Tang Y. Catalytic Conversion of Chloromethane to Olefins and Aromatics Over Zeolite Catalysts. Catal. Lett. 2021, 151, 1038–1048. 10.1007/s10562-020-03364-z.

[ref32] Oord R.; ten Have I. C.; Arends J. M.; Hendriks F. C.; Schmidt J.; Lezcano-Gonzalez I.; Weckhuysen B. M. Enhanced Activity of Desilicated Cu-SSZ-13 for the Selective Catalytic Reduction of NOx and Its Comparison with Steamed Cu-SSZ-13. Catal. Sci. Technol. 2017, 7, 3851–3862. 10.1039/C7CY00798A.

[ref33] Goetze J.; Meirer F.; Yarulina I.; Gascon J.; Kapteijn F.; Ruiz-Martínez J.; Weckhuysen B. M. Insights into the Activity and Deactivation of the Methanol-to-Olefins Process over Different Small-Pore Zeolites As Studied with Operando UV-Vis Spectroscopy. ACS Catal. 2017, 7, 4033–4046. 10.1021/acscatal.6b03677. 28603658 PMC5460665

[ref34] Borodina E.; Sharbini Harun Kamaluddin H.; Meirer F.; Mokhtar M.; Asiri A. M.; Al-Thabaiti S. A.; Basahel S. N.; Ruiz-Martinez J.; Weckhuysen B. M. Influence of the Reaction Temperature on the Nature of the Active and Deactivating Species during Methanol-to-Olefins Conversion over H-SAPO-34. ACS Catal. 2017, 7, 5268–5281. 10.1021/acscatal.7b01497. 28824823 PMC5557614

[ref35] Borodina E.; Meirer F.; Lezcano-González I.; Mokhtar M.; Asiri A. M.; Al-Thabaiti S. A.; Basahel S. N.; Ruiz-Martinez J.; Weckhuysen B. M. Influence of the Reaction Temperature on the Nature of the Active and Deactivating Species during Methanol to Olefins Conversion over H-SSZ-13. ACS Catal. 2015, 5, 992–1003. 10.1021/cs501345g. PMC5557614 28824823

[ref36] Cats K. H.; Weckhuysen B. M. Combined Operando X-Ray Diffraction/Raman Spectroscopy of Catalytic Solids in the Laboratory: The Co/TiO_2_ Fischer – Tropsch Synthesis Catalyst Showcase. ChemCatChem. 2016, 8, 1531–1542. 10.1002/cctc.201600074. 27812371 PMC5069592

[ref37] Goetze J.; Yarulina I.; Gascon J.; Kapteijn F.; Weckhuysen B. M. Revealing Lattice Expansion of Small-Pore Zeolite Catalysts during the Methanol-to-Olefins Process Using Combined Operando X-Ray Diffraction and UV-Vis Spectroscopy. ACS Catal. 2018, 8, 2060–2070. 10.1021/acscatal.7b04129. 29527401 PMC5839605

[ref38] van Vreeswijk S. H.; Monai M.; Oord R.; Schmidt J. E.; Vogt E. T. C.; Poplawsky J. D.; Weckhuysen B. M. Nano-Scale Insights Regarding Coke Formation in Zeolite SSZ-13 Subject to the Methanol-to- Hydrocarbons Reaction. Catal. Sci. Technol. 2022, 12, 1220–1228. 10.1039/d1cy01938d. 35310769 PMC8859524

[ref39] Schmidt J. E.; Poplawsky J. D.; Mazumder B.; Attila O.; Fu D.; de Winter D. A. M.; Meirer F.; Bare S. R.; Weckhuysen B. M. Coke Formation in a Zeolite Crystal During the Methanol-to-Hydrocarbons Reaction as Studied with Atom Probe Tomography. Angew. Chem., Int. Ed. 2016, 55, 11173–11177. 10.1002/anie.201606099. PMC6681177 27485276

[ref40] Tang F.; Zhu T.; Oehler F.; Fu W. Y.; Griffiths J. T.; Massabuau F. C. P.; Kappers M. J.; Martin T. L.; Bagot P. A. J.; Moody M. P.; Oliver R. A. Indium Clustering in A-Plane InGaN Quantum Wells as Evidenced by Atom Probe Tomography. Appl. Phys. Lett. 2015, 106, 072104 10.1063/1.4909514.

[ref41] Stephenson L. T.; Moody M. P.; Liddicoat P. V.; Ringer S. P. New Techniques for the Analysis of Fine-Scaled Clustering Phenomena within Atom Probe Tomography (APT) Data. Microsc. Microanal. 2007, 13, 448–463. 10.1017/S1431927607070900. 18001511

[ref42] Philippe T.; De Geuser F.; Duguay S.; Lefebvre W.; Cojocaru-Mirédin O.; Da Costa G.; Blavette D. Clustering and Nearest Neighbour Distances in Atom-Probe Tomography. Ultramicroscopy 2009, 109, 1304–1309. 10.1016/j.ultramic.2009.06.007. 19592168

[ref43] Larson D. J.; Prosa T. J.; Ulfig R. M.; Geiser B. P.; Kelly T. F. Local Electrode Atom Probe Tomography; Springer: 2013.

[ref44] Booth-Morrison C.; Mao Z.; Diaz M.; Dunand D. C.; Wolverton C.; Seidman D. N. Role of Silicon in Accelerating the Nucleation of Al_3_(Sc,Zr) Precipitates in Dilute Al-Sc-Zr Alloys. Acta Mater. 2012, 60, 4740–4752. 10.1016/j.actamat.2012.05.036.

[ref45] Haley D.; Petersen T.; Barton G.; Ringer S. P. Influence of Field Evaporation on Radial Distribution Functions in Atom Probe Tomography. Philos. Mag. 2009, 89, 925–943. 10.1080/14786430902821610.

[ref46] Zhou J.; Odqvist J.; Thuvander M.; Hedström P. Quantitative Evaluation of Spinodal Decomposition in Fe-Cr by Atom Probe Tomography and Radial Distribution Function Analysis. Microsc. Microanal. 2008, 19, 666–675. 10.1017/S1431927613000470. 23642804

[ref47] Wang D.; Gao F.; Peden C. H. F.; Li J.; Kamasamudram K.; Epling W. S. Selective Catalytic Reduction of NO_x_ with NH_3_ over a Cu-SSZ-13 Catalyst Prepared by a Solid-State Ion-Exchange Method. ChemCatChem. 2014, 6, 1579–1583. 10.1002/cctc.201402010.

[ref48] Zhang T.; Qiu F.; Li J. Design and Synthesis of Core-Shell Structured Meso-Cu-SSZ-13@mesoporous Aluminosilicate Catalyst for SCR of NO_x_ with NH_3_: Enhancement of Activity, Hydrothermal Stability and Propene Poisoning Resistance. Appl. Catal. B Environ. 2016, 195, 48–58. 10.1016/j.apcatb.2016.04.058.

[ref49] Database of Zeolite Structures, https://europe.iza-structure.org/IZA-SC/framework_vol.php?STC=CHA. Accessed Aug 1, 2022.

[ref50] Moody M. P.; Stephenson L. T.; Ceguerra A. V.; Ringer S. P. Quantitative Binomial Distribution Analyses of Nanoscale Like-Solute Atom Clustering and Segregation in Atom Probe Tomography Data. Microsc. Res. Technol. 2008, 71, 542–550. 10.1002/jemt.20582. 18425800

[ref51] Wang N.; Zhi Y.; Wei Y.; Zhang W.; Liu Z.; Huang J.; Sun T.; Xu S.; Lin S.; He Y.; Zheng A.; Liu Z. Molecular Elucidating of an Unusual Growth Mechanism for Polycyclic Aromatic Hydrocarbons in Confined Space. Nat. Commun. 2020, 11, 1079 10.1038/s41467-020-14493-9. 32103001 PMC7044299

[ref52] Cheng K.; Smulders L. C. J.; van der Wal L. I.; Oenema J.; Meeldijk J. D.; Visser N. L.; Sunley G.; Roberts T.; Xu Z.; Doskocil E.; Yoshida H.; Zheng Y.; Zecevic J.; de Jongh P. E.; de Jong K. P. Maximizing Noble Metal Utilization in Solid Catalysts by Control of Nanoparticle Location. Science 2022, 377, 204–208. 10.1126/science.abn8289. 35857537

