# Supplementary material for: Detecting Cage Crossing and Filling Clusters of Magnesium and Carbon Atoms in Zeolite SSZ-13 with Atom Probe Tomography
Source: JACS Au. 2022 Oct 14;2(11):2501–13. doi: 10.1021/jacsau.2c00296 (PMC9709938; doi:10.1021/jacsau.2c00296)
Supplement: Supplementary file 1 — au2c00296_si_002.zip [file au2c00296_si_002.zip › vanVreeswijketal_SI_2022.docx]

Detecting Cage Crossing and Filling Clusters of Magnesium and Carbon Atoms in Zeolite SSZ-13 with Atom Probe Tomography

Sophie H. van Vreeswijk^1^, Matteo Monai^1^, Ramon Oord^1^, Joel E. Schmidt^1^, Andrei N. Parvulescu^3^, Irina Yarulina^3^, Lukasz Karwacki^3^, Jonathan D. Poplawsky^2,^* and Bert M. Weckhuysen^1,^*

*^1^ Inorganic Chemistry and Catalysis group, Debye Institute for Nanomaterials Science, Utrecht University, Universiteitsweg 99, 3854 CG Utrecht, The Netherlands*

*^2^ Center for Nanophase Materials Sciences, Oak Ridge National Laboratory, Oak Ridge, TN 37831, USA*

*^3^ BASF, Carl-Bosch-Straße 38, 67063 Ludwigshafen am Rhein, Germany*

* to whom correspondence should be addressed; [poplawskyjd@ornl.gov](mailto:poplawskyjd@ornl.gov) and b.m.weckhuysen@uu.nl

# Bulk Characterization

## Pore volume and composition of the material

**Table S 1**: Si/Al ratios, Mg content, Surface area and pore volume information

|  | Si/Al ratio* | Mg wt%* | S_BET_ (cm^2^g^-1^)** | V_micro_ (cm^3^g^-1^)** |
| --- | --- | --- | --- | --- |
| H-SSZ-13 | 17.8 | - | 0.85 | 0.31 |
| Mg-SSZ-13 | 18.9 | 1.6 | 0.76 | 0.28 |
| * As determined with inductively coupled plasma-optical emission spectroscopy (ICP-OES)  ** As determined with N_2_-physisorption | | | | |

## Morphology of the samples

Scanning electron microscopy (SEM) images of the zeolite H-SSZ-13 and Mg-SSZ-13 materials are shown, indicating that magnesium ions are clustered on the outside of the zeolite crystals. Additionally, evidence of the outside species being magnesium have been shown with energy dispersive X-ray (EDX) analysis.


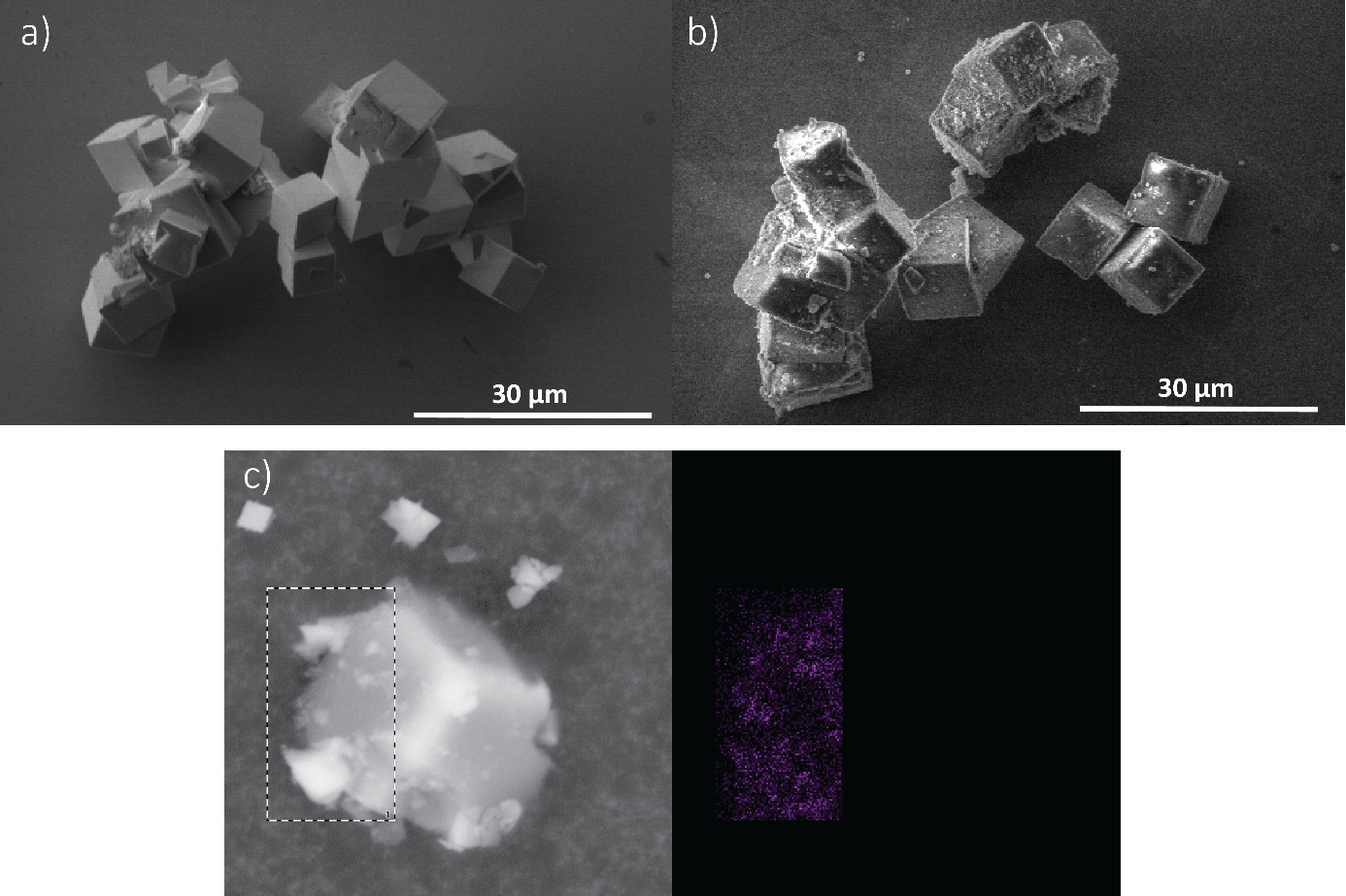


**Figure S1**: Scanning electron microscopy (SEM) images of a) H-SSZ-13 and b-c) Mg-SSZ-13 zeolites with c) Energy dispersive X-ray (EDX) analysis proof of magnesium on the outside of the zeolite crystal, although it is unclear what the exact penetration depth is of the X-rays in the zeolite material.

## Acidic properties of the zeolite

With ammonia-temperature programmed desorption (NH_3_-TPD) experiments the effect of magnesium on the acid sites of zeolite SSZ-13 have been studied. To semi-quantify the changes in zeolite acidity, the obtained NH_3_-TPD curves were fitted with three Gaussian curves, whilst identifying the low-temperature (LT) peaks, intermediate-temperature (IT) peaks, and high-temperature (HT) peaks.^1,2^ Important to notice, is that the use of such deconvolution can be quite arbitrary and is only used in this study to clarify the differences in acidity between the two catalysts.


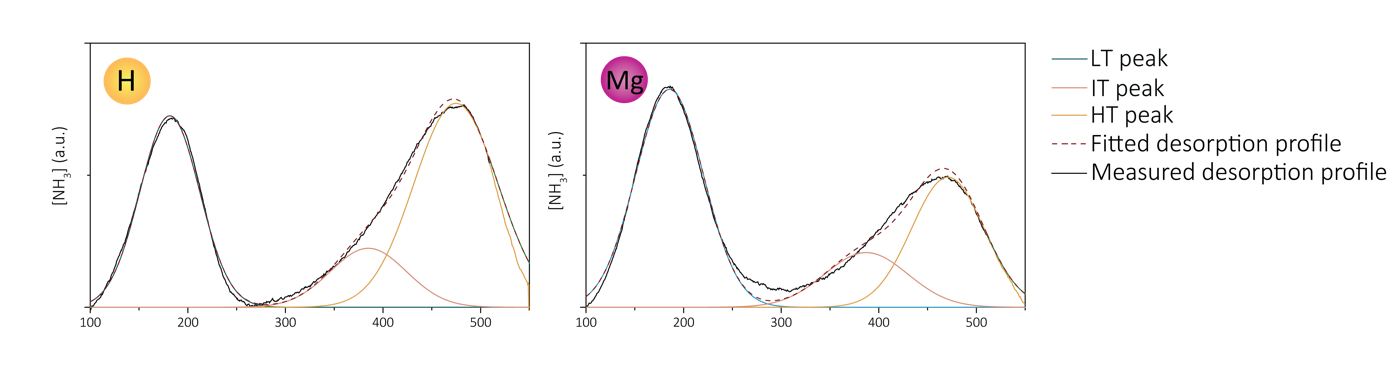


**Figure S2**: Fitted NH_3_-temperature programmed desorption (TPD)curves of H-SSZ-13 and Mg-SSZ-13 and the corresponding deconvolution results in three distinct contributions.

**Table S2**: Acidic properties of the two zeolite materials under study, as obtained by NH_3_ temperature programmed desorption (TPD) experiments.

|  | Quantity NH_3_ adsorbed (cm^3^g^-1^ STP) | mmol_NH3_/g_cat_ | LT peak contribution | | IT peak contribution | | HT peak contribution | |
| --- | --- | --- | --- | --- | --- | --- | --- | --- |
|  |  |  | °C | mmol_NH3_/g_cat_ | °C | mmol_NH3_/g_cat_ | °C | mmol_NH3_/g_cat_ |
| H-SSZ-13 | 19.1 | 0.86 | 182 | 0.29 | 384 | 0.12 | 475 | 0.44 |
| Mg-SSZ-13 | 19.7 | 0.87 | 182 | 0.45 | 387 | 0.14 | 471 | 0.28 |

# Atom Probe Tomography

## Pristine Mg-SSZ-13

### Atom Maps of the Pristine Zeolite Mg-SSZ-13 Sample

Three pristine Mg-SSZ-13 materials were successfully reconstructed. Two of these samples contained magnesium, while one sample did not.

###
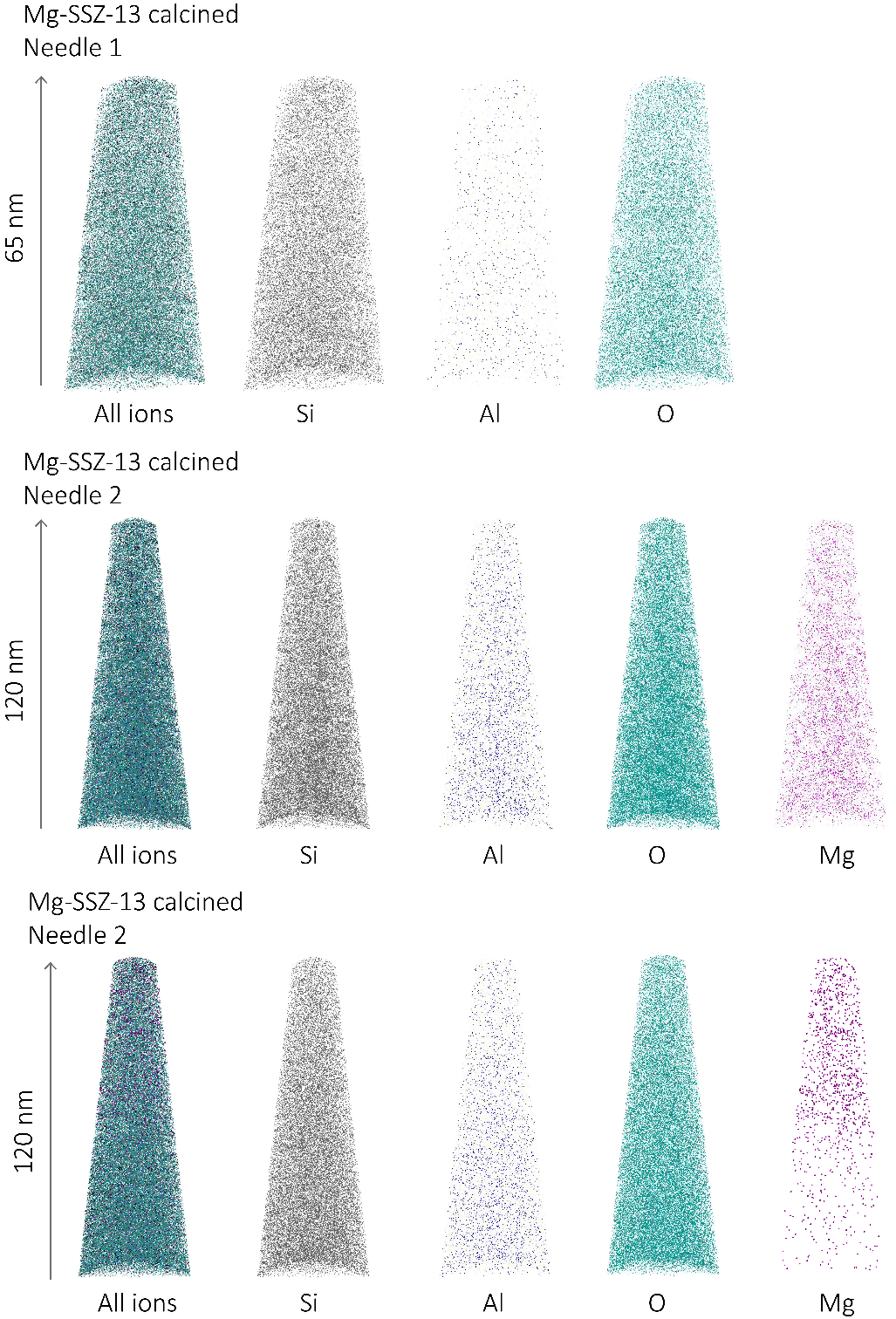
Elemental Composition of the Pristine Zeolite Mg-SSZ-13 Sample

**Figure S3**: Reconstructed atom probe tomography (APT) maps of the pristine Mg-SSZ-13 sample under study.

**Table S3**: APT dataset compositions of the pristine Mg-SSZ-13 sample.

|  | Dataset 1 | | Dataset 2 | | Dataset 3 | |
| --- | --- | --- | --- | --- | --- | --- |
| **Ion** | Count | Contribution (%) | Count | Contribution (%) | Count | Contribution (%) |
| O | 141975 | 61.704% | 611700 | 61.559% | 554862 | 62.062% |
| Si | 84775 | 36.844% | 342926 | 34.511% | 325690 | 36.429% |
| Al | 3067 | 1.333% | 14208 | 1.430% | 11318 | 1.266% |
| Ga | 272 | 0.118% | 579 | 0.058% | 1500 | 0.168% |
| Mg | - | - | 24260 | 2.441% | 678 | 0.076% |
|  |  |  |  |  |  |  |
|  | Si/Al | 27.6 | Si/Al | 24.1 | Si/Al | 28.8 |

### Nearest Neighbour Distribution Analysis of the Pristine Zeolite Mg-SSZ-13


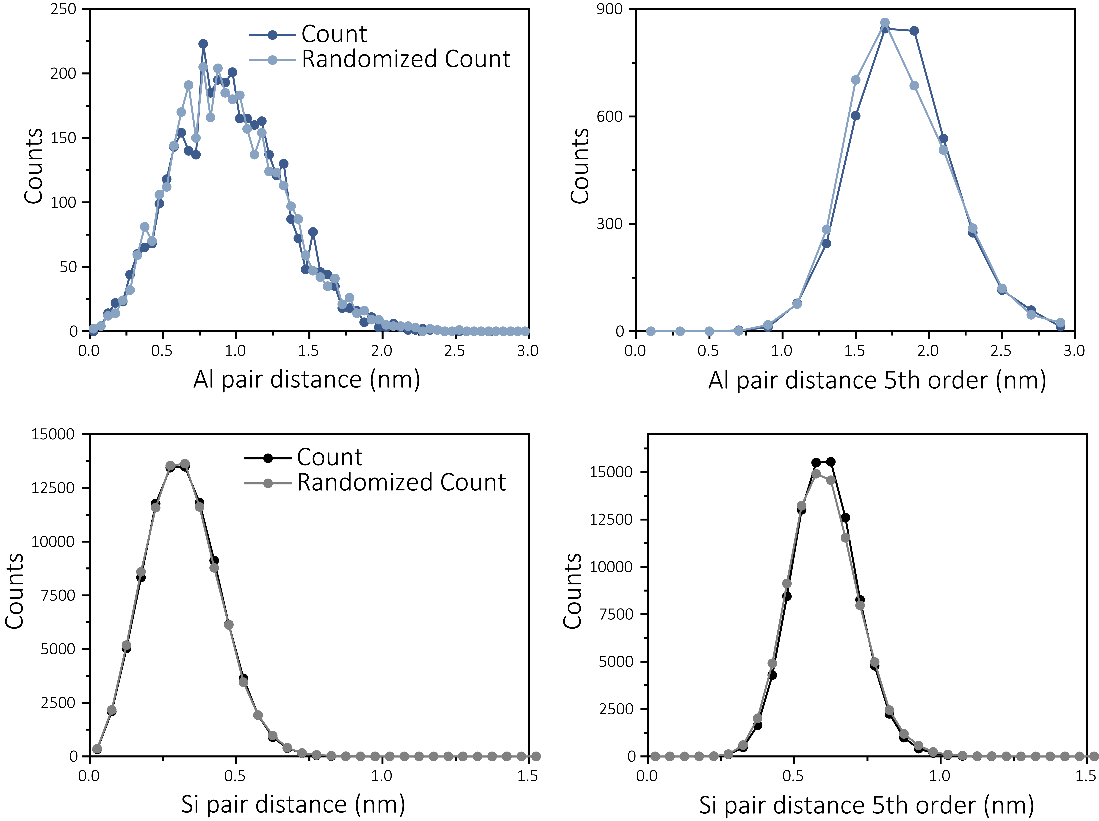


**Figure S4:** Nearest neighbour distributions (NND) analysis of the different elements of the pristine Mg-SSZ-13 Dataset 1.


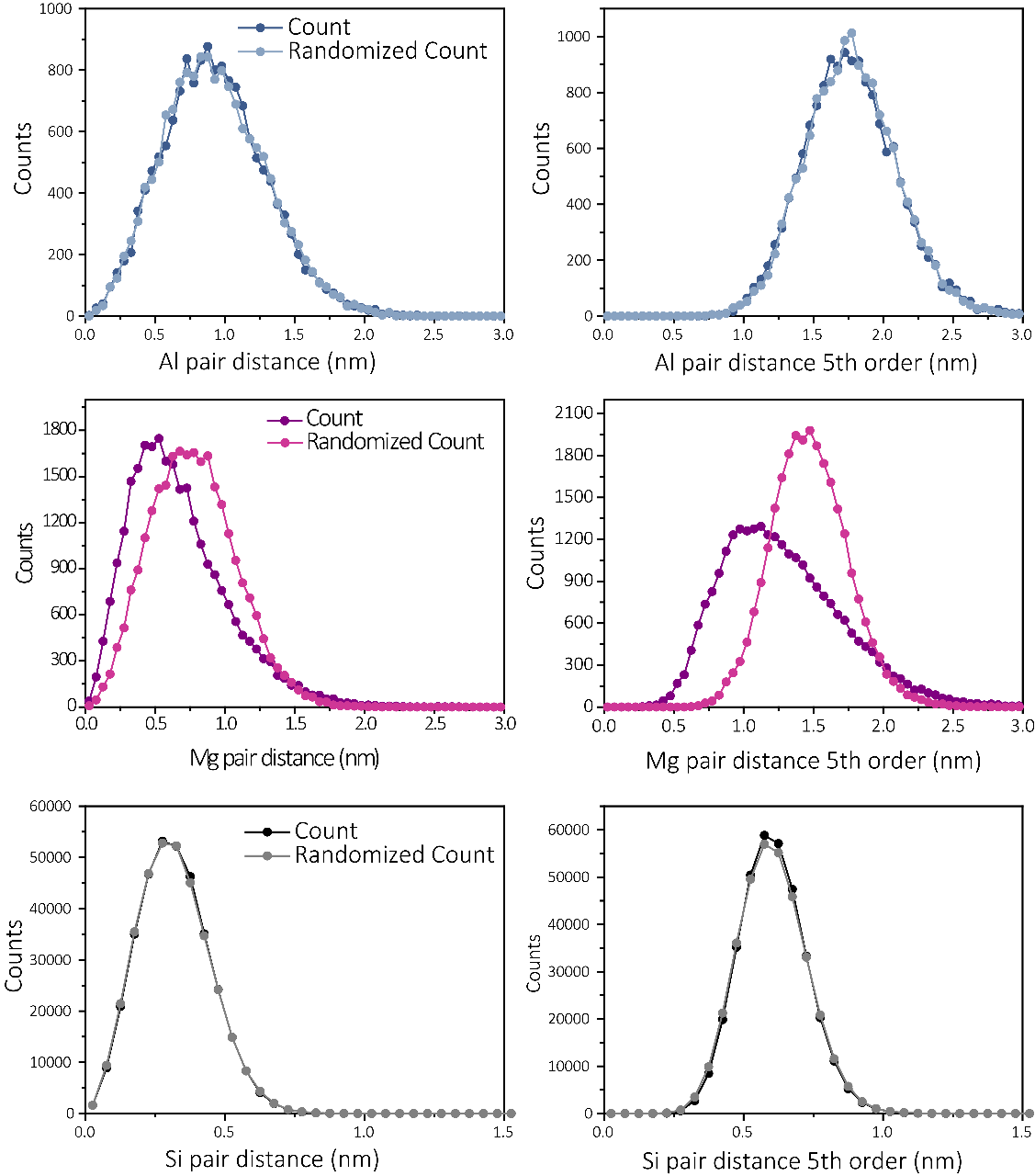


**Figure S5:** Nearest neighbour distributions (NND) analysis of the different elements of the pristine Mg-SSZ-13 Dataset 2.


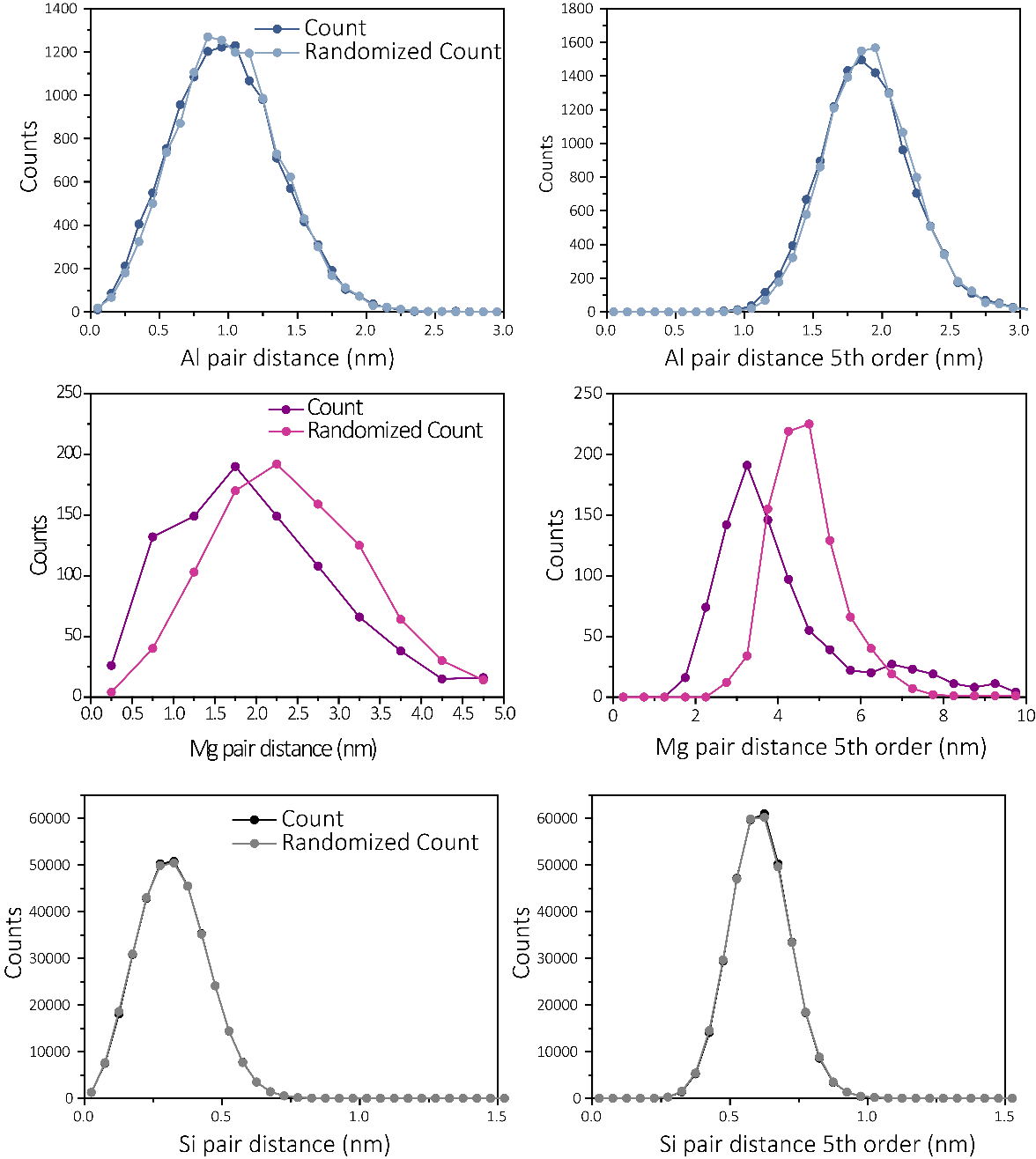


**Figure S6**: Nearest neighbour distributions (NND) analysis of the different elements of the pristine Mg-SSZ-13 Dataset 3.

### Radial Distribution Function Analysis of the Pristine Zeolite Mg-SSZ-13


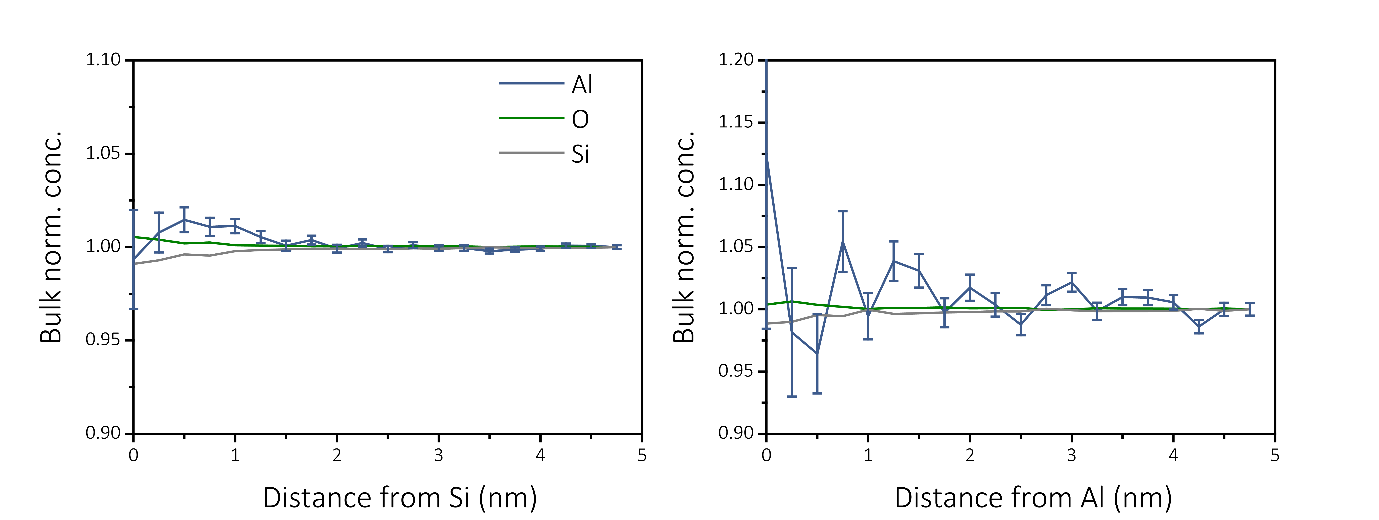


**Figure S7:** Radial distribution function (RDF) analysis of the pristine Mg-SSZ-13 Dataset 1.


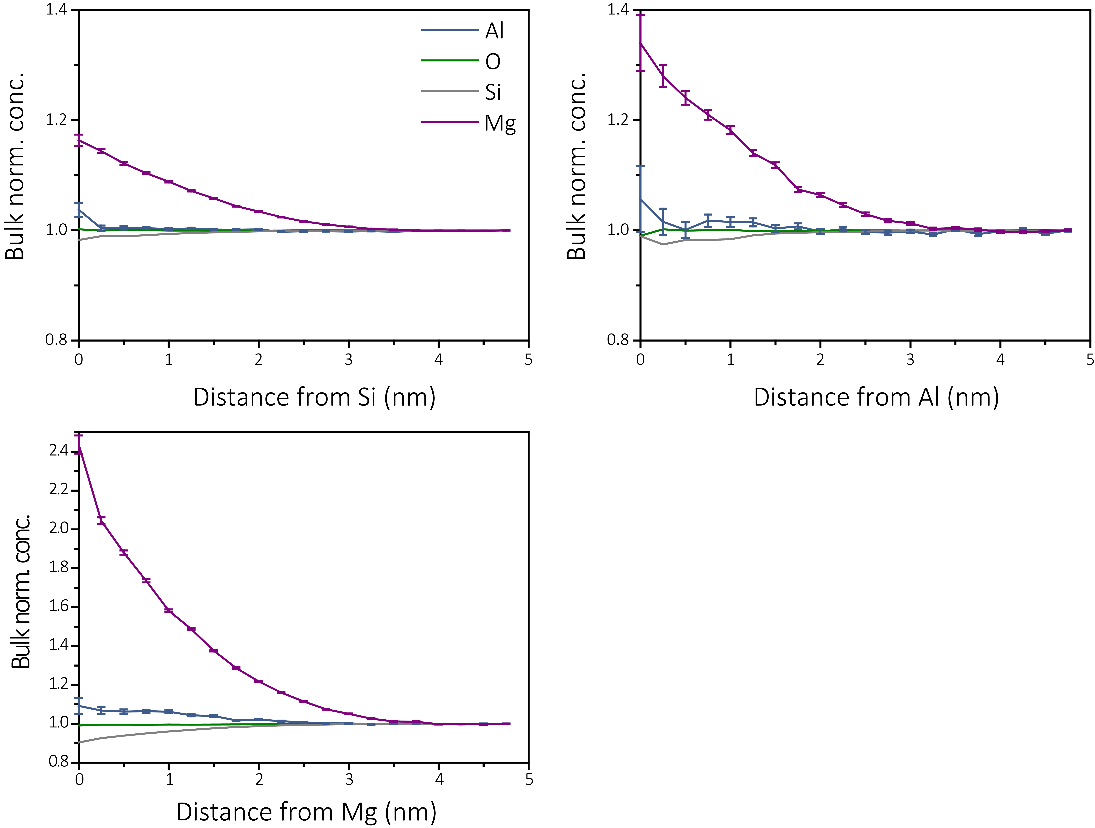


**Figure S8**: Radial distribution function (RDF) analysis of the pristine Mg-SSZ-13 Dataset 2.


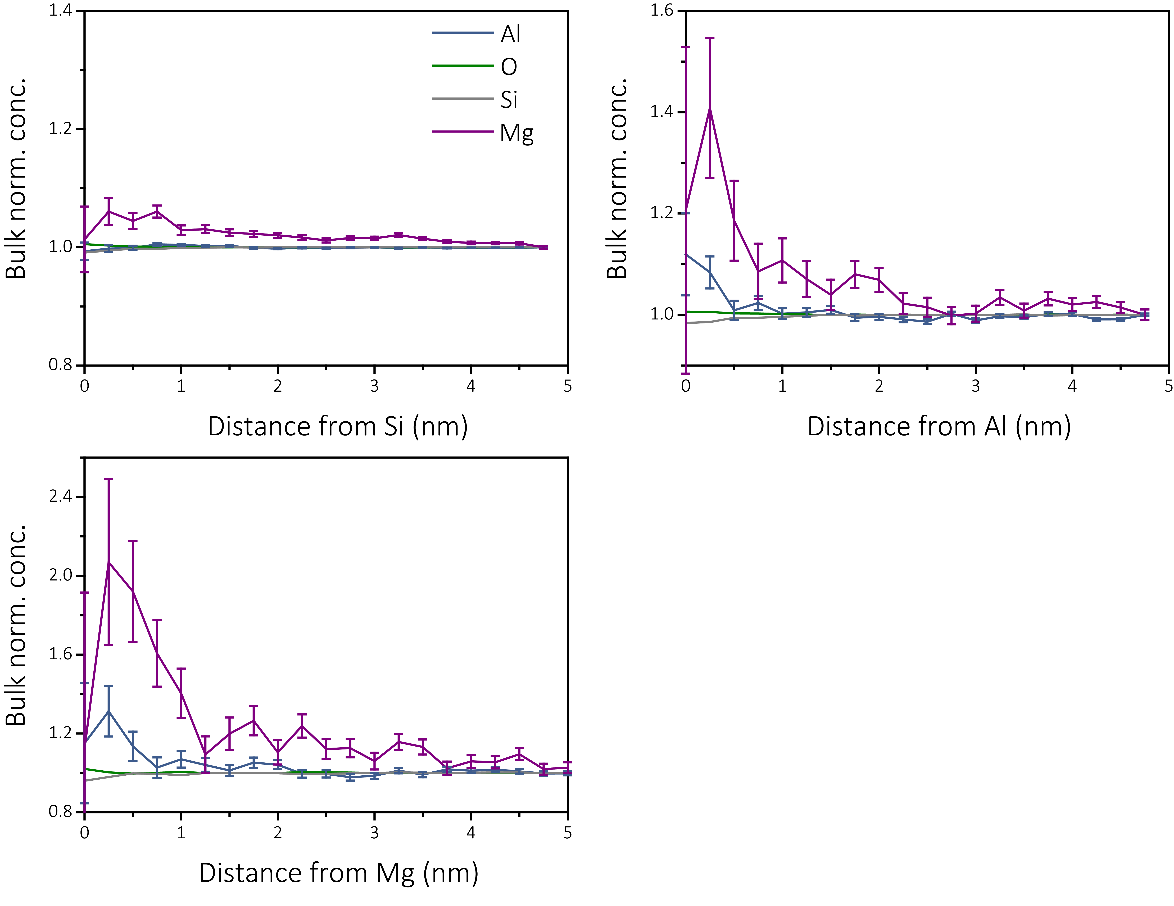


**Figure S9:** Radial distribution function (RDF) analysis of the pristine Mg-SSZ-13 Dataset 3.

### Iso-Surface Analysis of the Pristine Zeolite Mg-SSZ-13 Sample

###
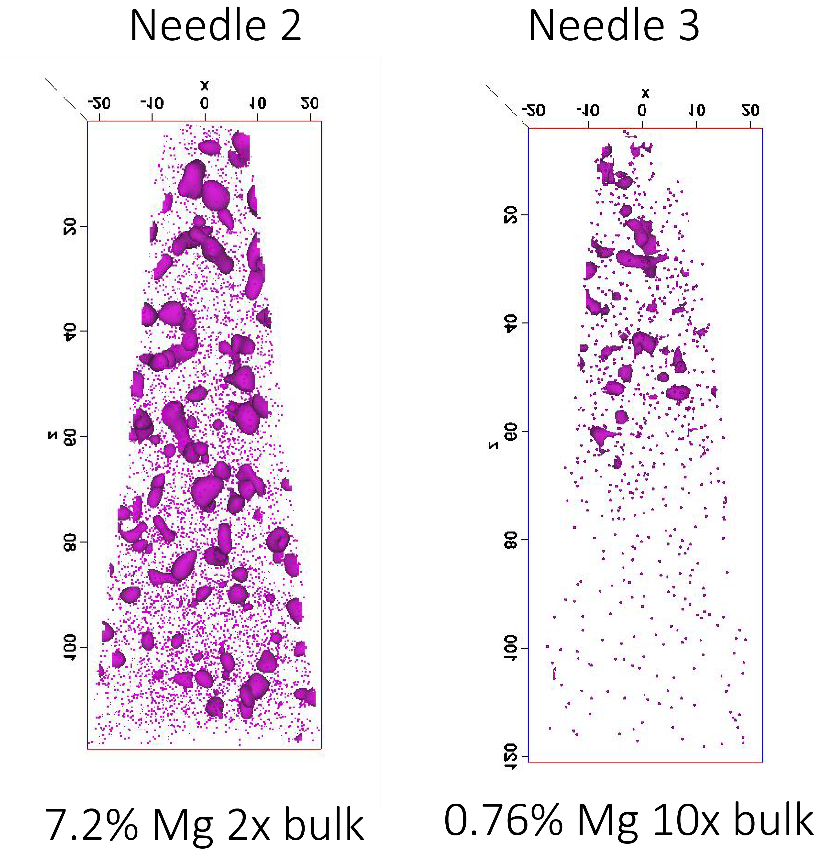


**Figure S10**: Iso-surface analysis of Mg of the fresh/calcined Mg-SSZ-13 Dataset 2 and 3.

### Cluster Analysis of the Pristine Zeolite Mg-SSZ-13 Sample


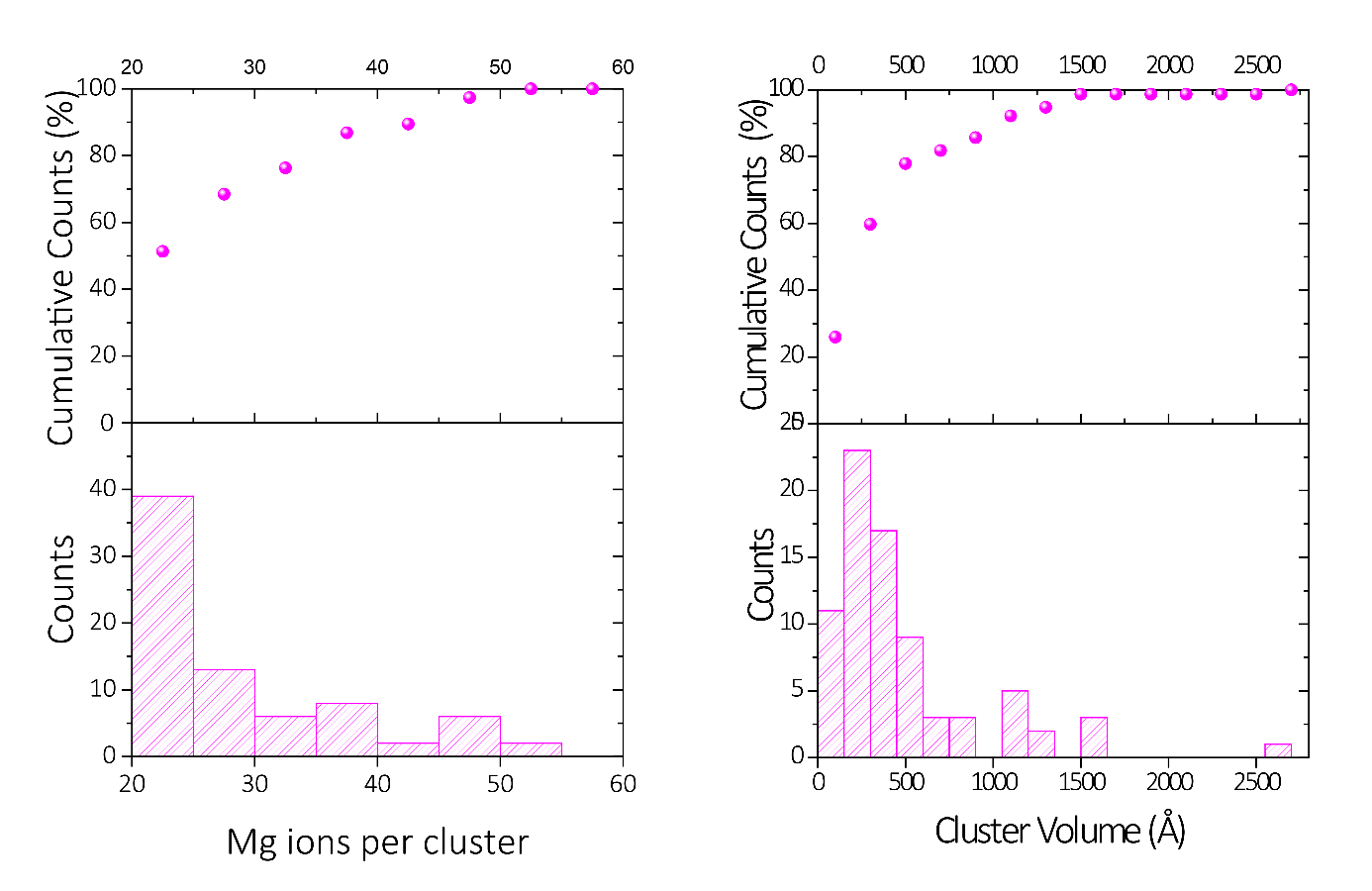


**Figure S11**: Number of Mg ions per cluster and the cluster volume found for the clusters in fresh/calcined Mg-SSZ-13 Dataset 2.

## Spent Zeolite Mg-SSZ-13

### Atom Probe Tomography 3-D Maps of the Spent Zeolite Mg-SSZ-13 Sample

Just as for the pristine Mg-SSZ-13 sample, not all Datasets contain magnesium. Similarly, not all datasets contain carbon.


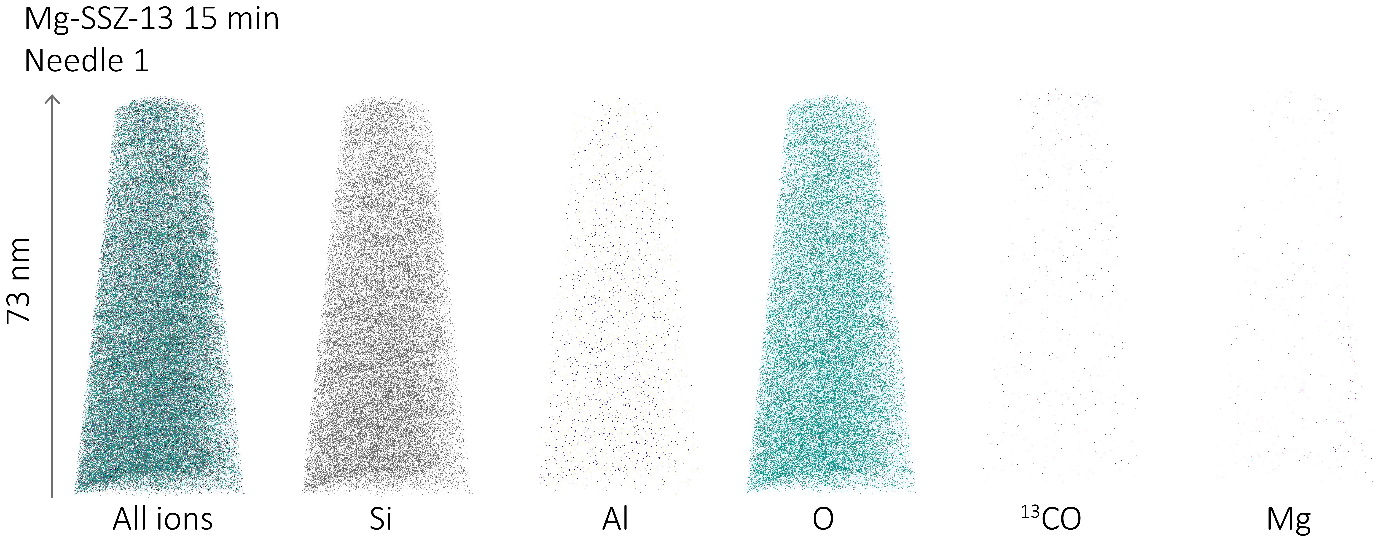


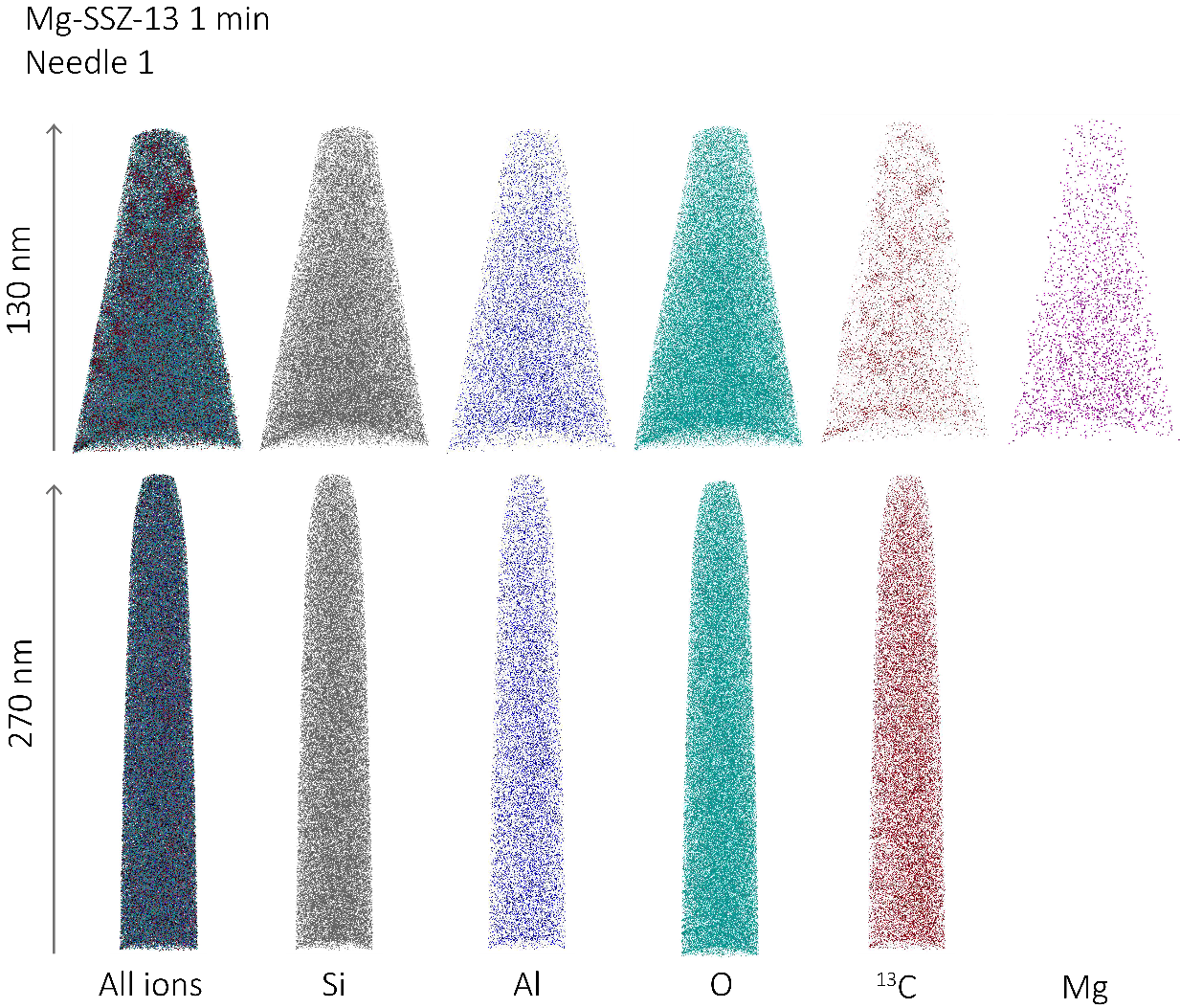


**Figure S12**: Reconstructed atom probe tomography (APT) 3-D maps of spent Mg-SSZ-13 1-min coked during the methanol-to-hydrocarbons (MTH) process.

**Figure S13**: Reconstructed atom probe tomography (APT) 3-D maps of spent Mg-SSZ-13 15-min coked during the methanol-to-hydrocarbons (MTH) process.


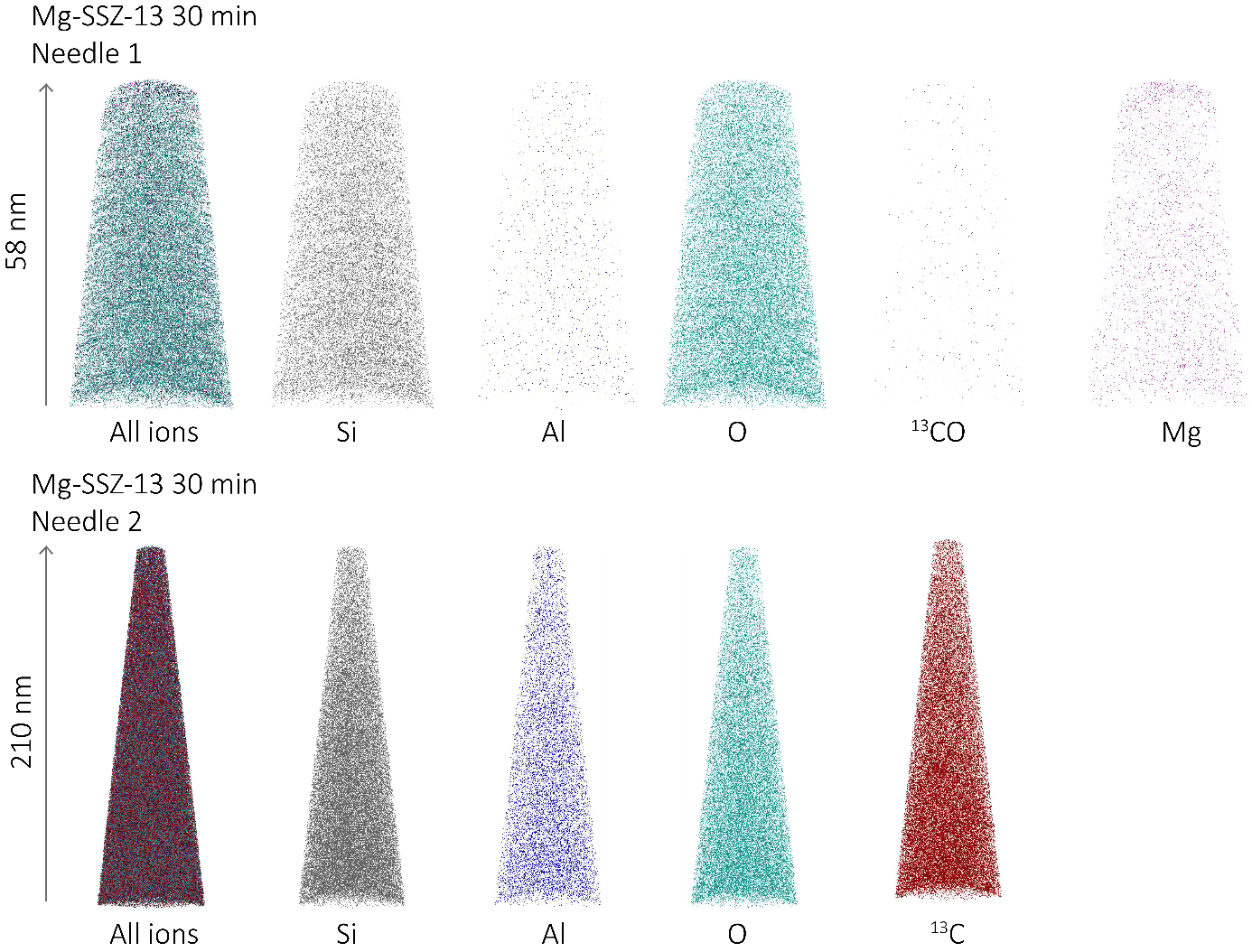


**Figure S14**: Reconstructed atom probe tomography (APT) 3-D maps of spent Mg-SSZ-13 30-min coked during the methanol-to-hydrocarbons (MTH) process.


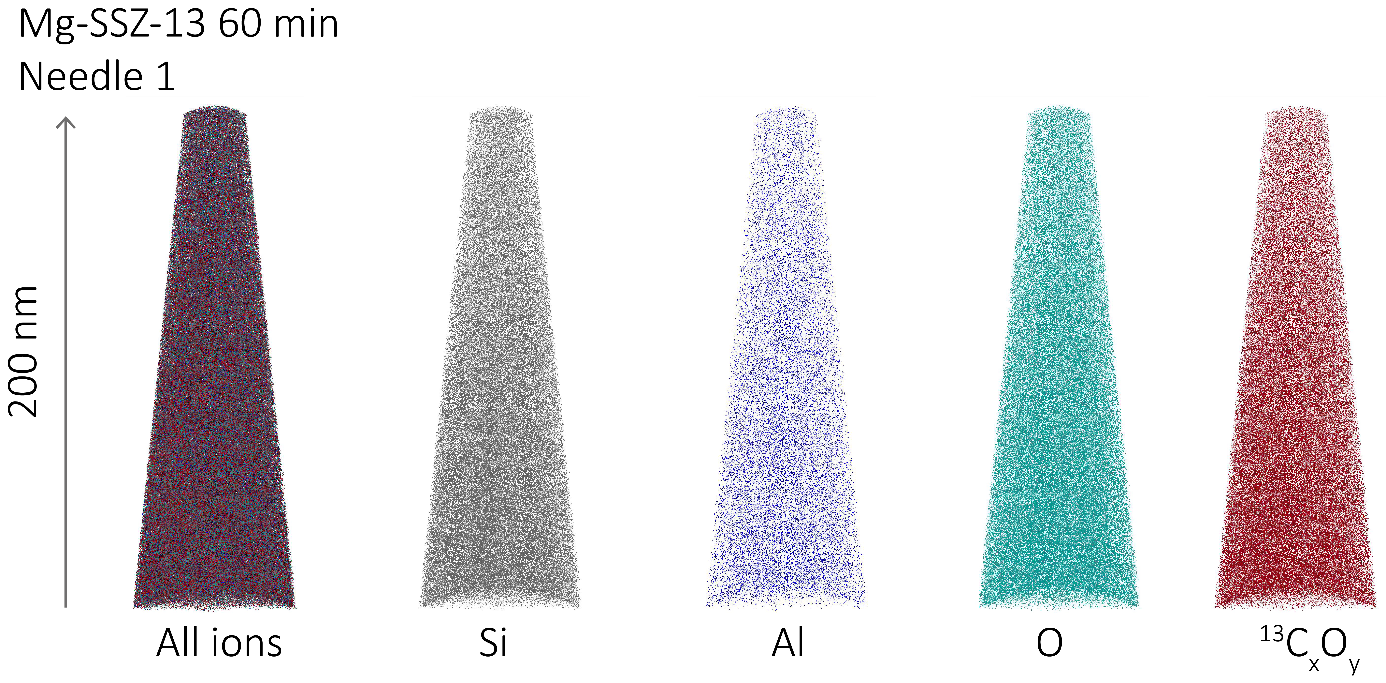


**Figure S15**: Reconstructed atom probe tomography (APT) maps of spent MgSSZ-13 60-min coked during the methanol-to-hydrocarbons (MTH) process.

### Compositions of the Spent MgSSZ-13 Sample

**Table S4**: Dataset composition of spent Mg-SSZ-13 coked 1-min during the methanol-to-hydrocarbons (MTH) process.

|  | Dataset 1 | | Dataset 2 | |
| --- | --- | --- | --- | --- |
| Ion | Count | Contribution (%) | Count | Contribution (%) |
| O | 1627456 | 61.380% | 1075974 | 55.336% |
| Si | 968366 | 36.523% | 773178 | 39.764% |
| Al | 32388 | 1.222% | 24288 | 1.249% |
| 13C | 20158 | 0.760% | 33267 | 1.711% |
| Mg | 1738 | 0.066% | 1494 | 0.077% |
| Ga | 1317 | 0.050% | 4248 | 0.218% |
|  |  |  |  |  |
|  | Si/Al | 29.9 | Si/Al | 31.8 |

**Table S5**: Dataset composition of spent Mg-SSZ-13 coked 15-min during the methanol-to-hydrocarbons (MTH) process.

|  | Dataset 1 | |
| --- | --- | --- |
| **Ion** | Count | Contribution (%) |
| O | 554862 | 62.062% |
| Si | 325690 | 36.429% |
| Al | 11318 | 1.266% |
| Ga | 1500 | 0.168% |
| Mg | 678 | 0.076% |
|  |  |  |
|  | Si/Al | 28.8 |

**Table S6**: Dataset composition of spent Mg-SSZ-13 coked 30-min during the methanol-to-hydrocarbons (MTH) process.

|  | Dataset 1 | | Dataset 2 | |
| --- | --- | --- | --- | --- |
| **Ion** | Count | Contribution (%) | Count | Contribution (%) |
| O | 99578 | 60.073% | 1893550 | 55.230% |
| Si | 58223 | 35.125% | 956750 | 27.906% |
| 13C | 246 | 0.148% | 398713 | 11.629% |
| 12C |  |  | 92300 | 2.692% |
| Mg | 4991 | 3.011% | 231 | 0.007% |
| Al | 2272 | 1.371% | 36371 | 1.061% |
| Ga | 450 | 0.272% | 922 | 0.027% |
| H |  |  | 49384 | 1.440% |
|  |  |  |  |  |
|  | Si/Al | 25.6 | Si/Al | 26.3 |
|  |  |  |  |  |

**Table S7**: Dataset composition of spent MgSSZ-13 coked 60-min during the methanol-to-hydrocarbons (MTH) process.

|  | Dataset 1 | |
| --- | --- | --- |
| **Ion** | Count | Ranged % |
| O | 2240084 | 55.328% |
| Si | 1126476 | 27.823% |
| 13C | 502400 | 12.409% |
| C | 77706 | 1.919% |
| H | 55498 | 1.371% |
| Al | 45803 | 1.131% |
| Ga | 748 | 0.018% |
|  | Si/Al | 24.6 |

### Nearest Neighbour Distributions of the Spent Zeolite MgSSZ-13 Sample


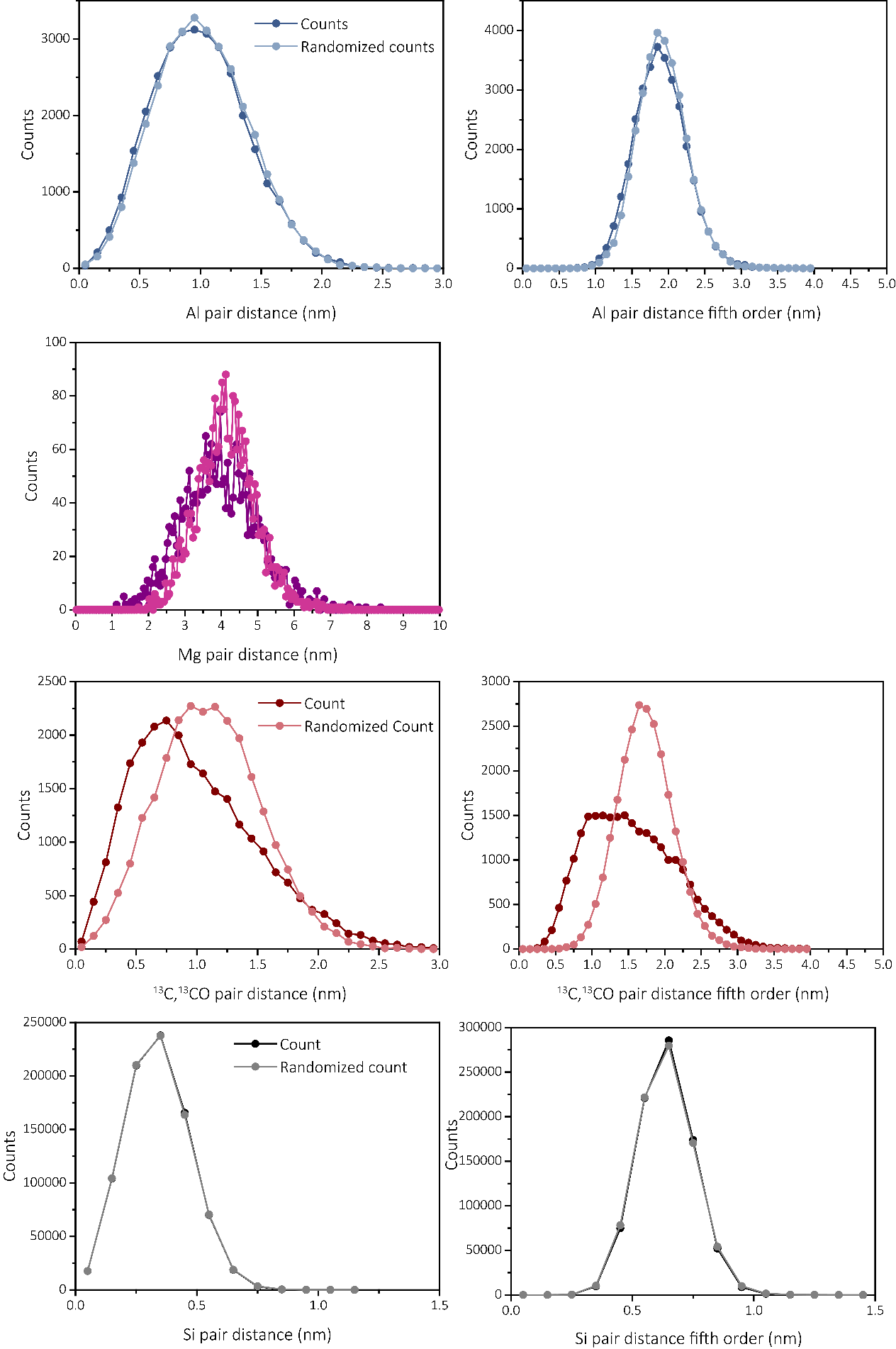


**Figure S16**: Nearest neighbour distribution (NND) of different elements of spent Mg-SZZ-13 coked 1-min sample dataset 1.


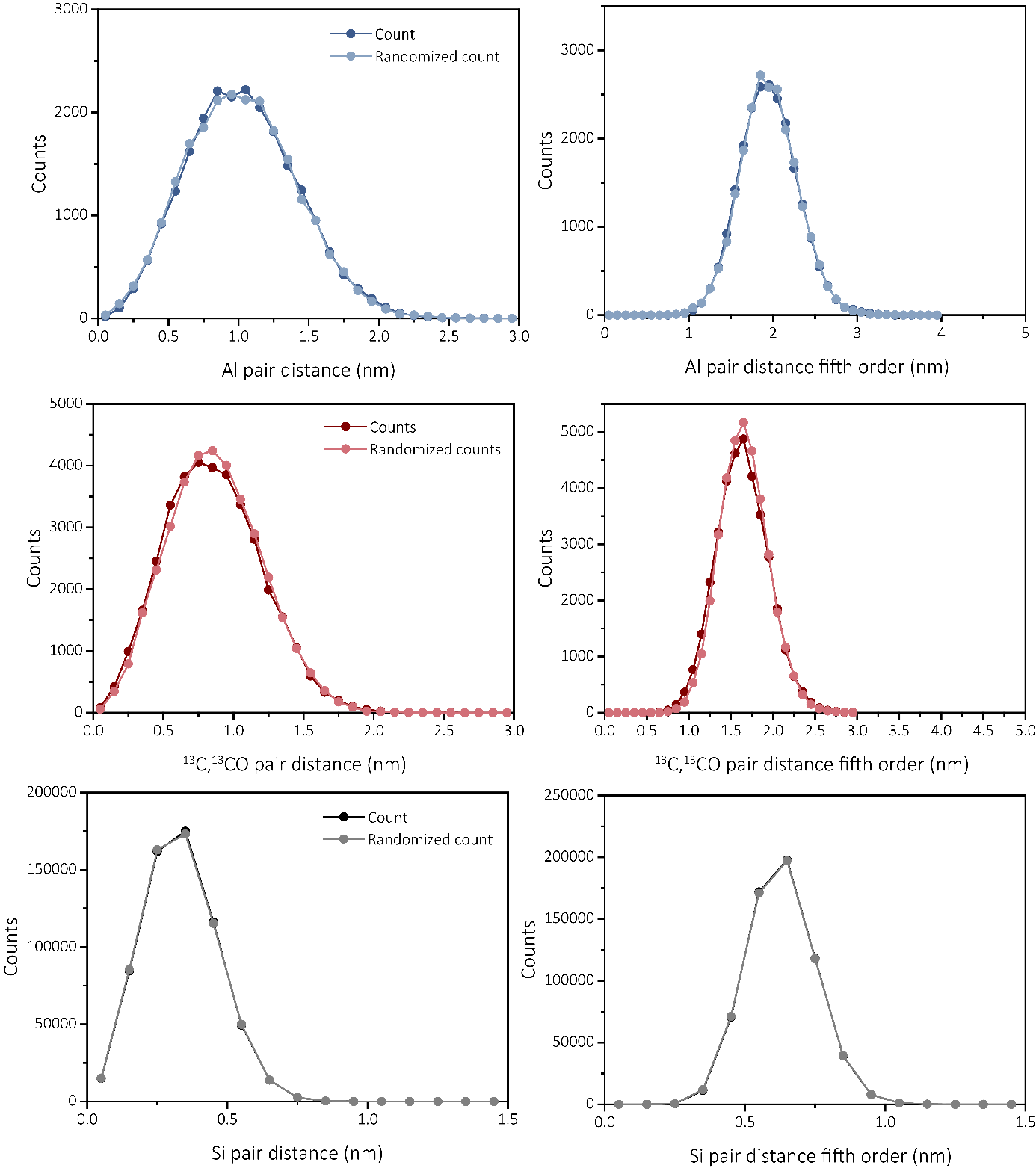


**Figure S17**: Nearest neighbour distribution (NND) of different elements of spent Mg-SZZ-13 coked 1-min sample dataset 2.


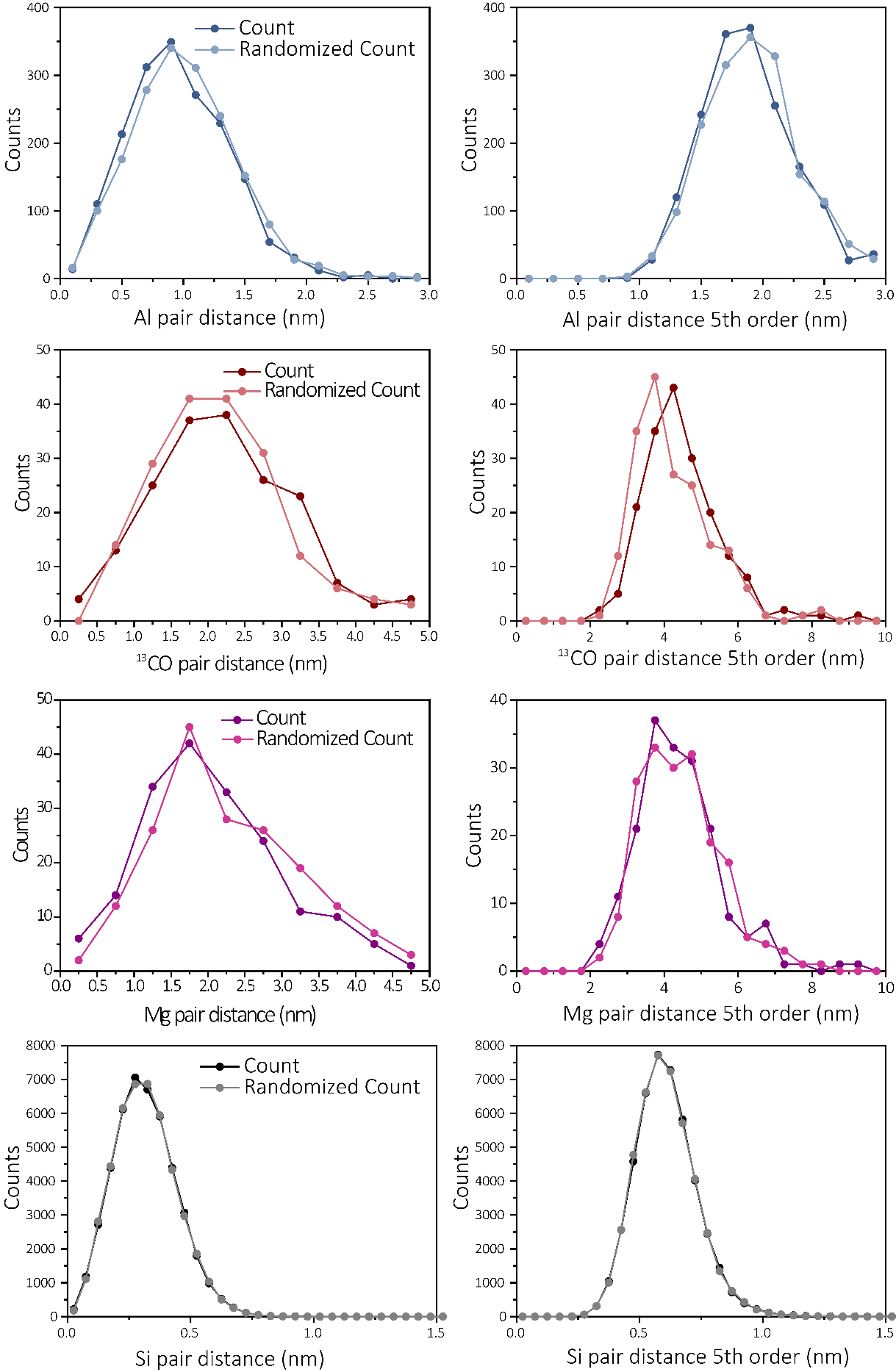


**Figure S18**: Nearest neighbour distribution (NND) of different elements of spent Mg-SZZ-13 coked 15-min sample dataset 1.


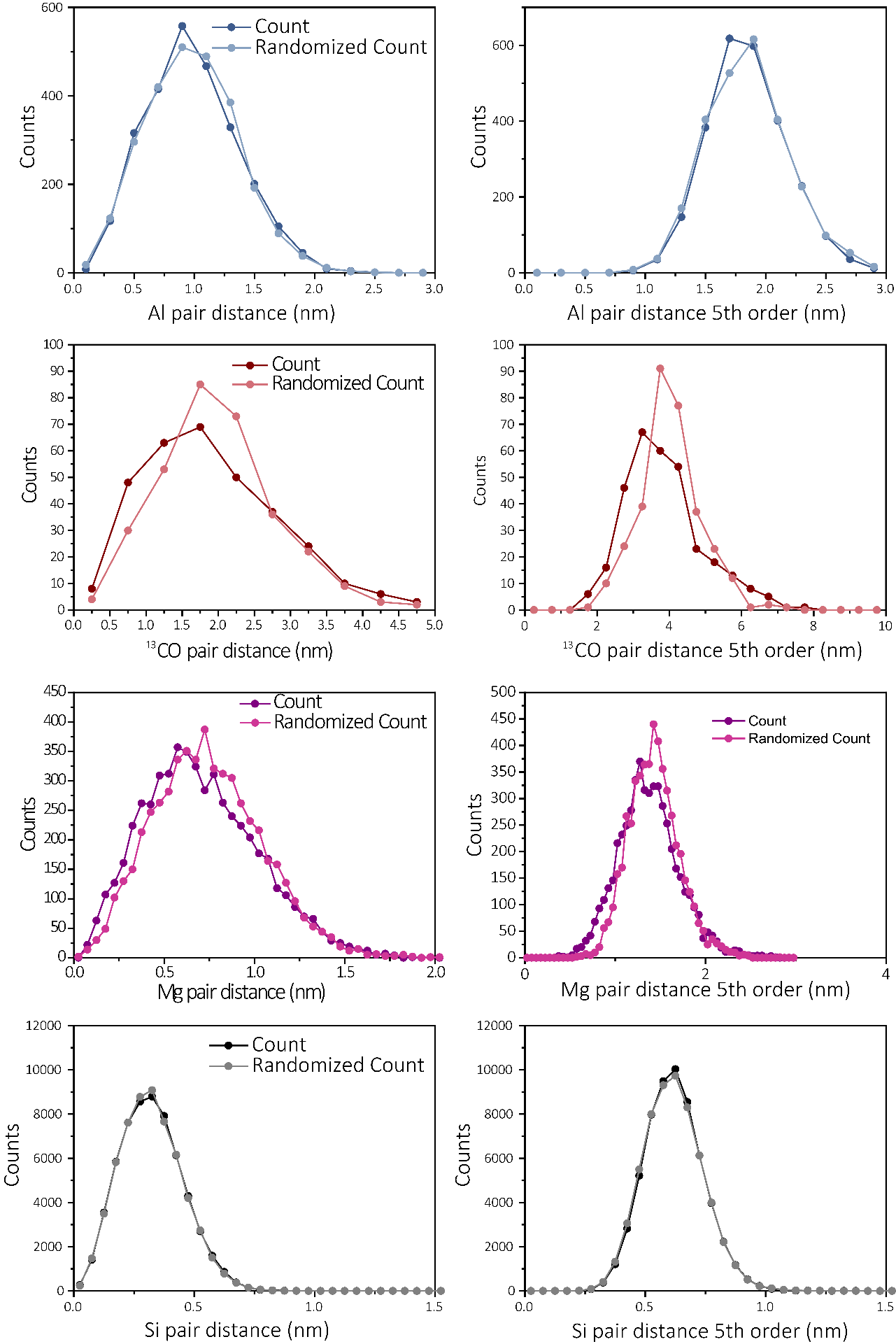


**Figure S19:** Nearest neighbour distribution (NND) of different elements of spent Mg-SZZ-13 30-min coked sample dataset 1.


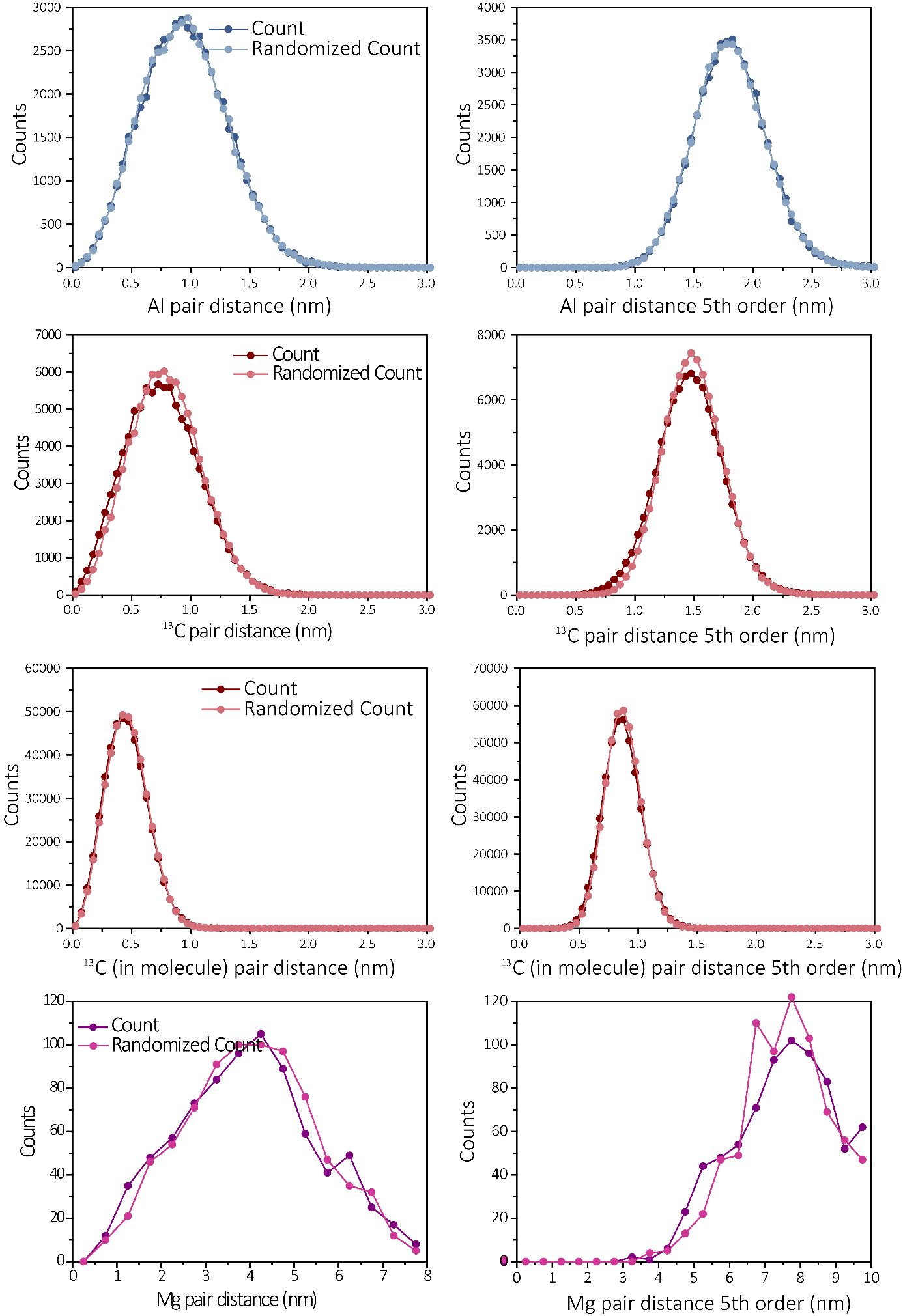


**Figure S20**: Nearest neighbour distribution (NND) of different elements of spent Mg-SZZ-13 30-min coked sample dataset 2.


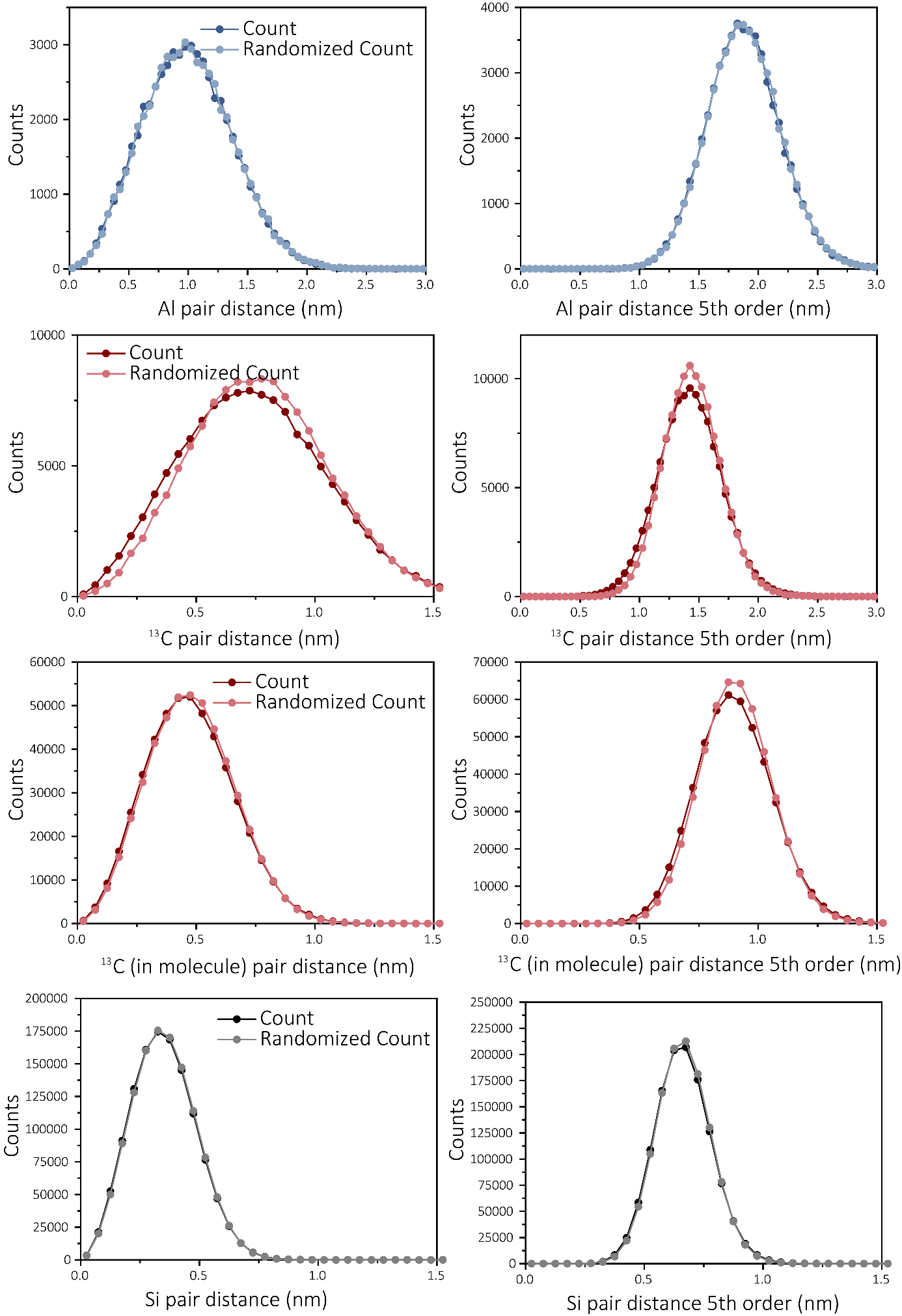


**Figure S21**: Nearest neighbour distribution (NND) of different elements of spent Mg-SZZ-13 60-min coked sample dataset 1.

**Table S8**: Pearson coefficient of Si, Mg and ^13^C. Pearson coefficient of Si has a value very close to zero, which indicates a complete homogeneous distribution while the Pearson coefficient of Mg and ^13^C is higher indicating a more heterogeneous distribution.

|  | |  | Pearson coefficient Si | Pearson coefficient Mg | Pearson coefficient ^13^C |
| --- | --- | --- | --- | --- | --- |
| Mg-SSZ-13 calcined | Dataset 1 | | 0.08 | - | - |
|  | | Dataset 2 | 0.02 | 0.47 | - |
|  | | Dataset 3 | 0.01 | 0.90 | - |
|  | |  |  |  |  |
| 1 min | | Dataset 1 | 0.02 | 0.38 | 0.49 |
|  | | Dataset 2 | 0.01 | - | 0.08 |
|  | |  |  |  |  |
| 15 min | | Dataset 1 | 0.03 | 0.29 | 0.18 |
|  | |  |  |  |  |
| 30 min | | Dataset 1 | 0.03 | 0.22 | 0.29 |
|  | | Dataset 2 | 0.03 | 0.18 | 0.04 |
|  | |  |  |  |  |
| 60 min | | Dataset 1 | 0.02 | - | 0.15 |


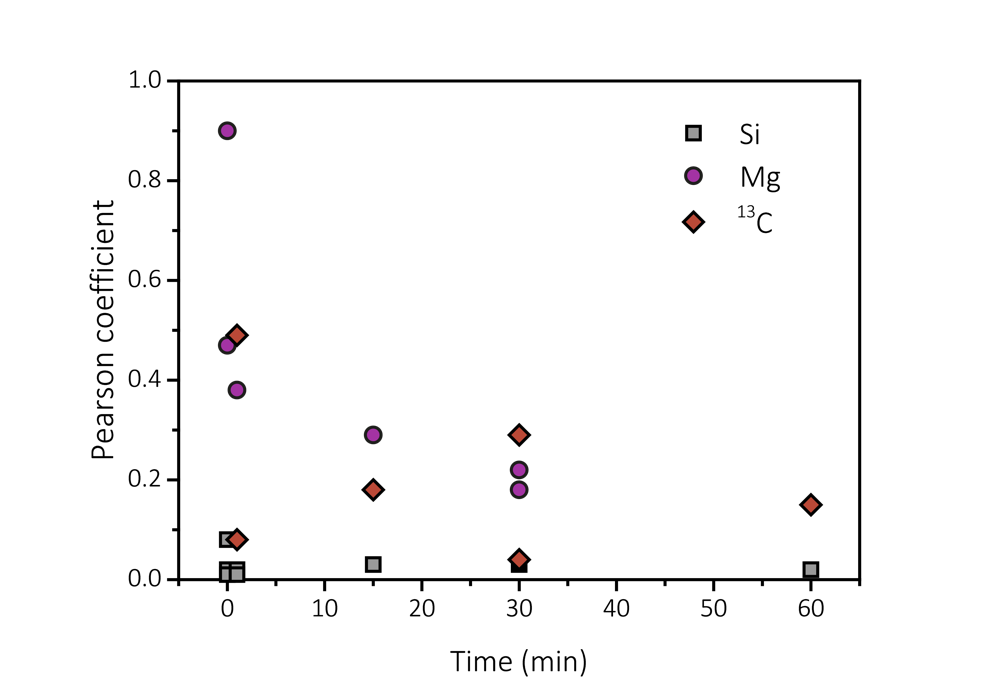


**Figure S22:** Pearson coefficient of the nearest neighbor distributions of Si, Mg and 13C with different time-on-stream.

### Radial Distribution Function Analysis of the Spent MgSSZ-13 Sample


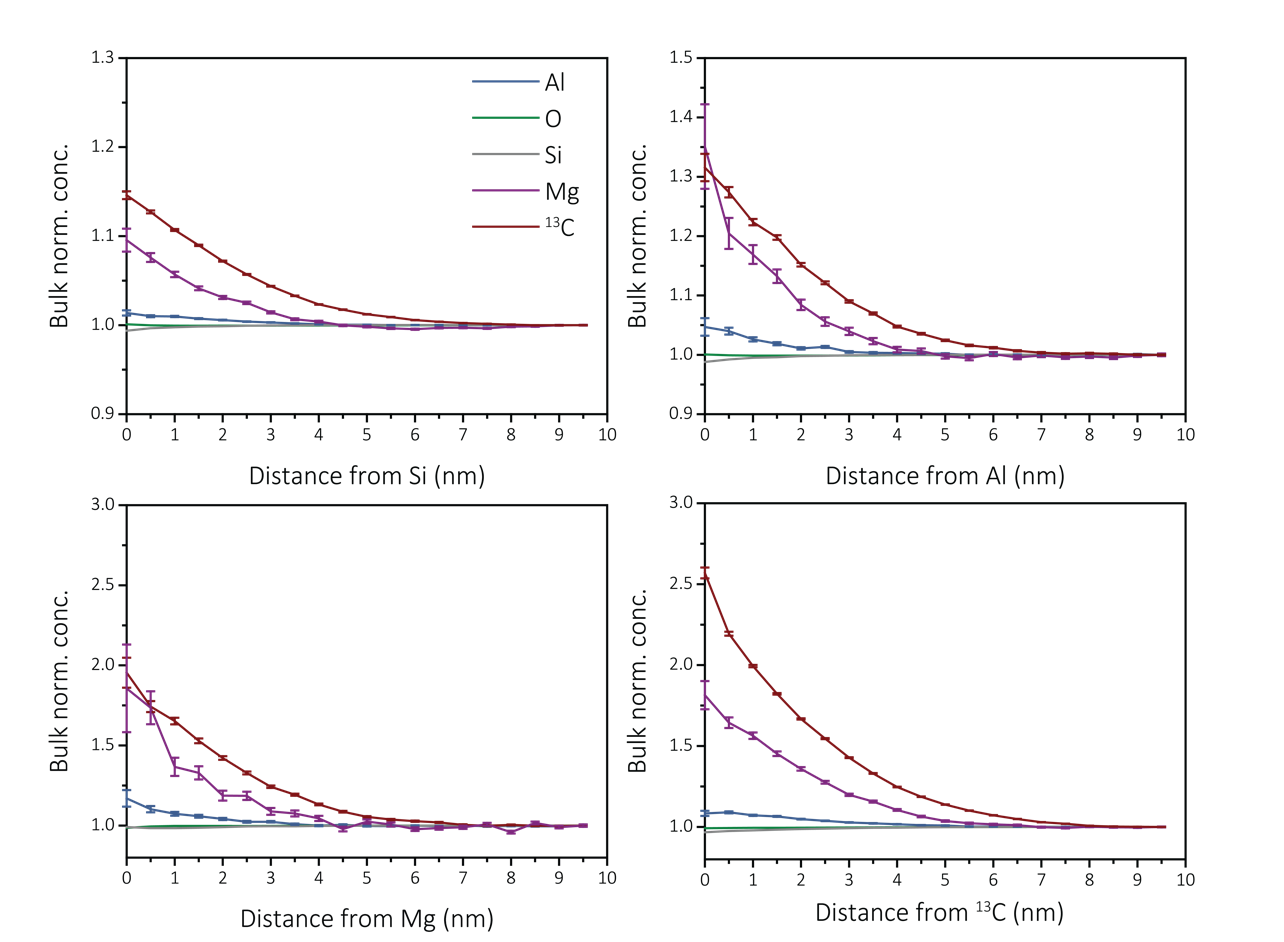


**Figure S23**: Radial distribution function (RDF) analysis of spent Mg-SSZ-13 coked 1 min dataset 1.


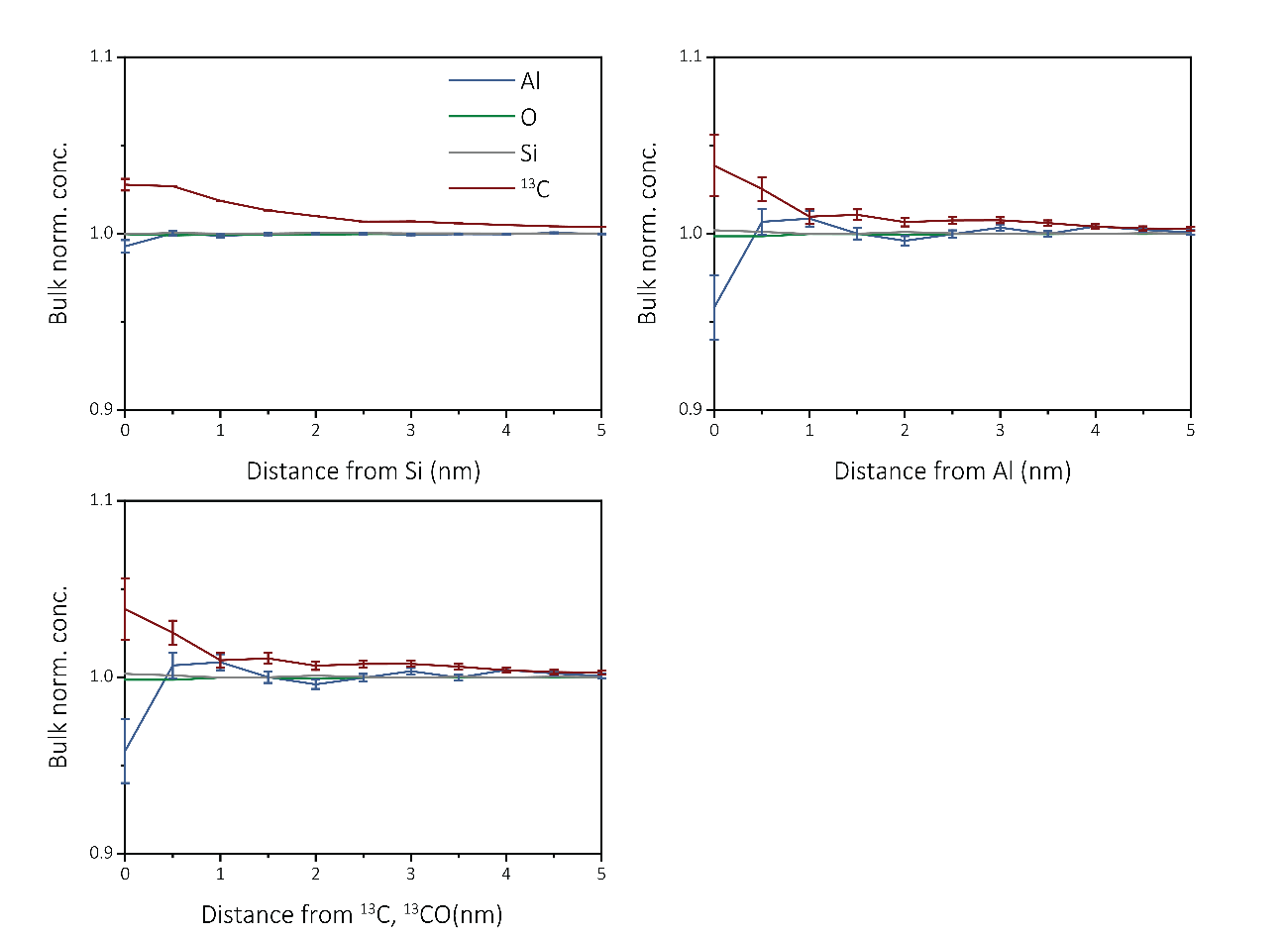


**Figure S24**: Radial distribution function (RDF) analysis of spent Mg-SSZ-13 coked 1-min dataset 2.


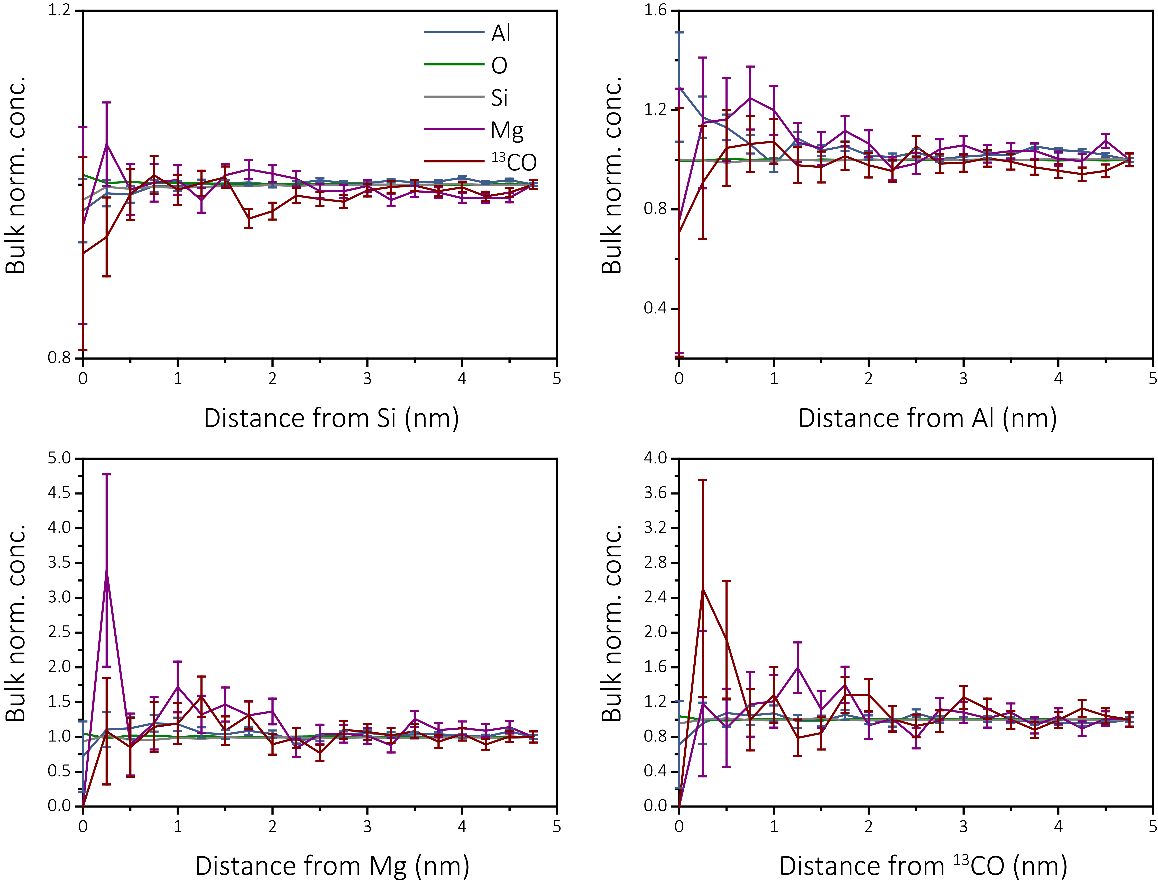


**Figure S25**: Radial distribution function (RDF) analysis of spent Mg-SSZ-13 coked 15-min dataset 1.


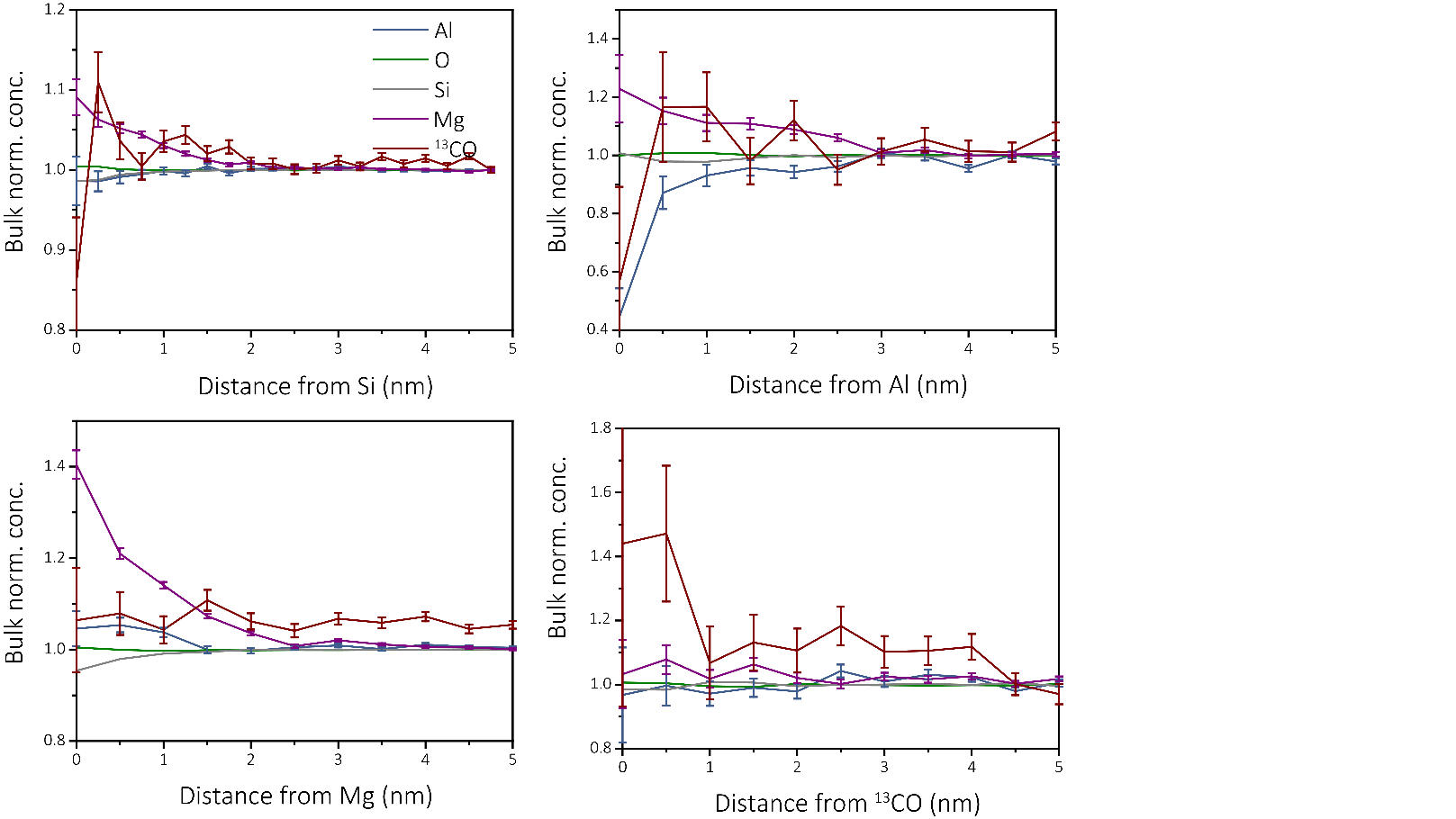


**Figure S26**: Radial distribution function (RDF) analysis of spent Mg-SSZ-13 coked 30-min dataset 1.


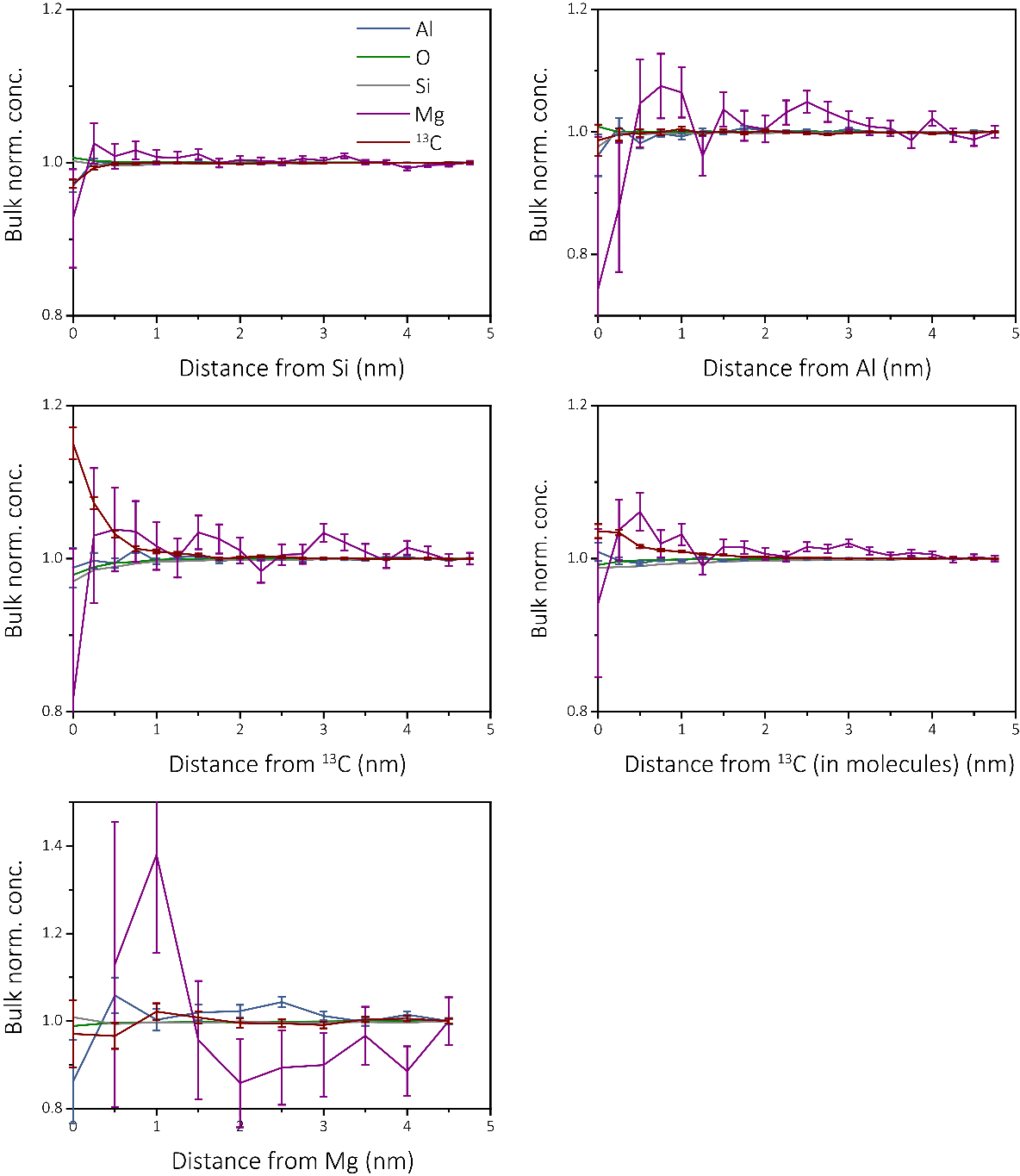


**Figure S27**: Radial distribution function (RDF) analysis of spent Mg-SSZ-13 coked 30-min dataset 2.


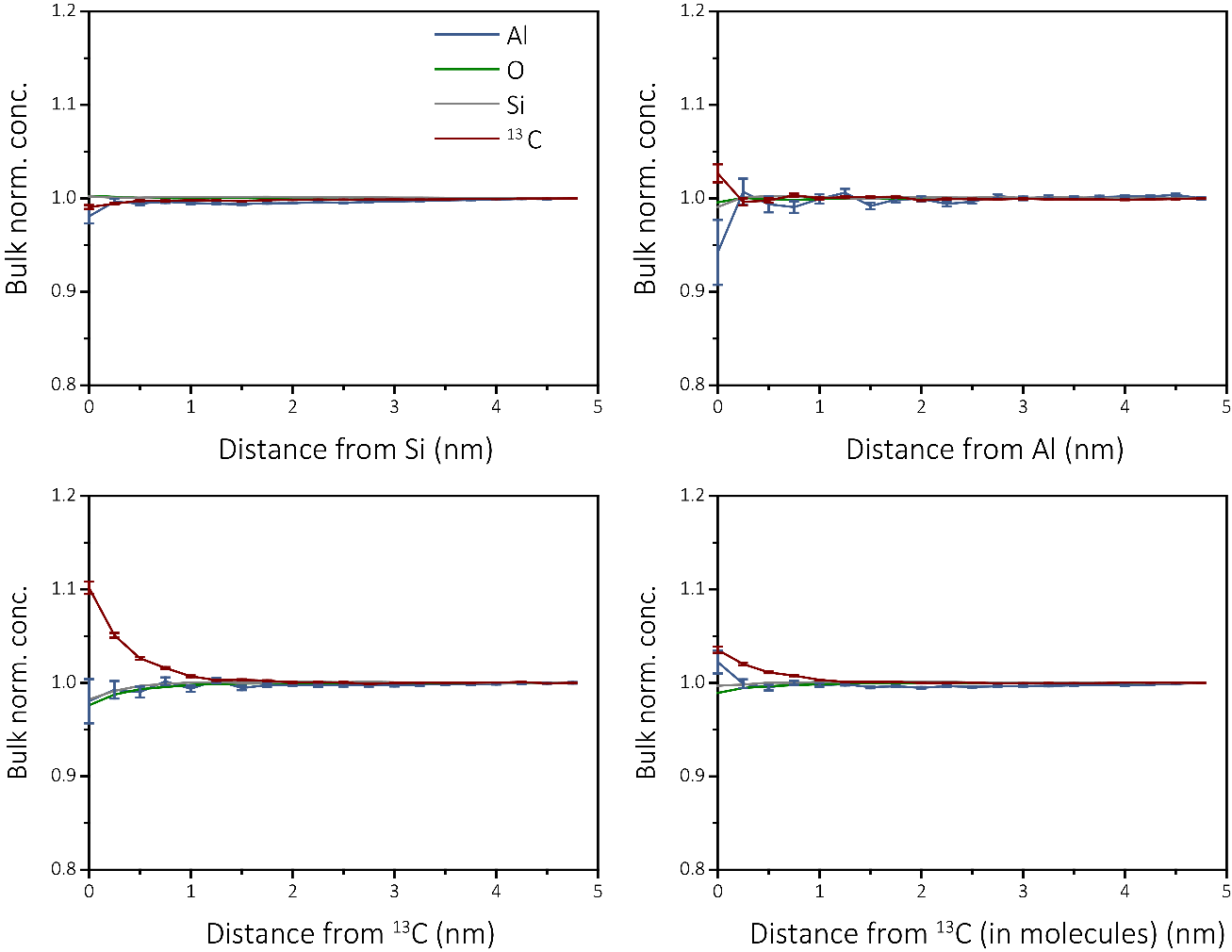


**Figure S28**: Radial distribution function (RDF) analysis of spent Mg-SSZ-13 coked 60-min dataset 1.


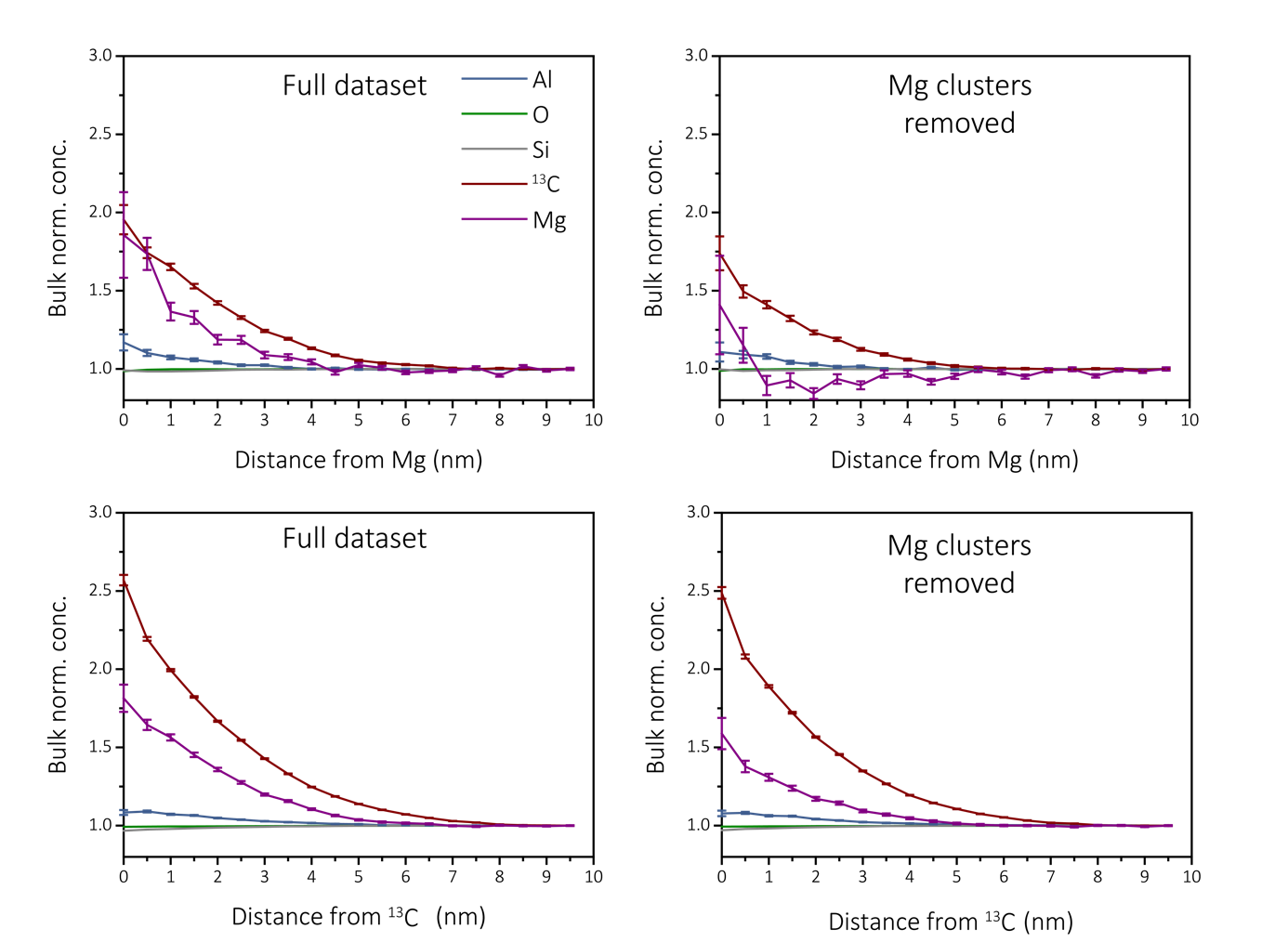


**Figure S29**: Radial distribution function (RDF) analysis of spent Mg-SSZ013 coked 1 min dataset 1 of Mg and ^13^C with and without Mg clusters.

### Iso-surface Analysis of the Spent Mg-SSZ-13 Zeolite


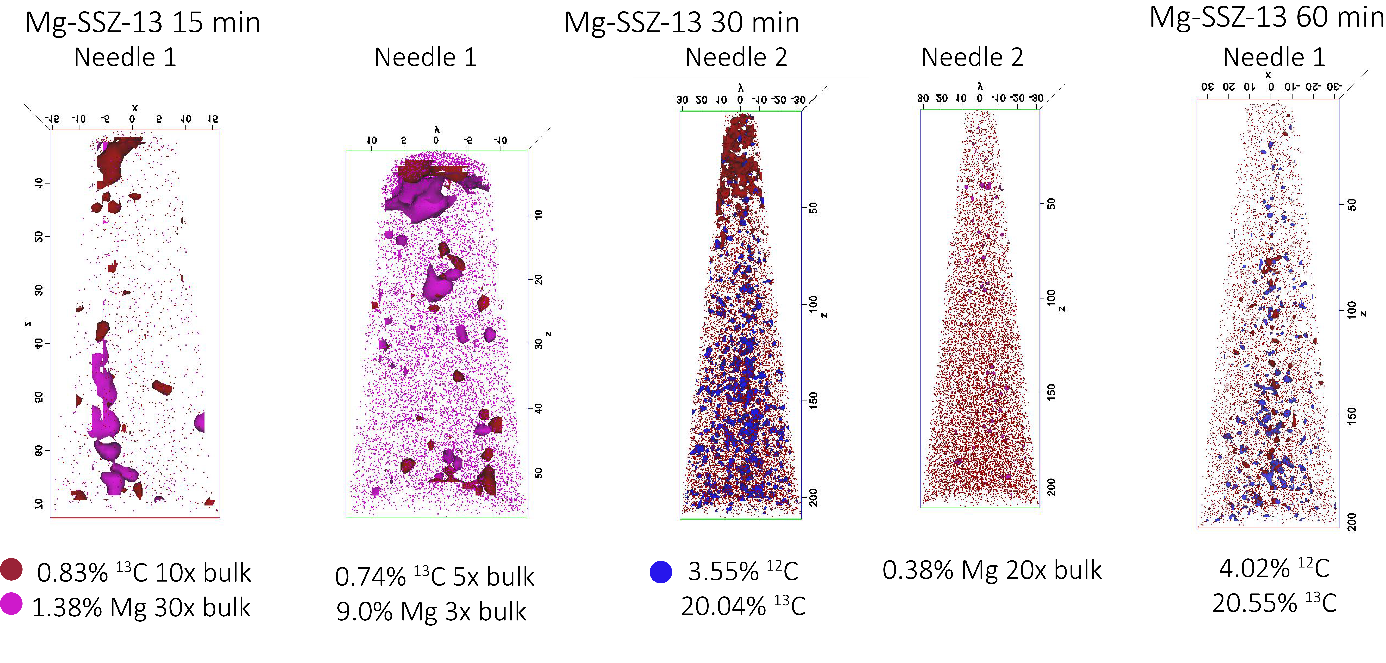


**Figure S30**: Iso-surface analysis of carbon and magnesium in Mg-SSZ-13 15 min coked dataset 1, with different concentrations, Mg-SSZ-13 30 min coked dataset 1 and 2 and Mg-SSZ-13 60-min coked sample.

# Catalytic Performance

## *Operando* UV-Vis Diffuse Reflectance Spectroscopy


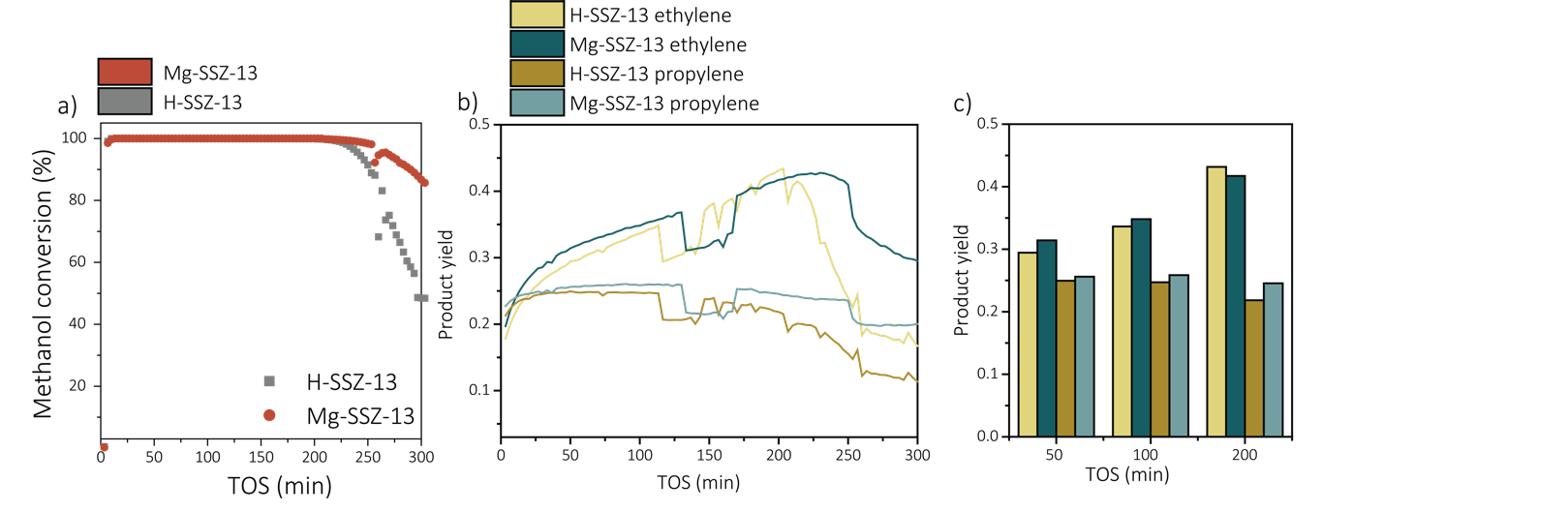


**Figure S31**: Catalytic performance results obtained during the methanol-to-hydrocarbons (MTH) reaction measured with *operando* UV-Vis diffuse reflectance spectroscopy (DRS). a) Methanol conversion measured with time-on-stream (TOS), b) product yield of ethylene and propylene with TOS and a c) different representation of the product yield with TOS. Between 120-150 min there was a pressure drop in which the carrier gas He-flow dropped for about 20 min.

## Different methods are available and already used in our research group to analyze the UV-Vis diffuse reflectance spectra and to quantify the growth of certain UV-Vis absorption bands overtime.^3,4,2,5,6^ However, due to the extensive coking of the SSZ-13 zeolite material, it is quite hard and somehow arbitrary to correct for the spectral contribution of the coking or “blackening” of the material by subtracting this from the data. Therefore, in this research we have opted for a different approach in which we only look into the intensity of the absorbance bands at certain wavelengths overtime. We think that this approach can be used to find trends in the formation of hydrocarbon intermediates as no peak shifts are observed over time (**Figure S32c** and **d**). As the absorption bands in the UV-Vis diffuse reflectance spectra are not deconvoluted, the exact quantity of the aromatic hydrocarbons in the zeolite cages cannot be determined, but we would like to draw some conclusions about the trends we observe. Because the precise knowledge of the intermediate hydrocarbons is not the aim of this study, we think that this approach, in which only indicating formation rates do justify trends are used, in satisfying enough.

The evolution rates can also be normalized resulting in further information about the evolution of the absorbance intensities over time allowing further analysis. The initial formation rate of the neutral, charged mono-alkylated benzenes and the charged poly-alkylated benzenes, which are active MTH intermediates, is decreasing drastically with the introduction of magnesium. This means that, just as for zeolite ZSM-5, magnesium could also suppress the aromatic cycle in the chabazite structure. The initial formation of poly aromatic compounds, which act as deactivating compounds, is not altered by the introduction of magnesium. This could be an indication of the existence of a similar dual cycle mechanism as for ZSM-5, as these results, even though less pronounced for SSZ-13, are also found for the ZSM-5 zeolite. This effect has been explained by the decrease in Brønsted acid sites, which also is observed for SSZ-13, which limits the formation of coke by olefin aromatization and the suppression of the aromatic cycle.


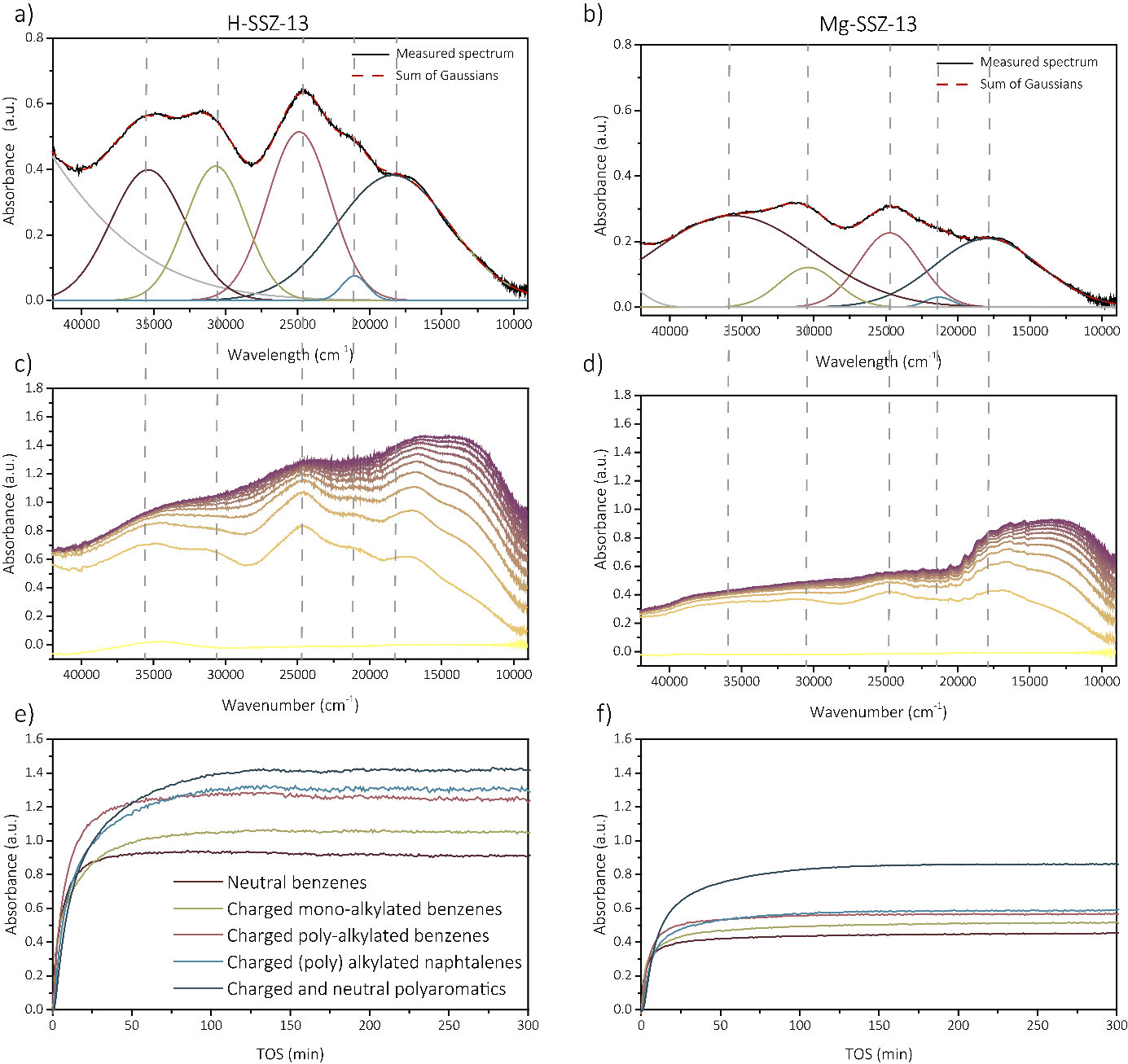


**Figure S32**: a) The fitted UV-Vis diffuse reflectance spectrum for 5 min time-on-stream (TOS) of H-SSZ-13, b) the fitted UV-Vis diffuse reflectance spectrum for 5 min TOS of the Mg-SSZ, c) the UV-vis spectra over time of H-SSZ-13, d) the UV-vis spectra over time of Mg-SSZ-13 e) the evolution of the absorption with TOS corresponding to the wavenumbers determined with the spectral fitting of H-SSZ-13 5 min 13, and f) the evolution of the absorption with time-on-stream corresponding to the wavenumbers determined with the spectral fitting of zeolite Mg-SSZ-13.

To conclude, the introduction of magnesium into the SSZ-13 structure seems to reduce the amount of aromatic species due to the MTH reaction. The formation of less aromatic intermediate species can be linked to the increased propylene selectivity and the decreased deactivation rate of the catalyst. Additionally, the active aromatic species, which do form, seems to form less rapidly. This can be attributed to the decrease in Bronsted acid sites, which decreases the formation of aromatic molecules by the suppression of olefin aromatization.

##
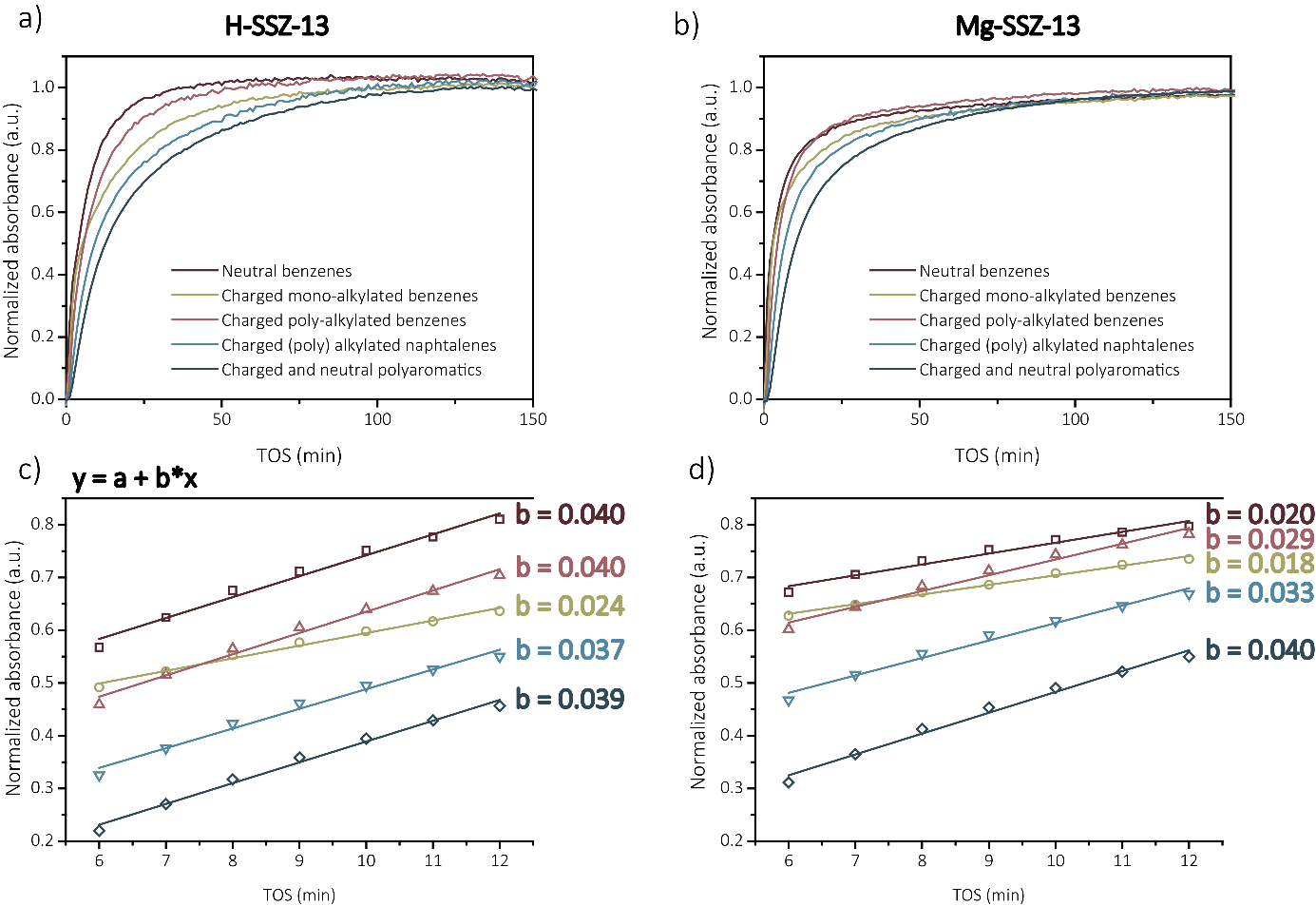
*Operando* X-ray Diffraction

**Figure S 33**: The normalized absorbance of different aromatic intermediates and deactivating compounds in H-SSZ-13 corresponding to the spectral evolution at certain wavenumbers determined with the spectral fitting of the 5 min time-on-stream (TOS) UV- Vis diffuse reflectance spectra of a) H-SSZ-13 and b) Mg-SSZ-13. The normalized absorbance between 6 and 12 min TOS to determine the initial formation rates of the different aromatic compounds in c) H-SSZ-13 and d) Mg-SSZ-13.


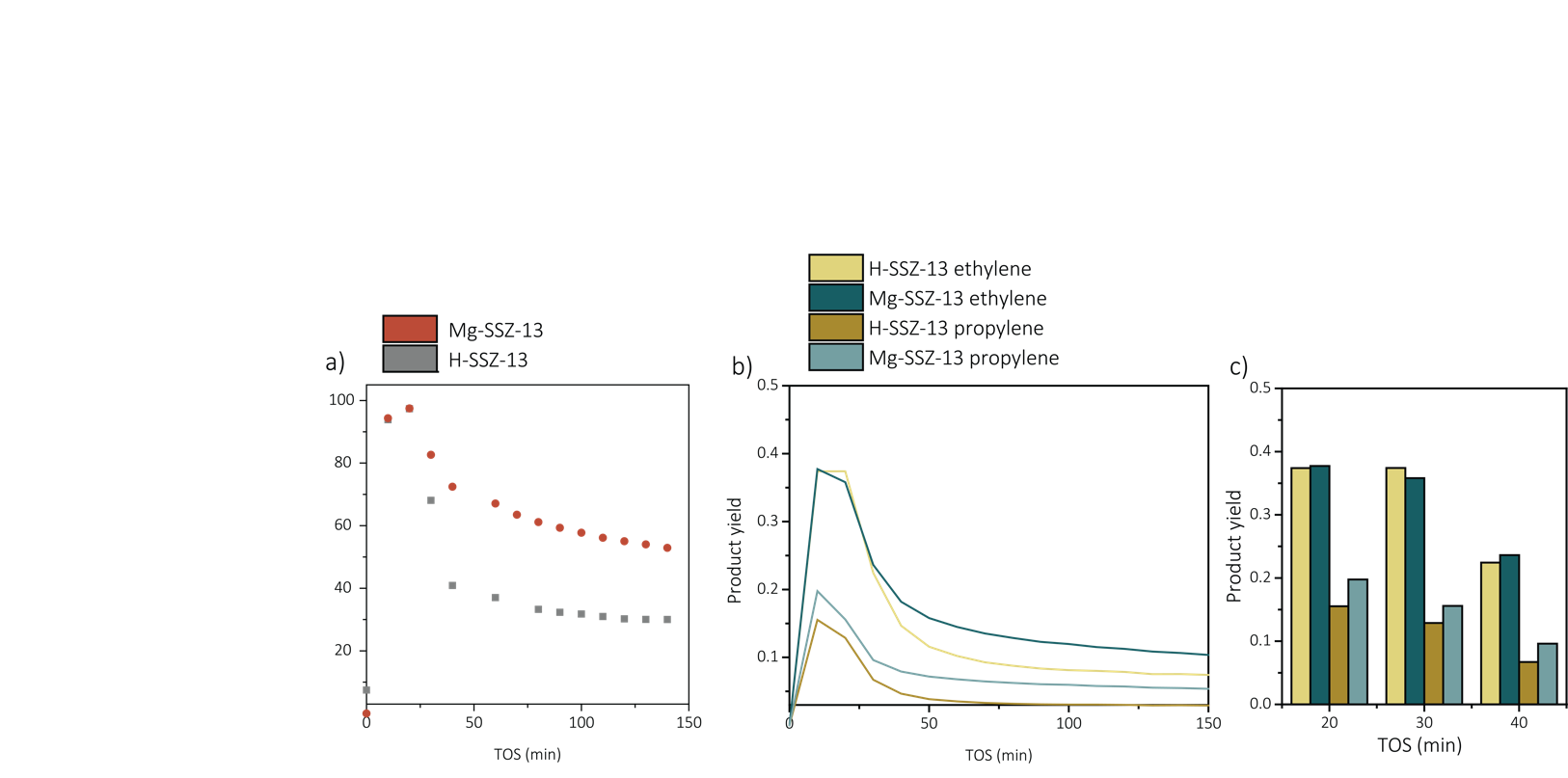


**Figure S34**: Catalytic performance results obtained during the methanol-to-hydrocarbons (MTH) reaction measured with *operando* X-ray diffraction (XRD). a) Methanol conversion measured with time-on-stream (TOS), b) product yield of ethylene and propylene with TOS and a c) different representation of the product yield with TOS.

The *operando* XRD patterns are shown in **Figure S35a** and **c** in a waterfall plot. To show the peak shifts over time they are also depicted as contour plots. For clarity, we have zoomed in between 11.5 and 12.5 2θ ° as this area shows a reflection which does not change with TOS as well as a peak that shifts to lower angles, which is related to the expansion of the crystal lattice, notably faster for the H-SSZ-13 than for the Mg-SSZ-13 catalysts. As the lattice expansion of the crystal is widely accepted to be related to the formation of coke molecules, the less rapid growth of the crystal lattice can be related to the slower formation of aromatic intermediates in Mg-SSZ-13.


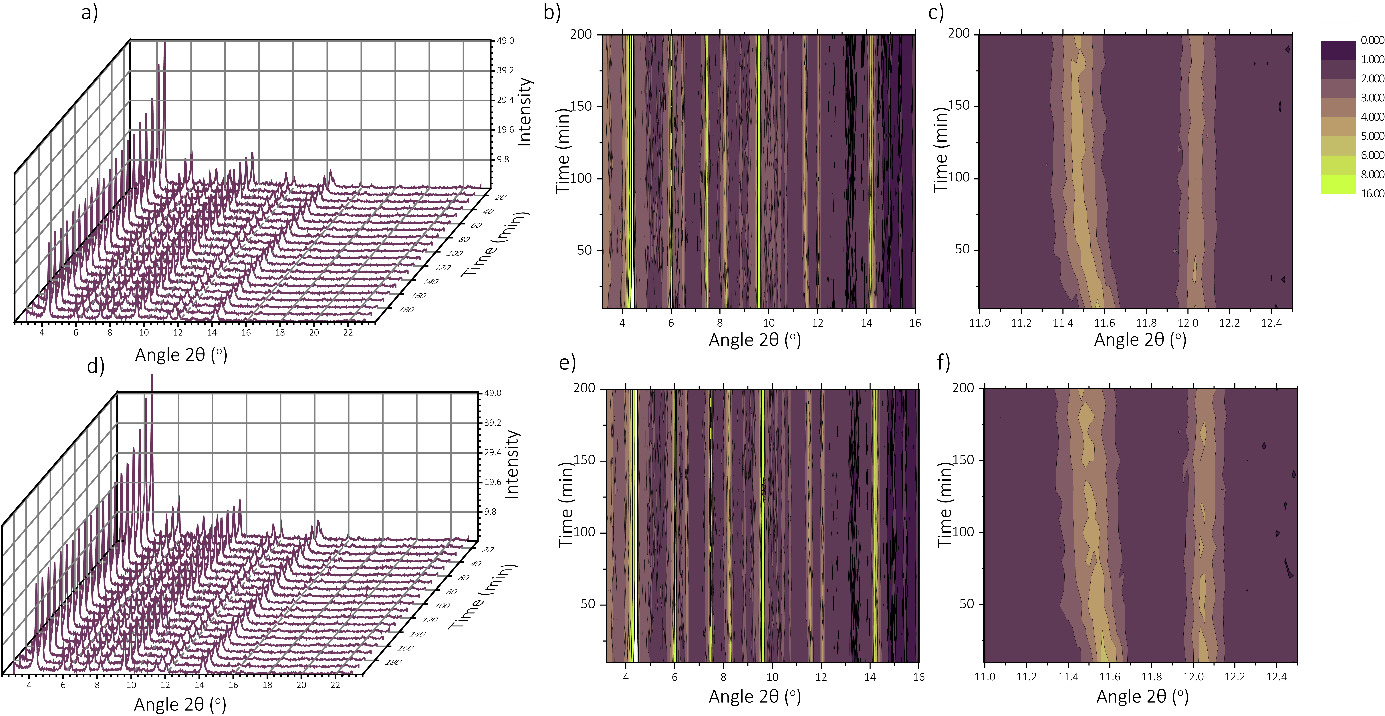


**Figure S35**: *Operando* X-ray diffraction (XRD) results under methanol-to-hydrocarbons (MTH) conditions over H-SSZ-13 (a,b,c) and Mg-SSZ-13 (c, e, f) with a) and d) as the waterfall plot of the *operando* XRD data, b) and e) the contour plots between 3.2 and 23 2θ ° and c) and f) the contour plots between 11 and 12.5 2θ ° to highlight the peak shift.

The lattice expansion of the zeolite in the c-direction, linearly related to the peak shift to lower angles at certain 2θ, has been calculated with the use of Rietveld refinement. By plotting the c-direction lattice dimension over time-on-stream, we observed that the lattice expansion is indeed faster for H-SSZ-13 than for Mg-SSZ-13. However, even after deactivation, the Mg-SSZ-13 keeps expanding, which could mean that after deactivation the trapped hydrocarbons in the Mg-SSZ-13 keep growing resulting in further lattice expansion.

The structure which was used to fit the *operando* X-ray diffractograms is described in **Table S9**. The dimensions, the Rwp and the peak position of the [104] reflection are listed in **Table S10** and **Table S11**. Some fits are depicted in **Figure S35** and **Figure S36** to show the goodness of fit. Additionally, to show that the lattice expansion can also be described by the shift of certain reflections, **Figure S36** depicted the lattice parameters in the c-direction as well as the peak position of the [104] reflection over time.

**Table S9**: Structure information of PDF card - 04-019-7189 with added C atom.

| Site | Np | x | y | z | Atom | Occ | Beq |
| --- | --- | --- | --- | --- | --- | --- | --- |
| Si_1 | 36 | 0.450(2) | 0.023(2) | 0.224(2) | Si | 0.982 | 1 |
| Al_1 | 36 | 0.14(9) | 0.6(8) | 1.46(5) | Al | 0.046 | 1 |
| O_1 | 18 | 1.559(5) | -0.559(5) | -0.417(8) | O | 1 | 1 |
| O_2 | 18 | 1.757(6) | 1.091(6) | 0.16667 | O | 1 | 1 |
| O_3 | 18 | -0.784(3) | -1.567(7) | 1.098(6) | O | 1 | 1 |
| O_4 | 18 | 0.00000 | -2.046(4) | 0.00000 | O | 1 | 1 |
| C_2 | 18 | 0.9593(9) | 0.9186(18) | 0.1232(10) | C | 6.7(3) | 1 |

**Table S10**: The lattice dimensions in the a=b and c directions, the error of the dimension in the c-directions, the Rwp factor and the peak position of the [104] reflection of H-SSZ-13.

| Time | a=b (Å) | c (Å) | Error c (Å) | Rwp-factor | Peak position [104] |
| --- | --- | --- | --- | --- | --- |
| 10 | 13.540 | 14.778 | 0.0119 | 18.64 | 11.56 |
| 20 | 13.546 | 14.783 | 0.0068 | 13.86 | 11.57 |
| 30 | 13.549 | 14.819 | 0.0074 | 13.75 | 11.53 |
| 40 | 13.549 | 14.854 | 0.0080 | 12.82 | 11.51 |
| 50 | 13.547 | 14.864 | 0.0079 | 15.88 | 11.50 |
| 60 | 13.565 | 14.910 | 0.0087 | 14.23 | 11.48 |
| 70 | 13.575 | 14.928 | 0.0069 | 12.93 | 11.48 |
| 80 | 13.580 | 14.938 | 0.0080 | 12.85 | 11.47 |
| 90 | 13.579 | 14.949 | 0.0088 | 14.73 | 11.47 |
| 100 | 13.581 | 14.949 | 0.0084 | 12.84 | 11.46 |
| 110 | 13.564 | 14.938 | 0.0073 | 12.61 | 11.46 |
| 120 | 13.555 | 14.930 | 0.0076 | 12.87 | 11.46 |
| 130 | 13.563 | 14.939 | 0.0070 | 12.65 | 11.46 |
| 140 | 13.578 | 14.954 | 0.0053 | 12.67 | 11.46 |
| 150 | 13.572 | 14.955 | 0.0082 | 12.90 | 11.46 |
| 160 | 13.577 | 14.964 | 0.0078 | 13.18 | 11.46 |
| 170 | 13.559 | 14.938 | 0.0078 | 12.97 | 11.46 |
| 180 | 13.567 | 14.952 | 0.0074 | 12.83 | 11.46 |
| 190 | 13.576 | 14.959 | 0.0075 | 12.73 | 11.46 |
| 200 | 13.550 | 14.933 | 0.0075 | 12.52 | 11.46 |
| 210 | 13.551 | 14.938 | 0.0083 | 12.55 | 11.46 |
| 220 | 13.568 | 14.953 | 0.0085 | 12.54 | 11.46 |
| 230 | 13.568 | 14.958 | 0.0073 | 12.68 | 11.46 |
| 240 | 13.573 | 14.964 | 0.0079 | 12.65 | 11.46 |
| 250 | 13.577 | 14.967 | 0.0072 | 12.61 | 11.45 |
| 260 | 13.554 | 14.944 | 0.0087 | 13.19 | 11.46 |
| 270 | 13.557 | 14.948 | 0.0079 | 12.65 | 11.46 |
| 280 | 13.537 | 14.925 | 0.0082 | 12.73 | 11.46 |
| 290 | 13.557 | 14.947 | 0.0077 | 12.10 | 11.46 |
| 300 | 13.555 | 14.946 | 0.0077 | 11.97 | 11.46 |
| 310 | 13.574 | 14.972 | 0.0077 | 12.63 | 11.45 |
| 320 | 13.568 | 14.955 | 0.0093 | 12.89 | 11.46 |
| 330 | 13.552 | 14.941 | 0.0086 | 12.94 | 11.46 |
| 340 | 13.572 | 14.967 | 0.0083 | 12.49 | 11.46 |
| 350 | 13.563 | 14.956 | 0.0081 | 13.08 | 11.46 |

**Table S11**: The lattice dimensions in the a=b and c directions, the error of the dimension in the c-directions, the Rwp factor and the peak position of the [104] reflection of Mg-SSZ-13.

| Time | a=b (Å) | c (Å) | Error c (Å) | Rwp-factor | Peak position [104] |
| --- | --- | --- | --- | --- | --- |
| 10 | 13.561 | 14.795 | 0.0053 | 13.07 | 11.58 |
| 20 | 13.556 | 14.796 | 0.0056 | 13.11 | 11.56 |
| 30 | 13.544 | 14.802 | 0.0060 | 12.53 | 11.56 |
| 40 | 13.519 | 14.789 | 0.0075 | 13.13 | 11.55 |
| 50 | 13.522 | 14.815 | 0.0073 | 12.58 | 11.53 |
| 60 | 13.524 | 14.827 | 0.0080 | 12.95 | 11.52 |
| 70 | 13.518 | 14.827 | 0.0081 | 13.14 | 11.52 |
| 80 | 13.518 | 14.843 | 0.0073 | 12.81 | 11.51 |
| 90 | 13.518 | 14.847 | 0.0086 | 12.95 | 11.51 |
| 100 | 13.517 | 14.848 | 0.0081 | 13.09 | 11.50 |
| 110 | 13.518 | 14.850 | 0.0083 | 12.57 | 11.50 |
| 120 | 13.519 | 14.855 | 0.0080 | 12.62 | 11.50 |
| 130 | 13.521 | 14.865 | 0.0083 | 13.36 | 11.49 |
| 140 | 13.522 | 14.881 | 0.0064 | 13.12 | 11.49 |
| 150 | 13.529 | 14.886 | 0.0086 | 12.79 | 11.48 |
| 160 | 13.519 | 14.882 | 0.0078 | 12.57 | 11.48 |
| 170 | 13.518 | 14.882 | 0.0078 | 12.67 | 11.48 |
| 180 | 13.519 | 14.888 | 0.0077 | 12.25 | 11.48 |
| 190 | 13.555 | 14.928 | 0.0072 | 12.74 | 11.48 |
| 200 | 13.517 | 14.889 | 0.0079 | 12.86 | 11.47 |
| 210 | 13.522 | 14.894 | 0.0075 | 12.86 | 11.48 |
| 220 | 13.518 | 14.889 | 0.0081 | 13.06 | 11.48 |
| 230 | 13.512 | 14.894 | 0.0076 | 12.80 | 11.48 |
| 240 | 13.518 | 14.891 | 0.0078 | 12.45 | 11.48 |
| 250 | 13.542 | 14.930 | 0.0070 | 12.51 | 11.47 |
| 260 | 13.519 | 14.902 | 0.0079 | 12.70 | 11.47 |
| 270 | 13.518 | 14.899 | 0.0049 | 12.94 | 11.46 |
| 280 | 13.515 | 14.898 | 0.0072 | 17.75 | 11.47 |
| 290 | 13.532 | 14.914 | 0.0074 | 12.65 | 11.47 |
| 300 | 13.558 | 14.956 | 0.0074 | 12.77 | 11.46 |
| 310 | 13.517 | 14.902 | 0.0077 | 12.37 | 11.46 |
| 320 | 13.554 | 14.955 | 0.0069 | 12.73 | 11.46 |
| 330 | 13.553 | 14.950 | 0.0068 | 12.34 | 11.46 |
| 340 | 13.517 | 14.912 | 0.0076 | 12.22 | 11.46 |
| 350 | 13.518 | 14.916 | 0.0075 | 12.40 | 11.46 |


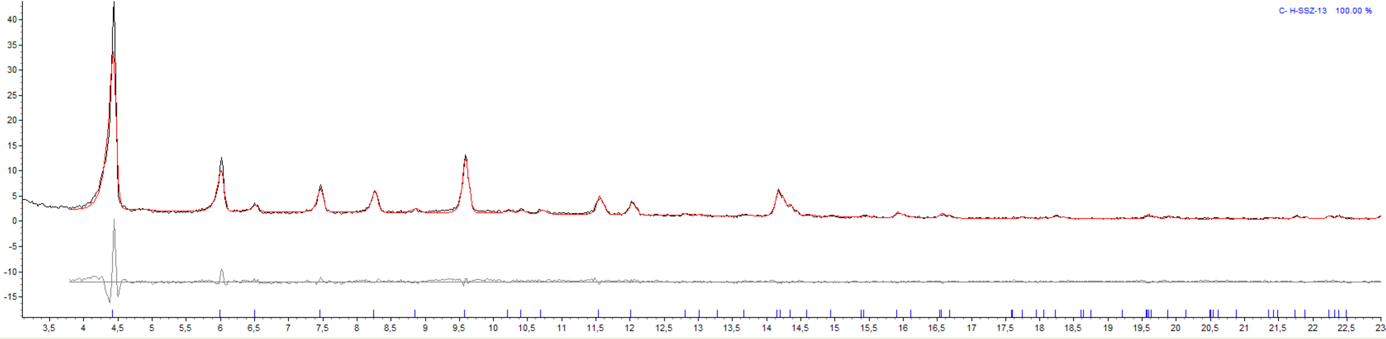


**Figure S36**: Example of a refined diffractogram of H-SSZ-13 collected under *operando* conditions.


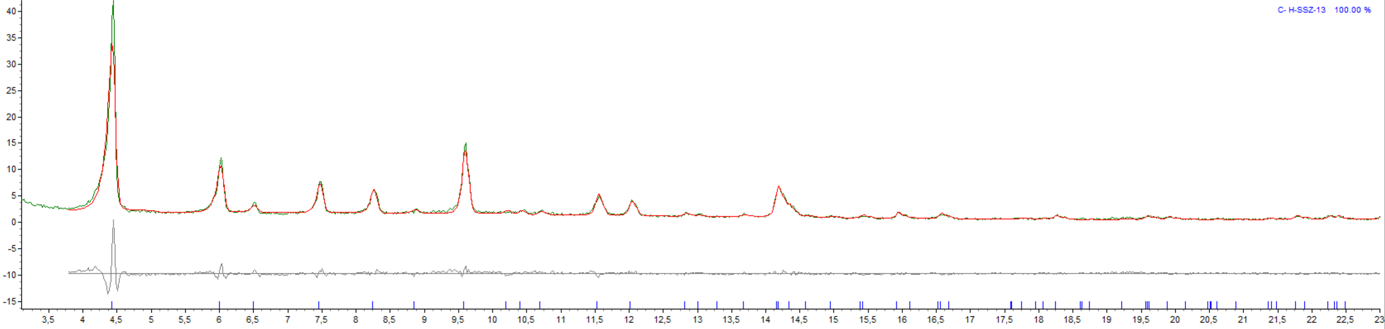


**Figure S37**: Example of a refined diffractogram of Mg-SSZ-13 collected under *operando* conditions.


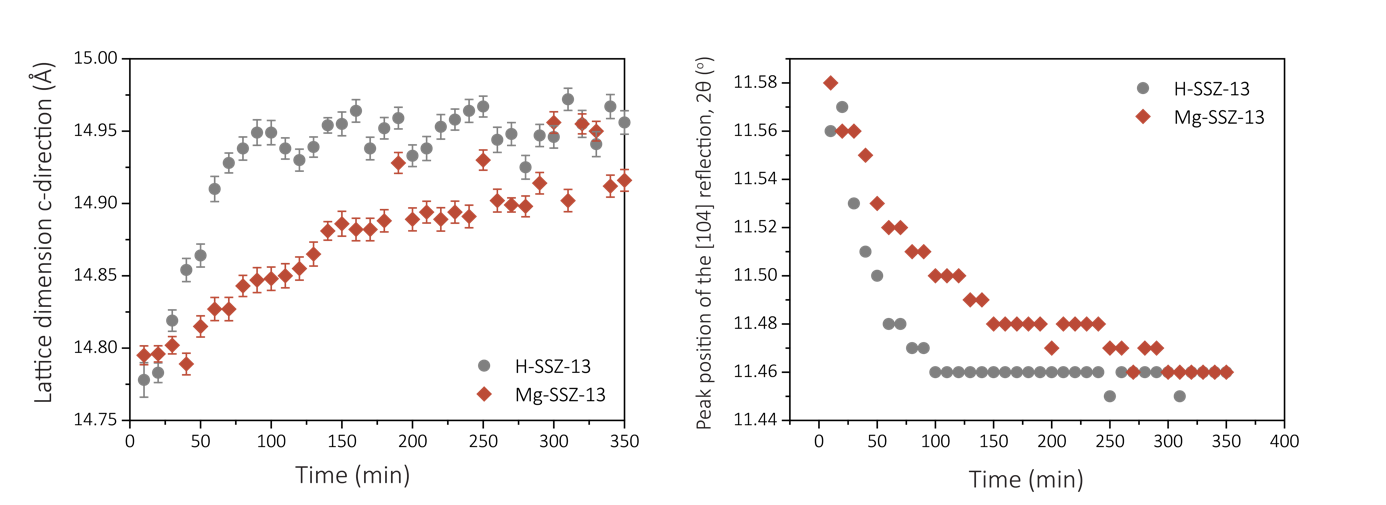


**Figure S38**: Dimensions of the lattice in the c-direction determined with Rietveld refinement analysis and the peak position of the [104] reflection, which shifts due to the expansion, with increasing time-on-stream of zeolite H-SSZ-13 and Mg-SSZ-13 during the methanol-to-hydrocarbons (MTH) reaction (WHSV = 0.7 h^-1^, 450 °C).

**References**

1 R. Oord, I. C. ten Have, J. M. Arends, F. C. Hendriks, J. Schmidt, I. Lezcano-Gonzalez and B. M. Weckhuysen, *Catal. Sci. Technol.*, 2017, **7**, 3851–3862.

2 J. Goetze, F. Meirer, I. Yarulina, J. Gascon, F. Kapteijn, J. Ruiz-mart and B. M. Weckhuysen, *ACS Catal.*, 2017, **7**, 4033–4046.

3 J. Goetze and B. M. Weckhuysen, *Catal. Sci. Technol.*, 2018, **8**, 1632–1644.

4 J. Goetze, I. Yarulina, J. Gascon, F. Kapteijn and B. M. Weckhuysen, *ACS Catal.*, 2018, **8**, 2060–2070.

5 E. Borodina, F. Meirer, I. Lezcano-González, M. Mokhtar, A. M. Asiri, S. A. Al-Thabaiti, S. N. Basahel, J. Ruiz-Martinez and B. M. Weckhuysen, *ACS Catal.*, 2015, **5**, 992–1003.

6 E. Borodina, H. Sharbini Harun Kamaluddin, F. Meirer, M. Mokhtar, A. M. Asiri, S. A. Al-Thabaiti, S. N. Basahel, J. Ruiz-Martinez and B. M. Weckhuysen, *ACS Catal.*, 2017, **7**, 5268–5281.

7 Y. Ono, H. Adachi and Y. Senoda, *J. Chem. Soc. Faraday Trans. 1*, 1988, **84**, 1091–1099.
